# Metabolic reprogramming in cancer: dysregulation of glucose, lipid, and amino acid pathways and therapeutic opportunities

**DOI:** 10.1186/s43556-026-00427-2

**Published:** 2026-03-10

**Authors:** Mingkang Yu, Di Yang, Xiuli Chen, Yuling Yang, Bingqiang Zhang, Xinxin Jiang, Lijie Xing, Yuxuan Yang, Yani Sun, Ning Li

**Affiliations:** 1 School of Basic Medicine, Qingdao Medical College, Qingdao University, Qingdao, China; 2 Department of Infectious Diseases, The Affiliated Hospital of Qingdao University, Qingdao University, Qingdao, China; 3 Qingdao Restore Biotechnology Co., Ltd., Qingdao, China; 4 Qingdao Engineering Research Center for Cellular Immunity and Early Cancer Screening, Qingdao, China

**Keywords:** Metabolic reprogramming, Hepatocellular carcinoma, Glycolysis, Lipogenesis, Glutamine, Metabolism-targeted therapy

## Abstract

Metabolic reprogramming is a hallmark of cancer, including hepatocellular carcinoma (HCC). Cancer cells exhibit enhanced glucose and glutamine uptake, increased glycolysis, pentose phosphate pathway activity, de novo lipogenesis, and altered amino acid metabolism. However, the metabolic crosstalk underlying cancer progression and the strategic directions for drug development remain insufficiently synthesized. This review systematically summarizes the functional mechanisms of key signaling regulators involved in cancer metabolic reprogramming, including mammalian target of rapamycin complex 1 (mTORC1), myelocytomatosis viral oncogene homolog (c-Myc), hypoxia-inducible factor-1α (HIF-1α), activating transcription factor 4 (ATF4), nuclear factor erythroid 2–related factor 2 (NRF2), and sterol regulatory element–binding protein 1 (SREBP1). Notably, we highlight the interconnections among metabolic pathways in cancer cells and the signaling hubs that orchestrate metabolic crosstalk, which together constitute an integrated network of metabolic pathways and their regulatory signals. Metabolic targets and metabolism-directed therapeutic agents with substantial developmental potential are comprehensively summarized, providing up-to-date insights and concrete directions for metabolism-targeted cancer therapy. Encouragingly, agents such as the fatty acid synthase inhibitor TVB-2640 and the glutaminase inhibitor CB-839 have already entered clinical trials. We recognize that adverse effects on normal tissues and drug resistance driven by metabolic plasticity represent major challenges for metabolism-targeted therapies. Accordingly, we systematically summarize innovative strategies that offer new therapeutic possibilities, including targeting multiple metabolic pathways through combination therapy to enhance efficacy, combining metabolic inhibitors to overcome resistance to conventional anticancer agents, leveraging metabolic reprogramming for early cancer detection, and exploring emerging approaches such as immunometabolism and metabolomics.

## Introduction

Malignant tumors represent a significant global public health challenge, with their high incidence and mortality rates posing a serious threat to human health [1]. Metabolic reprogramming has emerged as one of the core hallmarks of cancer, providing essential materials and energy to sustain uncontrolled proliferation and malignant progression of tumor cells [2]. Hepatocellular carcinoma (HCC) is the predominant form of primary liver cancer [3], ranking sixth among global malignancies [4] and serving as the third leading cause of cancer-related mortality [5, 6]. Major pathogenic factors for HCC encompass viral infections (hepatitis B virus, HBV; hepatitis C virus, HCV), chronic liver diseases (non-alcoholic fatty liver disease, NAFLD; non-alcoholic steatohepatitis, NASH), and environmental exposures (alcohol use [7] and aflatoxin ingestion [5]). HCC has an insidious onset and lacks typical early symptoms. As a result, 70% of patients are diagnosed at an advanced stage when clinical symptoms become apparent [8]. This delay leads to limited treatment options and a poor prognosis [5, 9, 10]. Given that the liver is the central organ for metabolism in the body, the occurrence and progression of HCC are inherently associated with severe metabolic disturbances. These disturbances make HCC an ideal model for studying cancer metabolic reprogramming [11].

The occurrence and progression of HCC are multi-stage evolutionary processes, with metabolic reprogramming being one of their core driving forces [12]. To meet the demands of rapid proliferation, invasion and metastasis, resistance to oxidative stress, and adaptation to the microenvironment, HCC cells undergo systematic remodeling of nutrient uptake and utilization pathways [13]. This remodeling is primarily reflected in the dysregulation of three core nutrient pathways: glucose metabolism [14], lipid metabolism [15], and amino acid metabolism [16]. The "Warburg effect" in glucose metabolism provides cells with rapid yet inefficient ATP, along with metabolic intermediates and precursors for phospholipid and nucleic acid synthesis [17]. Meanwhile, reprogrammed lipid metabolism (including uptake, de novo synthesis, and oxidation) supplies the building blocks for cell membrane construction and signal transduction [18]. Furthermore, amino acid metabolism, typified by glutamine, maintains the function of the tricarboxylic acid (TCA) cycle via anaplerosis and furnishes nitrogen sources [19]. Although research on specific metabolic pathways, such as glycolysis, has been relatively thorough in the past few decades [20–22], there are still significant limitations in current studies. For instance, the molecular networks underlying how the three major nutrient pathways crosstalk and how they jointly regulate the malignant phenotype of HCC remain unclear. Understanding these complex metabolic adaptations is crucial for developing novel therapeutic strategies for HCC [23], yet therapeutic translation targeting metabolism still faces significant challenges.

Against this backdrop, this review systematically summarizes the regulatory mechanisms and crosstalk underlying major metabolic reprogramming in cancer, using HCC as a paradigm. First, the characteristic alterations in three core nutrient pathways — glucose, lipid, and amino acid metabolism — in cancer and their impact on tumor biological behavior are reviewed. Subsequently, the complex crosstalk mechanisms among these metabolic pathways are highlighted, illustrating how they synergistically support tumor survival and adaptation. Finally, therapeutic opportunities and ongoing preclinical/clinical studies targeting these pathways are comprehensively evaluated. The review concludes with an outlook on future directions, aiming to advance the understanding of the cancer metabolic regulatory network and to inform the development of novel metabolism-based therapies.

## Metabolic reprogramming in cancer: an overview

### Hallmarks of cancer metabolism

Cancer exhibits multiple characteristic metabolic alterations. First, nutrient uptake and transport are markedly enhanced, including increased uptake of glucose [24, 25] and various amino acids [26, 27]. Notably, enhanced uptake does not necessarily require upregulation of all transporters; for example, L-type amino acid transporter 1 is downregulated in pancreatic cancer [24]. Moreover, under glucose-restricted conditions, AMP-activated protein kinase (AMPK) prioritizes cell survival by suppressing the cystine transporter solute carrier family 7 member 11 (SLC7A11), thereby limiting oxidative stress and ferroptosis [28]. Second, multiple metabolic pathways are highly active in cancer cells. Oncogenic signaling pathways such as hypoxia-inducible factor-1α (HIF-1α), myelocytomatosis viral oncogene homolog (c-Myc), mammalian target of rapamycin complex 1 (mTORC1), and sterol regulatory element–binding protein 1 (SREBP1) upregulate the enzymes that drive these metabolic programs. For example, cancer cells exhibit enhanced glycolysis and pentose phosphate pathway (PPP) activity [29, 30], and HIF-1α specifically promotes glycolytic and PPP flux [31]. Details of these oncogenic signals are discussed in subsequent sections. Third, cancer cells maintain robust antioxidant defenses. Unsaturated fatty acids and glutamine metabolism protect against ferroptosis [32], whereas nicotinamide adenine dinucleotide phosphate (NADPH) produced through the PPP and serine metabolism preserves redox homeostasis [30]. Fourth, cancer cells shape an immunosuppressive tumor microenvironment (TME) [33], inhibiting CD8⁺ T-cell activity [34]. Mechanistically, tumor cells induce the accumulation of cholesterol [35], histidine [36], and lactate [37, 38] in the microenvironment, collectively promoting immune suppression.

### Key metabolic pathways in carcinogenesis

After being transported into the cell, glucose enters glycolysis and is sequentially converted to pyruvate through multiple enzymatic reactions. The PPP branches from the glycolytic intermediate glucose-6-phosphate to generate ribose-5-phosphate and NADPH. Pyruvate derived from glycolysis can be converted to acetyl-CoA for mitochondrial oxidative phosphorylation (OXPHOS) or reduced to lactate. In cancer, glycolysis, the PPP, and lactate production are all upregulated and are essential for sustaining tumor cell survival [39, 40], whereas OXPHOS may be either enhanced or diminished depending on context [41].

De novo lipogenesis (DNL) uses acetyl-CoA and NADPH as substrates and converts them into fatty acids through multistep enzymatic reactions. Fatty acid β-oxidation (FAO) is the mitochondrial process in which fatty acids are degraded to acetyl-CoA. Acetyl-CoA also serves as the precursor for cholesterol biosynthesis. In cancer, both DNL [42] and cholesterol synthesis [43] are increased. FAO is generally elevated [44, 45], although reduced FAO has also been observed in certain settings [46].

Glutamine is converted to glutamate, which can be further metabolized to α-ketoglutarate to fuel the TCA cycle or be used for proline synthesis. Serine is synthesized from the glycolytic intermediate 3-phosphoglycerate (3PG), whereas asparagine is produced from the TCA cycle–derived intermediate oxaloacetate. Cancer cells display enhanced glutaminolysis to produce glutamate [47], as well as increased synthesis of multiple amino acids [48, 49].

## Glucose metabolic reprogramming in cancer

Glucose serves as the primary energy source for cells. Upon uptake, it is metabolized through glycolysis, the PPP, and OXPHOS. To meet elevated biosynthetic and energetic demands, cancer cells increase glucose uptake and enhance glycolytic activity even under aerobic conditions to exhibit the Warburg effect. In addition, cancer cells activate the PPP, while simultaneously suppressing gluconeogenesis and dampening OXPHOS. Moreover, recent studies have shown that lactate accumulation, primarily resulting from enhanced glycolysis in cancer, can promote tumor progression [50]. These metabolic shifts enable cancer cell survival, proliferation, invasion [51], and the acquisition of therapeutic resistance [52].

**Fig. 1:**
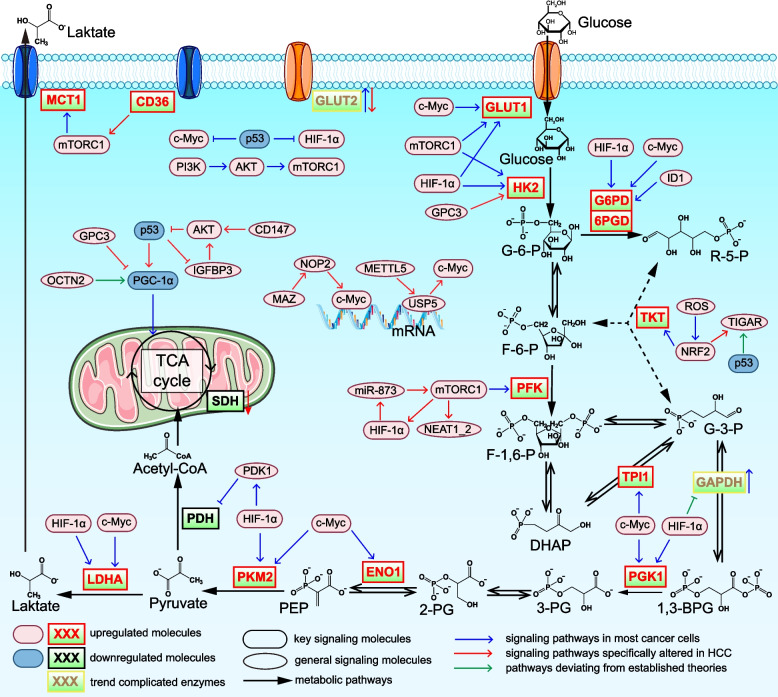
Signaling pathways regulating glucose metabolic reprogramming in cancer, including HCC-specific pathways. In tumors, HIF-1α is activated, oncogenes such as c-Myc are upregulated, tumor suppressors such as p53 are mutated or lost, and signaling molecules such as mTORC1 become hyperactivated. These alterations, along with additional HCC-specific molecular pathways, modulate the levels and activity of key enzymes within glucose metabolic pathways. As a result, glucose uptake is elevated, glycolytic flux is enhanced, mitochondrial activity is suppressed, the PPP is enhanced, and gluconeogenesis is inhibited. Collectively, these metabolic changes supply abundant energy and biosynthetic precursors through glycolysis and the PPP, while PPP-derived NADPH supports redox homeostasis, thereby promoting cancer cell survival, proliferation, invasion, and metastasis. Abbreviations: 1,3-BPG, 1,3-Bisphosphoglycerate; 2-PG, 2-phosphoglycerate; 3-PG, 3-phosphoglycerate; 6PGD, 6-phosphogluconate dehydrogenase; AKT, protein kinase B; c-Myc, myelocytomatosis viral oncogene homolog; DHAP, dihydroxyacetone phosphate; ENO1, enolase 1; F-1,6-P, fructose-1,6-bisphosphate; F-6-P, fructose 6-phosphate; G-3-P, glyceraldehyde 3-phosphate; G-6-P, glucose 6-phosphate; G6PD, glucose-6-phosphate dehydrogenase; GAPDH, glyceraldehyde-3-phosphate dehydrogenase; GLUT, glucose transporter; GPC3, glypican-3; HIF-1α, hypoxia-inducible factor-1α; HK-2, hexokinase 2; ID1, inhibitor of differentiation 1; IGFBP3, insulin-like growth factor binding protein-3; LDHA, lactate dehydrogenase A; MCT1, monocarboxylate transporter 1; METTL5, methyltransferase-like 5; mTORC1, mechanistic target of rapamycin complex 1; NEAT1_2, nuclear enriched abundant transcript 1 isoform 2; NOP2, nucleolar protein 2; NRF2, nuclear factor Erythroid 2-Like 2; OCTN2, organic cation/carnitine transporter 2; PDH, pyruvate dehydrogenase; PDK1, pyruvate dehydrogenase kinase 1; PEP, phosphoenolpyruvate; PGC-1α, proliferator-activated receptor gamma coactivator 1 α; PFK1, phosphofructokinase 1; PGK1, phosphoglycerate kinase 1; PI3K, phosphatidylinositol-3-kinase; PKM2, pyruvate kinase M2; R-5-P, ribose-5-phosphate; ROS, reactive oxygen species; TIGAR, TP53-induced glycolysis and apoptosis regulator; TKT, transketolase; TPI1, triosephosphate isomerase 1; USP5, ubiquitin-specific peptidase 5

Metabolic reprogramming of glucose in cancer is primarily mediated by several key mechanisms (Fig. 1). In normal cells, HIF-1α, which is activated under hypoxic conditions, serves as a potent driver of glucose metabolic reprogramming, particularly within the hypoxic TME [53]. In cancer, HIF-1α can also be activated even under non-hypoxic conditions [54], leading to the upregulation of key glycolytic enzymes in tumor cells. These enzymes include pyruvate kinase M2 (PKM2) [55], glucose transporter 1 (GLUT1) [56], lactate dehydrogenase A (LDHA) [57], hexokinase 2 (HK2), pyruvate dehydrogenase kinase 1 (PDK1) [58], phosphoglycerate kinase 1 (PGK1) [59], and glucose-6-phosphate dehydrogenase (G6PD) [60]. c-Myc is a central oncogene family regulating cell proliferation, metabolism, and survival, functioning as a potent transcription factor that plays a key role in glucose metabolic reprogramming [61]. It upregulates several crucial glycolytic enzymes, including LDHA, GLUT1, enolase 1 (ENO1), triosephosphate isomerase 1 (TPI1), PGK1, PKM2, and G6PD [62–64]. In contrast, tumor protein p53 is an essential tumor suppressor [65]. Its loss or mutation diminishes its inhibitory effects on glycolysis by weakening the suppression of c-Myc and HIF-1α [66]. As a result, glycolytic activity is enhanced [67] and mitochondrial function is impaired [68]. Additionally, the phosphatidylinositol-3-kinase (PI3K)/protein kinase B (AKT)/mammalian target of rapamycin (mTOR) pathway is a core oncogenic signaling axis frequently activated in tumors. mTORC1 promotes glucose metabolic reprogramming by inducing the levels of GLUT, monocarboxylate transporter 1 (MCT1) [69], HK2 [70], 6-phosphofructo-2-kinase/fructose-2,6-bisphosphatase 3, and phosphofructokinase 1 (PFK1) [71].

Interestingly, one study reported a paradoxical phenomenon in which the key glycolytic enzyme glyceraldehyde-3-phosphate dehydrogenase (GAPDH) is inhibited by HIF-1α [72]. However, it should be noted that this phenomenon has thus far been documented only in a limited number of cell types, such as renal cell carcinomas. Moreover, the pyruvate-deficient microenvironment implicated in this effect may not be universally present across human tumors.

### Glucose uptake and transporters

Cancer cells exhibit a high demand for glucose, reflected by increased glucose uptake and upregulation of glucose transporters, particularly GLUT1 [73]. GLUT1 abundance can be used to assess tumor grade [74] and may predict metastasis and mortality in cancer patients [75]. In HCC, both GLUT1 and GLUT3 are markedly upregulated [76]. Notably, GLUT2 is upregulated in various cancers [77, 78]. However, multiple studies have demonstrated that tumor cells from HCC patients exhibit reduced GLUT2 abundance [79, 80], and low GLUT2 levels have been shown to increase stemness [81]. The substantial experimental evidence for GLUT2 downregulation in HCC indicates its potential value as a distinctive diagnostic marker for this malignancy.

Beyond the GLUT family, sodium-glucose cotransporters (SGLTs) are also found to be upregulated in multiple human tumor types [82, 83]. Several SGLT inhibitors originally developed for diabetes management have advanced into preclinical and clinical evaluation for cancer therapy [84]. In HCC, treatment with the SGLT2 inhibitor canagliflozin not only suppresses glycolytic flux but also induces mitotic arrest and apoptosis [85]. Notably, SGLT2 inhibitors also reduce the risk of non-malignant NAFLD progressing to HCC [86]. These observations indicate that SGLT inhibition may emerge as an important strategy for both HCC treatment and prevention.

### Glycolysis and the warburg effect

Glycolysis is a core metabolic pathway in which glucose is converted into pyruvate in the cytoplasm, generating a small amount of ATP. Its key enzymes include HK2, PFK1, PKM2, and PGK1. The Warburg effect, also known as aerobic glycolysis, is one of the fundamental metabolic features of cancer. Even in the presence of sufficient oxygen, cancer cells preferentially convert glucose into lactate through glycolysis, characterized by enhanced glycolytic flux, increased lactate production and accumulation, and suppressed mitochondrial OXPHOS [87, 88]. Although seemingly inefficient, this metabolic shift provides cancer cells with essential intermediates. These intermediates are diverted into biosynthetic pathways, including the PPP for ribose generation [89] and the serine–glycine synthesis pathway for non-essential amino acid production [90]. The resulting hypoxic and acidified TME further promotes tumor invasion, metastasis, and immune suppression [91, 92].

As noted above, key regulators such as c-Myc, AKT/mTOR, and p53 contribute to the establishment of the Warburg effect in cancer (Fig. 1). Recent studies have uncovered additional mechanisms of c-Myc activation in HCC. Methyltransferase-like protein 5 enhances the translation of ubiquitin-specific peptidase 5. Ubiquitin-specific peptidase 5 then inhibits K48-linked polyubiquitination of c-Myc, thereby increasing its protein stability and activating downstream glycolytic genes [63]. In addition, MYC-associated zinc finger protein functions as a transcription factor that upregulates the S-adenosyl-L-methionine–dependent methyltransferase nucleolar protein 2. Nucleolar protein 2 stabilizes c-Myc mRNA and promotes its translation, further driving glycolytic reprogramming in HCC [93]. The lncRNA nuclear enriched abundant transcript 1 (NEAT1) appears to be a critical downstream target of mTORC1 in HCC. mTORC1 suppresses NEAT1_2, thereby enhancing mRNA splicing and increasing the levels of key glycolytic enzymes [94]. The fatty acid receptor CD36 further amplifies aerobic glycolysis through the AKT/mTOR signaling pathway [95]. Additionally, in HCC, miR-595 promotes glycolysis and lipid droplet accumulation by activating HIF-1α [96]. Intriguingly, one study reported an unusual phenomenon in HCC in which p53 levels are elevated. This apparent paradox is explained by the finding that the Scribble–p53 complex activates oncogenic rather than tumor-suppressive programs [97]. The evidence supporting this mechanism is robust, and the discovery represents a novel insight into HCC biology.

These oncogenic regulators do not function independently (Fig. 1). AKT/mTOR acts upstream of HIF-1α [98], while evidence also shows that, in HCC, HIF-1α upregulates miR-873, which in turn activates AKT/mTOR-mediated Warburg metabolism [99]. In HCC, p53 also exhibits crosstalk with the AKT/mTOR pathway. The degradation of p53 leads to the activation of insulin-like growth factor binding protein-3 (IGFBP3)-dependent AKT/mTOR signaling [100]. Conversely, CD147 inhibits p53 via PI3K/AKT pathway activation. Reduced p53 levels then induce GLUT1 upregulation and phosphofructokinase liver type activation to enhance glycolysis. Simultaneously, low levels of p53 downregulate peroxisome proliferator-activated receptor gamma coactivator 1α (PGC-1α), mitochondrial transcription factor A, and p53-induced ribonucleotide reductase M2B subunit to impair mitochondrial function [101].

Besides the aforementioned molecules, other molecular regulators also influence the Warburg effect in HCC. The transmembrane heparan sulfate proteoglycan glypican-3 (GPC3) is upregulated on the surface of HCC cells. GPC3 enhances glycolysis by upregulating key glycolytic genes, including LDHA, HK2, and GLUT1, and suppresses mitochondrial respiration by downregulating PGC-1α, thereby driving aerobic glycolysis [102]. In addition, reduced levels of the pyruvate dehydrogenase E1α subunit (PDHA1) have been observed in HCC [103].

### Pentose phosphate pathway

The PPP, a critical branch of glucose metabolism, is highly active in cancer. The PPP generates NADPH via 6-phosphogluconate dehydrogenase (6PGD) to scavenge reactive oxygen species (ROS) [104] and to maintain lipid synthesis [105]. The non-oxidative phase provides nucleotide precursors through transketolase (TKT) to fuel proliferation [106]. Inhibition of 6PGD activates acetyl-CoA carboxylase 1 (ACC1) and AMPK, leading to a reduction in the NADPH/NADP + ratio, which triggers oxidative stress [107]. In addition, research found that inhibitor of differentiation 1 promotes G6PD transcription and activates the PPP via the wingless-related integration site/β-catenin/c-Myc axis, thereby facilitating HCC proliferation [108]. TKT abundance is regulated by the NRF2/KEAP1/BACH1 signaling axis. Accumulation of ROS in tumor cells inactivates kelch-like ECH-associated protein 1 (KEAP1), leading to reduced degradation of nuclear factor erythroid 2–related factor 2 (NRF2). The stabilized NRF2 competes with BTB and CNC homolog 1 (BACH1) for binding to antioxidant response elements (AREs) within the TKT promoter, thereby enhancing TKT transcription [104]. Concurrently, NRF2 binds to AREs within the TP53-induced glycolysis and apoptosis regulator (TIGAR) promoter, upregulating TIGAR and further activating the PPP [109]. Although TIGAR suppresses glycolysis in HCC as a downstream target of p53, its elevated levels paradoxically support tumor survival through stimulating the PPP, which scavenges ROS and protects cells from oxidative stress [110] (Fig. 1).

### Mitochondrial respiration

Traditional views based on the Warburg effect suggest that cancer cells rely mainly on glycolysis while suppressing mitochondrial OXPHOS. However, recent evidence indicates that some tumors undergo a metabolic shift toward increased OXPHOS during progression [111, 112]. In HCC, OXPHOS is often enhanced, and its inhibition compromises tumor cell survival [113, 114], likely because OXPHOS provides ample ATP and supplies precursors for glutamate and lipid synthesis. For instance, organic cation/carnitine transporter 2 (OCTN2) promotes stem-like traits in HCC by elevating OXPHOS through PGC-1α activation [115]. Additionally, activated signal transducer and activator of transcription 3 increases c-Myc levels in HCC, thereby promoting mitochondrial OXPHOS and biogenesis [113]. The key TCA cycle enzyme, succinate dehydrogenase (SDH), is downregulated in HCC. These low SDH levels appear contradictory to the enhanced OXPHOS observed in HCC, yet multiple reports confirm the co-existence of both phenomena. Crucially, studies indicate that SDH drives HCC through the activation of yes-associated protein (YAP) and transcriptional coactivator with PDZ-binding motif (TAZ) [116]. Moreover, downregulation of SDH leads to ROS accumulation and activation of the nuclear factor-κB signaling pathway [117] and enhances cellular proliferation and motility [118]. This suggests that the pro-carcinogenic role of low SDH may be independent of its effect on OXPHOS and instead directly mediated by downstream oncogenic factors.

### Other alterations in glucose metabolism

In several cancers, including HCC, gluconeogenesis is suppressed [119, 120]. The gluconeogenic enzymes phosphoenolpyruvate carboxykinase 1 (PCK1) and PCK2 are downregulated in HCC, a pattern associated with poor patient prognosis [121]. In HCC cells, reduced PCK1 levels also function as a signaling cue, whereby PCK1 deficiency increases the O-GlcNAcylation of checkpoint kinase 2, thereby promoting HCC growth [122].

Key enzymes and transporters of lactate metabolism are altered in HCC. LDH upregulation drives lactate accumulation and accelerates glycolysis [123], while MCT upregulation mediates acid efflux [124], shaping the TME. One study demonstrated that lactate accumulation can also lead to lysine lactylation, thereby mediating metabolic changes [125]. Furthermore, in HCC cells, high MCT1 levels promote ATP production, resulting in AMPK deactivation and subsequent upregulation of SREBP1 [126].

## Lipid metabolic reprogramming in cancer

**Fig. 2:**
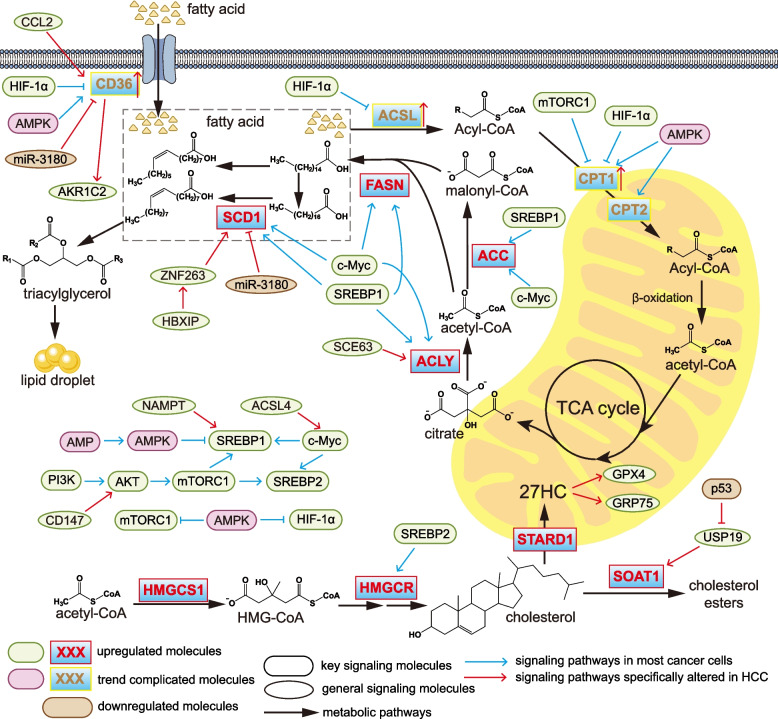
Signaling pathways regulating lipid metabolic reprogramming in cancer, including HCC-specific pathways. In cancer cells, the PI3K/AKT/mTORC1 pathway is activated, leading to the upregulation of SREBP1. Together with aberrantly activated oncogenic c-Myc, SREBP1 enhances de novo lipogenesis by upregulating fatty acid–synthesizing enzymes. Under low-energy conditions, activated AMPK promotes fatty acid β-oxidation in cancer cells. In addition, cancer cells exhibit increased lipid uptake as well as elevated cholesterol uptake and synthesis. These lipid metabolic alterations supply membrane components required for rapid cell division, confer resistance to ferroptosis, and may contribute to the establishment of an immunosuppressive microenvironment. Abbreviations: ACC, acetyl-CoA carboxylase; ACLY, ATP-citrate lyase; ACSL, acyl-CoA synthetase long-chain; AKR1C2, Aldo–Keto Reductase Family 1 Member C2; AMPK, AMP-activated protein kinase; CCL2, C–C motif chemokine ligand 2; CD36, cluster of differentiation 36; CPT, carnitine palmitoyltransferase; c-Myc, myelocytomatosis viral oncogene homolog; FASN, fatty acid synthase; GRP75, glucose-regulated protein 75; GPX4, glutathione peroxidase 4; HBXIP, hepatitis B X-interacting protein; HIF-1α, hypoxia-inducible factor-1α; HMG, 3-hydroxy-3-methylglutaryl; HMGCR, 3-hydroxy-3-methylglutaryl-CoA reductase; HMGCS1, 3-hydroxy-3-methylglutaryl-CoA synthase 1; mTORC1, mammalian target of rapamycin complex 1; NAMPT, nicotinamide phosphoribosyltransferase; SCD1, stearoyl-CoA desaturase 1; SEC63, protein transport protein Sec63; SOAT1, sterol O-acyltransferase 1; SREBP, sterol regulatory element–binding protein; STARD1, steroidogenic acute regulatory protein D1; USP19, ubiquitin-specific peptidase 19; ZNF263, zinc finger protein 263

HCC cells exhibit enhanced lipid uptake, increased DNL, and elevated FAO to maintain HCC survival. These metabolic alterations collectively sustain energy production, supply biosynthetic precursors, and promote cancer cell survival and proliferation by activating lipid-mediated signaling pathways (Fig. 2).

The PI3K/AKT/mTOR pathway is frequently activated in cancer. Activated mTORC1 drives lipogenesis primarily by upregulating the transcription factor SREBP1 [127], and concurrently suppresses FAO by inhibiting carnitine palmitoyltransferase 1 (CPT1) [128]. SREBP1 binds to the promoters of lipogenic enzyme genes and upregulates their expression, including that of fatty acid synthase (FASN), ATP-citrate lyase (ACLY), ACC1, and stearoyl-CoA desaturase 1 (SCD1) [129–131]. Beyond SREBP1, mTORC1 also regulates lipogenesis by activating SREBP2, thereby upregulating 3-hydroxy-3-methylglutaryl-CoA reductase (HMGCR), a key enzyme in the cholesterol synthesis pathway [132, 133]. The oncogene c-Myc exerts effects similar to those of SREBP1, inducing the upregulation of lipogenic genes such as FASN, ACC1, and ACLY [134–136]. Furthermore, c-Myc can activate the transcription of SREBP1/2 [134] and enhance upstream glucose metabolism to provide energy for lipid biosynthesis. However, numerous studies indicate that lipid metabolism in cancer is dynamic: under energy-stressed conditions, AMPK is activated by a high AMP/ATP ratio, shifting metabolism toward catabolism while inhibiting anabolic processes [137]. Specifically, AMPK activation under hypoxia or glucose deprivation [138] leads to the upregulation of key FAO enzymes CPT1, CPT2, and CD36 [139], and suppresses lipogenesis via SREBP1 inhibition [140].

### Lipid uptake and trafficking

In cancer, lipid uptake is increased, primarily reflected by the elevated CD36 levels [141]. CD36, a class B scavenger receptor [142], mediates the uptake of free fatty acids as well as oxidized low-density lipoproteins [143, 144]. Studies have shown that CD36 is also upregulated in HCC, where it upregulates aldo–keto reductase family 1 member C2 (AKR1C2). Together, CD36 and AKR1C2 enhance fatty acid uptake and drive HCC proliferation and metastasis [145]. In HCC tissues, eva-1 homolog A (EVA1A) levels are reduced [146], and EVA1A deficiency upregulates CD36, promoting fatty acid uptake [147]. In HCC tissues, miR-3180 is downregulated, resulting in reduced repression of CD36 and SCD1, thereby facilitating HCC cell proliferation, migration, and invasion [148]. Additionally, HCC-derived periostin induces C–C motif chemokine ligand 2 (CCL2) secretion in endothelial progenitor cells. CCL2 subsequently enhances CD36 production in HCC cells, further supporting tumor progression [149] (Fig. 2).

Notably, CD36 is also implicated in the pathogenesis of HCC. High CD36 levels in NASH [150] upregulate Nogo-B, leading to lysophosphatidic acid-stimulated yes-associated protein oncogenic activity, thereby increasing the risk of NAFLD transformation into HCC [144].

Furthermore, multiple studies have demonstrated an intriguing phenomenon: CD36 is not only functional within HCC cells, but its upregulation in surrounding cells also promotes HCC progression. For instance, CD36 levels are elevated in cancer-associated fibroblasts. These CD36-upregulating cancer-associated fibroblasts secrete the macrophage migration inhibitory factor [151] and inhibit cytotoxic T lymphocyte activity by inducing programmed cell death protein 1 production [152]. Paradoxically, one study demonstrated that the fatty acid uptake of human HCC samples was lower than that of adjacent normal tissues [153]. This study has certain limitations: it had a small sample size, and all samples were exclusively early-stage HCC samples. Furthermore, it is inappropriate to use peritumoral tissues as normal controls, and these factors may have collectively contributed to the formulation of the aforementioned result.

### De Novo Lipogenesis

In cancer, DNL is enhanced [154]. The primary product of DNL is palmitic acid [155], which can be further elongated to stearic acid. In cancer cells, SCD1 is upregulated and converts palmitic and stearic acids into palmitoleic and oleic acids. These fatty acids contribute to phospholipid synthesis, providing membrane components required for rapid cell division and promoting cellular invasion [156]. The resulting unsaturated fatty acids also protect cancer cells from ferroptosis [157, 158]. Similarly, DNL is elevated in HCC, where it facilitates immune evasion and is associated with poor prognosis [159].

In HCC, SREBP1 is upregulated and promotes the transcription of key enzymes involved in DNL [160], thereby driving tumor progression. Several studies have identified upstream pathways responsible for SREBP1 activation in HCC (Fig. 2). Acyl-CoA synthetase long-chain family member 4 (ACSL4) enhances SREBP1 production via c-Myc upregulation [161]. CD147 activates SREBP1c through the AKT/mTOR signaling pathway [162]. In addition, nicotinamide phosphoribosyltransferase is upregulated in HBV-positive HCC cells and promotes SREBP1-mediated lipogenesis [163].

Lipogenic enzymes in HCC are also regulated by several additional factors. In HCC, miR-3180 is downregulated, resulting in diminished suppression of SCD1 [148]. Moreover, hepatitis B X-interacting protein enhances SCD1 transcription through co-activating zinc finger protein 263 [164]. The protein transport protein Sec63 further drives lipogenesis by stabilizing ACLY, thereby increasing acetyl-CoA production [165].

In cancer, increased lipid uptake and enhanced DNL lead to the accumulation of lipid droplets, which contribute to drug resistance and the formation of an immunosuppressive microenvironment [166, 167]. In HCC, AKR1C3 promotes the storage of triacylglycerol in lipid droplets, preventing cytosolic fatty acid accumulation and thereby inducing sorafenib resistance [168]. Conversely, reducing lipid droplet deposition through pharmacologic intervention may inhibit HCC progression [169].

### Fatty acid oxidation

In cancer, the intensity of FAO is highly dynamic, and both the upregulation and suppression of FAO have been observed to confer advantages to tumor cells. HIF-1α suppresses the mRNA expression of key FAO enzymes, including CPT1 and ACSL, as well as the fatty acid transporter CD36 [170, 171]. Similarly, activated mTOR downregulates CPT1A [172]. In contrast, under conditions of low cellular energy, an increased AMP/ATP ratio activates AMPK, which subsequently stimulates FAO through the activation of CPT1 [173]. Moreover, AMPK suppresses both mTOR and HIF-1α activity [174], thereby counteracting their inhibitory effects on FAO (Fig. 2).

Specific metabolic alterations in HCC involve the upregulation of FAO. This can be driven by mechanisms such as aberrantly activated β-catenin, which promotes FAO through PPARα upregulation [175], and elevated OCTN2, which enhances FAO via the PGC-1α pathway [115]. In addition, stromal interaction molecule 1 (STIM1) downregulation increases CPT1 [176]. ACSL enzymes are also involved in FAO, as fatty acids must be linked to CoA via ACSL before β-oxidation. ACSL1 and ACSL4 are observed to be upregulated in HCC [177, 178]. These alterations collectively facilitate HCC growth and metastasis. Conversely, distinct from the typical FAO enhancement seen in HCC, E2F transcription factor 2 is upregulated in NAFLD-related HCC, which attenuates FAO by repressing CPT2 [179]. This CPT2 downregulation causes acylcarnitine accumulation, inducing the development of HCC [180].

Notably, FAO alterations in immune cells within the HCC microenvironment can suppress antitumor immunity. During HCC development, elevated fatty acid-binding protein 5 (FABP5) in monocytes reduces the rate of β-oxidation, leading to lipid droplet accumulation and immunosuppression [181]. In contrast, in tumor-associated macrophages, receptor-interacting serine/threonine kinase 3 (RIPK3) deficiency enhances FAO through PPAR activation, thereby suppressing their immune activity [182].

### Cholesterol and membrane lipid metabolism

Cholesterol is an essential component of the cellular membrane, and low-density lipoprotein serves as its primary carrier in the bloodstream. In many cancers, cholesterol biosynthesis is elevated [183], accompanied by increased levels of the low-density lipoprotein receptor and the cholesterol-regulating transcription factor SREBP2 [184]. SREBP2 further enhances cholesterol production by upregulating key enzymes in the biosynthetic pathway, including 3-Hydroxy-3-methylglutaryl-CoA synthase 1 (HMGCS1) and HMGCR [133, 185].

Alterations in cholesterol metabolism are also evident in HCC. Upregulation of PPARδ enhances cholesterol synthesis [186], and elevated cholesterol production in HCC is associated with poorer prognosis [187]. Notably, cholesterol can also act as a signaling molecule. It suppresses receptor tyrosine kinase degradation, which amplifies oncogenic signaling to promote HCC progression [188]. Cholesterol metabolism is closely linked to therapeutic resistance. SREBP2 has been shown to mediate tyrosine kinase inhibitor resistance in HCC [189], and similarly, high levels of HMGCR counteract the tumor-suppressive effects induced by FASN depletion [183]. High cholesterol levels additionally contribute to an immunosuppressive microenvironment. ATP-binding cassette subfamily A member 1 is upregulated in monocytes and macrophages in the TME, promoting cholesterol efflux and suppressing their immune activity [190]. A cholesterol-rich environment also inhibits natural killer T cell function [35]. However, one study reported that elevated circulating low-density lipoprotein cholesterol reduces the risk of developing HCC [191]. These findings suggest that LDL-C may exert context-dependent effects—higher levels in the circulation appear to suppress HCC initiation, whereas elevated levels within the TME may instead promote tumor progression. Thus, the two observations are not mutually exclusive.

Several additional alterations in cholesterol metabolism have been identified in HCC (Fig. 2). Excess cytosolic cholesterol is esterified and stored in lipid droplets, a process that is enhanced in HCC. Loss of p53 diminishes its repression of ubiquitin-specific peptidase 19, leading to activation of the cholesterol acyltransferase sterol O-acyltransferase 1 (SOAT1) [192]. Steroidogenic acute regulatory protein D1 (STARD1), the rate-limiting transporter that delivers cholesterol to mitochondria for bile acid synthesis, is also upregulated in HCC [193]. 27-hydroxycholesterol (27HC) is an intermediate in the conversion of cholesterol to bile acids. In HCC, elevated transmembrane protein 147 increases 27HC, which in turn upregulates glutathione peroxidase 4 to suppress ferroptosis [194]. Moreover, 27HC activates glucose-regulated protein 75 and its downstream effectors HIF-1α and c-Myc, thereby modulating glucose metabolism [195].

Alterations in membrane lipid metabolism also occur in cancer. Enhanced DNL and SCD1 upregulation in cancer cells [148, 154] promote fatty acid synthesis, providing precursors for membrane lipids. SCD1 synthesizes unsaturated fatty acids; the resulting monounsaturated fatty acids suppress ferroptosis [196]. Meanwhile, the generated oleic acid increases the proportion of unsaturated fatty acids in membrane phospholipids, enhancing plasma membrane fluidity and promoting invasive cancer cell metastasis [156]. Upregulated ACSL4 in tumors drives the preferential incorporation of polyunsaturated fatty acids into phospholipids, promoting cancer cell metastasis [197]. Furthermore, a high phosphatidylcholine/phosphatidylethanolamine ratio in cancer cell phospholipids favors cell proliferation [198].

Sphingolipid metabolism is also altered in cancer, characterized by enhanced ceramide hydrolysis [199] and increased sphingosine-1-phosphate synthesis, which activates sphingosine kinase to exert pro-oncogenic effects [200]. Notably, ceramide possesses pro-apoptotic properties and plays a dual role in HCC development. The accumulation of ceramide in NASH exacerbates hepatic steatosis, inflammation, and fibrosis [201]. In contrast, high hypoxia-inducible lipid droplet associated protein levels in established HCC reduce ceramide production, helping HCC cells evade apoptosis [202] and promoting tumor progression.

## Amino acid metabolic reprogramming in cancer

**Fig. 3:**
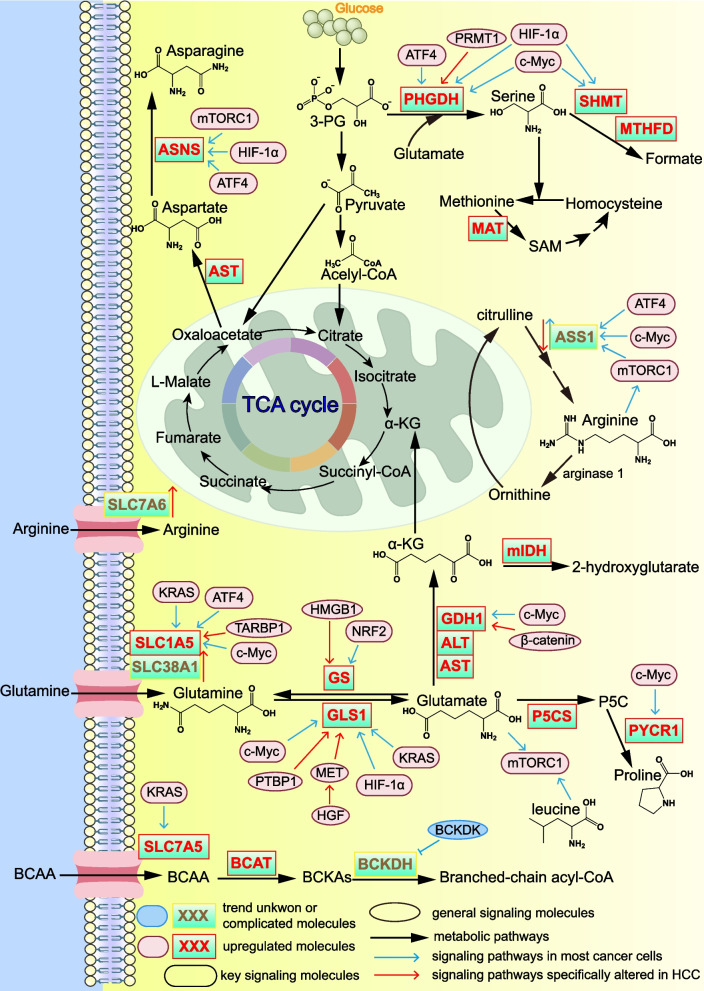
Signaling pathways regulating amino acid metabolic reprogramming in cancer, including HCC-specific pathways. Uptake, synthesis, and utilization of multiple amino acids—including glutamine, asparagine, serine, arginine, proline, and branched-chain amino acids—are markedly increased in cancer cells. Amino acid metabolic reprogramming is driven by several signaling regulators, including c-Myc, mTORC1, HIF-1α, ATF4, NRF2, and KRAS. These alterations not only supply substrates for protein synthesis but also provide precursors for nucleotide production and DNA methylation, maintain redox homeostasis, and function as signaling mediators that promote tumor progression. Collectively, these metabolic changes support the biosynthetic demands of rapidly proliferating cancer cells and contribute to therapeutic resistance. Abbreviations: 3-PG, 3-phosphoglycerate; ALT, alanine aminotransferase; α-KG, α-ketoglutarate; ASNS, asparagine synthetase; ASS1, argininosuccinate synthase-1; AST, aspartate aminotransferase; ATF4, activating transcription factor 4; BCAA, branched-chain amino acids; BCAT, branched-chain amino acid transaminase; BCKAs, branched-chain α-keto acids; BCKDH, branched-chain α-keto acid dehydrogenase; BCKDK, branched-chain α-keto acid dehydrogenase kinase; c-Myc, myelocytomatosis viral oncogene homolog; GDH1, glutamate dehydrogenase 1; GLS, glutaminase; GS, glutamine synthetase; HGF, hepatocyte growth factor; HIF-1α, hypoxia-inducible factor-1α; HMGB1, high-mobility group box 1; KRAS, kirsten rat sarcoma viral oncogene homolog; MAT, methionine adenosyltransferase; MET, mesenchymal-epithelial transition factor; mIDH, mutant isocitrate dehydrogenase; MTHFD, methylenetetrahydrofolate dehydrogenase; mTORC1, mammalian target of rapamycin complex 1; NRF2, nuclear factor erythroid 2–related factor 2; P5C, pyrroline-5-carboxylate; P5CS, P5C synthase; PHGDH, phosphoglycerate dehydrogenase; PRMT1, protein arginine methyltransferase 1; PTBP1, polypyrimidine tract-binding protein 1; PYCR, pyrroline-5-carboxylate reductase; SAM, S-adenosylmethionine; SHMT, serine hydroxymethyltransferase; SLC, solute carrier; TARBP1, TAR RNA binding protein 1

In cancer cells, the uptake and synthesis of multiple amino acids—including glutamine, asparagine, serine, arginine, proline, and branched-chain amino acids (BCAAs)—are markedly increased (Fig. 3). These amino acids not only supply substrates for protein synthesis but also provide essential precursors for nucleotide and DNA synthesis. They also support redox homeostasis and act as signaling molecules to promote tumor progression. Moreover, glutamine serves as an important energy source for tumor cells.

Multiple signaling pathways regulate amino acid–metabolizing enzymes, thereby driving amino acid metabolic reprogramming in cancer cells (Fig. 3). c-Myc upregulates SLC1A5, glutaminase 1 (GLS1), glutamate dehydrogenase 1 (GDH1), phosphoglycerate dehydrogenase (PHGDH), serine hydroxymethyltransferase (SHMT), argininosuccinate synthase-1 (ASS1), and pyrroline-5-carboxylate reductase 1 (PYCR1) [203–208]. Moreover, mTORC1 upregulates asparagine synthetase (ASNS) and ASS1 [209–211]. HIF-1α increases the levels of GLS1, PHGDH, SHMT2, and ASNS [212–215]. Activating transcription factor 4 (ATF4), a stress-responsive transcription factor, induces the levels of ASNS, ASS1, PHGDH, and SLC1A5 while sustaining mTORC1 activation [216]. NRF2 enhances the levels of glutamine synthetase (GS) and glutamate transporter 1 [217]. Kirsten rat sarcoma viral oncogene homolog (KRAS) transcriptionally activates SLC1A5, SLC7A5, and GLS [217]. In particular, amino acids are capable of functioning as signaling molecules. The key oncogenic regulator mTORC1 can be activated by amino acids, with elevated levels of leucine, arginine, methionine, and glutamine serving as potent activators of mTORC1 [218–220].

### Glutamine metabolism

In cancer, glutamine uptake is markedly increased. Abundant glutamine is converted to glutamate by GLS to fuel bioenergetic needs, support downstream biosynthetic pathways, and activate the oncogenic regulator mTORC1 [221]. The solute carrier (SLC) family mediates the transmembrane transport of metabolites and includes transporters for multiple amino acids [222]. Among these, the glutamine transporters SLC38A1 and SLC1A5 are upregulated in HCC, resulting in enhanced glutamine uptake [223]. Moreover, oncogenic Kruppel-like factor 7 and the metabolic regulator TAR RNA binding protein 1 have been reported to increase SLC1A5 levels in HCC [224, 225].

In cancer, including HCC, GLS1 is markedly upregulated and hyperactivated, and this elevation is strongly associated with poor prognosis [226, 227]. In HCC, GLS1 can be induced by hepatocyte growth factor activated mesenchymal-epithelial transition factor (MET) signaling or by polypyrimidine tract-binding protein 1, both of which promote chemoresistance [228, 229]. Moreover, extracellular signal-regulated kinase and SET and MYND domain-containing protein 2 upregulate c-Myc, thereby driving GLS1 transcription and enhancing glutamine utilization [230–232]. The resulting glutamate supports glutathione (GSH) synthesis to detoxify ROS and suppress ferroptosis. High levels of the GSH-biosynthetic enzymes glutamate-cysteine ligase catalytic subunit and glutathione synthetase are associated with poor prognosis in cancer cells [233, 234]. Conversely, GLS1 downregulation reduces GSH production, leading to ROS accumulation and apoptosis [230]. In contrast to GLS1, GLS2 exerts tumor-suppressive activity by promoting ferroptosis; accordingly, its abundance is decreased in cancer [235].

Glutamate can be converted to α-ketoglutarate (α-KG) by alanine aminotransferase (ALT) 1/2, aspartate aminotransferase 1/2 (AST1/2), or GDH1, thereby fueling the TCA cycle and ATP production. α-KG may also be reduced by mutant isocitrate dehydrogenase 1 (mIDH1) to generate 2-hydroxyglutarate, a metabolite implicated in cancer progression [236, 237]. Glutaminolysis supports cancer cell survival under low-glucose conditions [238], and concurrent inhibition of both GLUT1 and GLS1 induces ROS accumulation and apoptosis [239]. ALT1/2 and AST1/2 are highly synthesized in multiple cancers [240]. Their abundant presence in liver and HCC underlies their routine clinical use as serum markers for hepatic injury; they are also drivers of NAFLD progression [241]. In HCC, the β-catenin/lymphoid enhancer-binding factor 1 complex activates transcription of GDH1, thereby enhancing the generation of glutamate-derived aspartate and proline [242]. However, another study reported that GDH1 is downregulated in HCC [243]. This apparent discrepancy can be reconciled by evidence showing that AST predominantly mediates glutaminolysis when glucose is sufficient. Under glucose-deprived conditions, GDH1 becomes the primary enzyme driving glutamine catabolism in HCC to sustain energy production and cell survival [244].

GS, which catalyzes the synthesis of glutamine from glutamate and ammonia, is upregulated in HCC. For example, high-mobility group box 1, which is elevated in HCC, activates GS production [245]. Clinically, high GS levels serve as a diagnostic biomarker for HCC [246] and predict poor survival outcomes in patients with HCC [247]. Consistently, GS-positive HCC patients exhibit significantly worse prognosis compared with GS-negative patients [248]. Moreover, GS induces the development of NASH and is concurrently a risk factor for HCC occurrence [249].

### Asparagine and serine

Oxaloacetate generated from the TCA cycle or via pyruvate carboxylation is converted to aspartate through AST-mediated transamination with glutamate. AST levels are also increased in cancer, particularly in HCC [250]. The resulting aspartate serves as an essential precursor for purine and pyrimidine biosynthesis, thereby supporting nucleotide production required for tumor cell proliferation [251, 252]. Asparagine metabolism is elevated in cancers, including HCC, and is associated with poor patient prognosis [253]. Asparagine biosynthesis is enhanced in cancer cells, and even under glutamine-deprived conditions, HCC cells maintain asparagine production, underscoring its metabolic necessity [254]. Asparagine depletion suppresses HCC progression [255], and therapeutic depletion of asparagine using asparaginase has been proposed as a potential anticancer strategy [256]. ASNS, which catalyzes the conversion of aspartate to asparagine [257], is upregulated in HCC tissues [258]. For example, elevated pituitary tumor-transforming gene 1 in HCC enhances ASNS transcription [259]. However, another study reported reduced ASNS mRNA abundance in the peripheral blood of HCC patients [260]. This apparent discrepancy may be attributed to methodological differences, as the latter study quantified circulating ASNS mRNA rather than ASNS abundance within tumor tissues.

Serine abundance is elevated in cancer cells [261], and serine plays essential metabolic and biosynthetic roles in tumor progression. Serine serves as a substrate for sphingolipid biosynthesis [262] and provides one-carbon units—via formate production and serine-derived S-adenosylmethionine (SAM)—that are critical for nucleotide synthesis [263]. Enhanced serine biosynthesis arises from the glycolytic intermediate 3PG, which is converted to serine in a glutamate-dependent reaction catalyzed by PHGDH. PHGDH is upregulated across multiple cancers [264], and its depletion suppresses tumor growth [265]. In HCC, protein arginine methyltransferase 1 enhances PHGDH enzymatic activity and thereby promotes tumor progression [261]. Serine is further metabolized by SHMT1/2 and methylenetetrahydrofolate dehydrogenase 1/2 (MTHFD1/2) to generate formate, which supplies one-carbon units for anabolic processes [266]. Both SHMT and MTHFD activities are elevated in cancer cells [266, 267]. Inhibitors targeting PHGDH and SHMT have entered preclinical drug development pipelines [268, 269].

### Arginine and proline metabolism

Arginine is generated in the urea cycle. Citrulline is converted to arginine through sequential catalysis by argininosuccinate synthase 1 (ASS1) and argininosuccinate lyase, and subsequently metabolized by arginase 1 to produce urea and ornithine. In some cancers, ASS1 abundance is elevated, and high ASS1 levels have been reported to confer resistance to ferroptosis [173, 270]. However, in many malignancies, including HCC, ASS1 is downregulated [271, 272], and restoring ASS1 levels may exert tumor-suppressive effects [273]. Although de novo arginine synthesis is diminished in HCC, increased arginine uptake and reduced arginine catabolism collectively lead to intracellular arginine accumulation [274]. For example, the arginine transporter SLC7A6 is upregulated in HCC [223], whereas argininosuccinate lyase is downregulated in breast cancer cells [275]. Beyond its role in protein synthesis, arginine also functions as a signaling metabolite capable of activating mTORC1 [219].

Glutamate is converted into proline through sequential catalysis by P5C synthase (P5CS) and PYCR. In HCC, the proline biosynthetic enzymes P5CS and PYCR1 are markedly upregulated [276, 277]. Proline-rich protein 11 also exerts pro-tumorigenic effects and promotes cancer progression [278]. Moreover, the downstream proline metabolite hydroxyproline accumulates in HCC. Hydroxyproline stabilizes HIF-1α and contributes to collagen synthesis, thereby facilitating tumor growth and metastasis [279, 280].

### Branched-chain amino acids and others

Cancer cells exhibit intracellular accumulation of BCAAs [281], which supports protein synthesis and exerts pro-tumorigenic effects. In HCC, inhibition of leucine catabolism by dihydrolipoamide S-acetyltransferase leads to leucine accumulation, which activates mTOR signaling to promote tumor progression [282, 283]. In contrast to the elevated intracellular BCAA levels, serum BCAA concentrations are decreased in patients with HCC, typically reflected by reduced BCAA/tyrosine ratios [284, 285]. This discrepancy likely reflects enhanced uptake and utilization of circulating BCAAs by tumor cells rather than a true contradiction. The BCAA transporter SLC7A5 is upregulated in multiple cancers, facilitating increased BCAA influx [218]. Branched-chain amino acid transaminase 1 (BCAT1) and BCAT2, which catalyze the conversion of BCAAs to branched-chain α-keto acids (BCKAs), are also elevated in cancer and support tumor growth [286]. In HCC, BCAT1 abundance positively correlates with AKT activation, thereby contributing to oncogenic signaling. The resulting BCKAs can be further metabolized by the branched-chain α-keto acid dehydrogenase (BCKDH) complex to fuel energy production or biosynthesis. Notably, BCKDH abundance varies with cellular context: after chemotherapy, high branched-chain α-keto acid dehydrogenase kinase (BCKDK) levels suppress BCKDH activity and promote drug resistance [287]. However, following radiotherapy, upregulation of BCKDH enhances BCAA degradation and contributes to radioresistance [288].

Serine provides one-carbon units that enable the conversion of homocysteine to methionine, which is subsequently transformed into SAM by methionine adenosyltransferases (MATs). SAM functions as a universal methyl donor [289] and also stabilizes glutathione peroxidase 4 to protect against ferroptosis [290]. Serine availability is therefore crucial for SAM biosynthesis. In cancer cells, elevated SHMT2 increases SAM production [291], and in HCC, high levels of PCK1 enhance serine synthesis to further promote SAM generation [292]. MAT enzymes play essential roles in cancer biology. MAT1A is upregulated in tumor cells [293], and inhibition of MAT2A suppresses cancer cell proliferation and growth, highlighting its potential as a therapeutic target [294, 295].

## Crosstalk among metabolic pathways in cancer

### Glucose–lipid metabolic interplay

Intermediates of glucose and lipid metabolism are coupled with oncogenic signaling pathways. Acetyl-CoA and NADPH generated from glycolysis directly fuel DNL, representing a primary metabolic link between glucose and lipid pathways. Multiple signaling cascades coordinate this metabolic crosstalk in cancer cells, and HCC-specific regulators of glucose–lipid metabolic reprogramming are summarized in Table 1.

**Table 1:** Hepatocellular carcinoma (HCC)-specific mechanisms mediating crosstalk among glucose, lipid, and amino acid metabolic reprogramming

Crosstalk type	Molecules	Mechanisms	References
Glucose metabolism regulates lipid metabolism	Acetyl-CoA carboxylase alpha (ACCα)	ACCα releases carnitine palmitoyltransferase 1 A (CPT1A) into the mitochondria during glucose deprivation, promoting fatty acid oxidation to maintain cell survival	[306]
	Monocarboxylate transporter 1 (MCT1)	The lactate transporter MCT1 can upregulate sterol regulatory element–binding protein 1 (SREBP1) and the downstream stearoyl-CoA desaturase-1, thereby promoting unsaturated fatty acid production and conferring resistance to ferroptosis	[126]
	MCT4	High levels of MCT4 in HCC can suppress lipid peroxidation	[307]
Lipid metabolism regulates glucose metabolism	Acetyl-CoA	In non-alcoholic steatohepatitis, acetyl-CoA generated from fatty acid oxidation promotes the acetylation of malate dehydrogenase 2 and aspartate aminotransferase 2, thereby enhancing glycolysis	[308]
	CD36	The fatty acid transporter CD36 promotes glycolysis through activation of the protein kinase B (AKT)/mTOR signaling pathway	[95]
	27-hydroxycholesterol	27-hydroxycholesterol activates glucose-regulated protein 75, which in turn modulates hypoxia-inducible factor-1 α (HIF-1α), c-Myc, and glucose uptake	[195]
Signals regulate glucose and lipid metabolism	Mammalian target of rapamycin complex1 (mTORC1)	mTORC1 enhances the metabolic flux from glucose to lipid synthesis by upregulating HIF-1α and SREBP1, thereby increasing cardiolipin production and conferring resistance to ionizing radiation	[309]
	Stromal interaction molecule 1(STIM1)	In metastatic HCC cells, STIM1 is downregulated, which suppresses glycolysis while activating fatty acid oxidation, thereby promoting anoikis resistance	[176]
	Carbohydrate responsive element binding protein (ChREBP)	The transcription factor ChREBP reroutes glutamine and glucose metabolic fluxes into fatty acid and nucleic acid synthesis	[310]
Amino acids metabolism regulates glucose metabolism	AKT/forkhead box O1 signals	In oleic acid–treated HCC cells, glutamine improves glucose metabolism through activation of the serine/AKT/forkhead box O1 signaling pathway	[311]
	RNA-binding motif protein 39	In HCC, elevated arginine levels bind to RNA-binding motif protein 39, thereby regulating metabolic alterations in glucose, amino acid, nucleotide, and fatty acid pathways	[274]
	Phosphoenolpyruvate carboxykinase 1	The gluconeogenic enzyme phosphoenolpyruvate carboxykinase 1 promotes the production of S-adenosylmethionine via the serine synthesis pathway	[292]
Glucose metabolism regulates amino acids metabolism	Glucose	Under high-glucose conditions, glutamine is catabolized predominantly through aspartate aminotransferase, whereas under glucose-restricted conditions, glutamine is catabolized via glutamate dehydrogenase 1	[244]
	Fructose	High-fructose diet upregulates glutamine in HCC via gut microbiota-derived acetate	[312]
Signals regulate glucose and amino acids metabolism	Hepatocyte growth factor	Hepatocyte growth factor activates the pyruvate dehydrogenase complex and glutaminase 1 through the mesenchymal–epithelial transition factor, thereby promoting the Warburg effect and glutamine metabolism	[228]
Amino acids metabolism regulates lipid metabolism	Solute carrier family 25 member 15	In HCC, downregulation of solute carrier family 25 member 15 activates a glutamine-reactive metabolic pathway, thereby enhancing lipid biosynthesis	[313]
	RNA-binding motif protein 39	In HCC, elevated arginine levels bind to and activate RNA-binding motif protein 39, which in turn modulates lipid metabolic processes	[274]
	Glutamine	In oleic acid–treated HCC cells, glutamine suppresses the levels of ACC and fatty acid synthase	[311]
	Peroxisome proliferator-activated receptor-δ (PPARδ)	In sorafenib-resistant HCC, PPARδ mediates the upregulation of glutamine-derived lipid biosynthetic pathways, enabling resistance to oxidative stress	[314]
Lipid metabolism regulates amino acids metabolism	CPT1A	Loss of CPT1A in HCC reduces acetyl-CoA production, leading to the accumulation of branched-chain amino acids and hyperactivation of mTOR signaling	[128]
	SREBP2	Under glutamine-deprived conditions, reduced glutamine availability suppresses SREBP2, thereby inhibiting cholesterol biosynthesis	[315]
Signals regulate lipid and amino acids metabolism	ChREBP	The transcription factor ChREBP reroutes glutamine and glucose metabolic fluxes into fatty acid and nucleic acid synthesis	[310]
	Oxoglutarate dehydrogenase-like	In HCC patients, low oxoglutarate dehydrogenase-like levels reduce the degradation of glucose and amino acids, and promote the reductive carboxylation of glutamine-derived α-ketoglutaric acid as well as de novo fatty acid synthesis	[316, 317]

mTORC1 is a central driver of metabolic reprogramming, activated in cancer cells downstream of PI3K/AKT, and orchestrating both glucose and lipid metabolism. mTORC1 enhances glucose uptake and glycolysis by upregulating GLUTs, MCT1 [69], and HK2 [70]. It also promotes DNL by activating SREBP1, thereby inducing FASN, ACLY, and ACC1 levels [127, 129–131]. In addition, mTORC1 stimulates glycolysis via upregulation of HIF-1α [296, 297].

Likewise, the transcription factor c-Myc promotes both glycolysis and lipid synthesis. c-Myc increases the levels of glycolytic and PPP enzymes, including LDHA, GLUT1, PKM2, and G6PD [63, 64], and enhances DNL and cholesterol synthesis by transcriptionally activating SREBP1/2 [134].

Carbohydrate-responsive element–binding protein (ChREBP) is a glucose-sensing transcription factor that responds to intracellular glucose levels and coordinates glucose–lipid metabolism. High-glucose conditions activate ChREBP [298]. In cancer cells, ChREBP enhances glycolysis, lipogenesis, and AKT/mTOR signaling [299, 300]. Notably, ChREBP promotes the progression of NASH to HCC by simultaneously augmenting lipogenesis and glycolysis [301].

In addition, phosphofructokinase liver type, a glycolytic enzyme, has been shown to activate CPT1A, thereby promoting FAO and tumor growth [302]. CD36, a fatty acid transporter upregulated in cancer [303], further enhances glycolysis via the PI3K/AKT/mTOR pathway [95].

Glucose–lipid metabolic reprogramming in cancer cells is influenced by nutrient availability. Under high-glucose conditions, glycolysis and DNL are enhanced, whereas glucose deprivation shifts metabolism toward increased FAO [304]. AMPK acts as a metabolic switch under energy stress; during hypoxia or glucose starvation, AMPK is activated [138]. Activated AMPK suppresses glycolysis by inhibiting mTORC1 and downregulating HK2, GLUT1, and LDHA [305]. Concurrently, AMPK upregulates FAO enzymes CPT1, CPT2, and CD36 [139] and inhibits lipogenesis by repressing SREBP1 [140].

### Glucose–amino acid metabolic interplay

There is substantial crosstalk between glucose metabolism and amino acid metabolism, mediated through shared intermediates and signaling pathways. Intermediates generated from glycolysis and the TCA cycle serve as essential precursors for the synthesis of serine, glutamate, and aspartate. In cancer cells, glutamine uptake is elevated, and its conversion to α-ketoglutarate fuels the TCA cycle to support ATP production. Multiple signaling pathways further coordinate the rewiring of glucose–amino acid metabolic crosstalk in cancer. Factors and mechanisms specifically mediating glucose–amino acid metabolic reprogramming in HCC are summarized in Table 1.

Glucose and glutamine metabolism are mutually regulated, such that the deficiency of one nutrient modulates the metabolic utilization of the other. Under glutamine deprivation, cancer cells enhance glutamine uptake and glycolysis to compensate for glutamine loss [318]. Inhibition of glycolysis suppresses PKM2, diverting carbon flux toward serine biosynthesis while maintaining TCA cycle activity through glutamine metabolism [319]. Moreover, hypoxic conditions enable glutamine to potentiate glycolytic flux [320]. When both glutaminolysis and glycolysis are simultaneously blocked, ROS accumulate, triggering AMPK activation and apoptosis in cancer cells [239].

In cancer cells, aberrant activation of HIF-1α and the transcription factor c-Myc serves as a signaling axis regulating both glucose and amino acid metabolism. Under serine-deprived conditions, activated HIF-1α upregulates glycolytic enzymes and serine biosynthetic enzymes to enhance glycolysis and supply carbon substrates for increased serine synthesis. The enzymes include PHGDH, phosphoserine aminotransferase 1, phosphoserine phosphatase, GLUT1, GLUT3, and HK2 [213]. c-Myc regulates a broad spectrum of amino acid metabolic enzymes, such as GLS1 [204], PHGDH [206], ASS1, and PYCR1 [208]. c-Myc simultaneously induces glycolytic enzymes including PKM2 and G6PD [63, 64], thereby accelerating glucose and amino acid utilization.

Cancer cells also activate mTORC1 through intracellular amino acid accumulation, thereby promoting tumor progression [321]. Elevated levels of leucine, arginine, and glutamine in cancer cells are potent activators of mTORC1 [218–220]. Activated mTORC1 further enhances glycolysis by upregulating glucose transporters and glycolytic enzymes, including GLUT1 and HK2 [69, 70].

ATF4 is upregulated in cancer and functions as a regulator of amino acid metabolism. ATF4 transcriptionally induces ASNS, ASS1, PHGDH, and the glutamine transporter SLC1A5 [216]. In colorectal cancer, ATF4-mediated upregulation of SLC1A5 has been shown to further enhance the levels of PKM2 and HK2, thereby promoting glycolysis [322]. The antioxidant transcription factor NRF2 is likewise activated in cancer, where it facilitates glutamine-derived GSH synthesis and stimulates the PPP. Specifically, NRF2 upregulates GS and glutamate transporter 1 levels in cancer cells [217], and activates the PPP through induction of G6PD [323, 324]. Additionally, the NRF2/ATF4 axis has been reported to drive HK2 upregulation in cancer cells [325].

The glycolytic enzyme PKM2 plays a pivotal regulatory role in serine biosynthesis in cancer. Serine directly binds to and activates human PKM2; therefore, serine deprivation reduces PKM2 activity [326]. The resulting inhibition of glycolysis leads to the accumulation of glycolytic intermediates, thereby channeling more glucose-derived carbon into serine biosynthesis [319, 326, 327]. Separately, PKM2 enhances the conversion of glutamine to lactate in cancer cells [328].

### Lipid–amino acid metabolic interplay

Crosstalk exists between intermediates and signaling pathways of lipid and amino acid metabolism. Glutamine-derived α-KG can undergo reductive carboxylation by isocitrate dehydrogenase 1 (IDH1) to generate citrate, which subsequently supplies acetyl-CoA for DNL [329]. Conversely, fatty acid β-oxidation produces acetyl-CoA that enters the TCA cycle, yielding α-KG that can be converted into glutamate. The interplay between lipid and amino acid metabolic reprogramming is mediated by multiple signaling pathways, and HCC-specific mechanisms reported to date are detailed in Table 1.

Amino acids promote lipid synthesis primarily through activation of mTORC1. Elevated intracellular levels of leucine, arginine, and glutamine activate mTORC1 in cancer cells [218–220]. Activated mTORC1 enhances DNL by upregulating SREBP1 and suppresses FAO by inhibiting CPT1A [127, 128]. mTORC1 additionally promotes the biosynthesis of aspartate and arginine [210, 211]. Moreover, in ovarian cancer, glutamine activates the mTOR–SREBP2 pathway to increase HMGCS1, thereby contributing to membrane stability [330].

c-Myc also plays a central role in coordinating lipid and amino acid metabolic reprogramming. As discussed earlier, c-Myc enhances the biosynthesis of lipids, serine, arginine, and proline [134, 206, 208, 331]. HIF-1α is another key regulator of glucose and amino acid metabolism, upregulating multiple enzymes involved in amino acid metabolism, including GLS1 [212], SHMT2 [214], and ASNS [215]. Recently, CPT1A, the rate-limiting enzyme in FAO, was shown to activate HIF-1α and promote tumor progression in breast cancer [332].

NRF2 promotes the synthesis of GSH from glutamate and the production of monounsaturated fatty acids catalyzed by SCD1—both of which are critical mediators of ferroptosis resistance. Evidence indicates that SCD1 is upregulated in cancer via AKT-dependent signaling [333]. The glutamine transporter SLC38A5 enhances GSH synthesis through NRF2 activation and, by increasing glutamine uptake, stimulates mTORC1 and its downstream effector SCD1 [334].

In addition, upregulated ASS1 in cancer cells disrupts the AMP/ATP balance, resulting in AMPK activation and subsequent upregulation of CPT1A, thereby enhancing FAO and ATP production [173]. Another study reported that BCAA metabolism in pancreatic cancer regulates lipid homeostasis by controlling mitochondrial fatty acid import [335]. Moreover, loss of FASN in cancer cells drives reductive carboxylation of glutamine-derived α-KG to produce citrate, which is shuttled into mitochondria, thereby alleviating oxidative stress [329].

Notably, amino acids such as glutamine and arginine not only regulate lipid metabolism in HCC (Table 1) but are similarly linked to lipid accumulation in NASH. Circulating amino acid abundance influences NASH progression. Hyperhomocysteinemia induces adipocyte lipolysis and promotes hepatic lipid accumulation [336], whereas dietary citrulline supplementation attenuates fructose-induced hepatic steatosis [337]. Mechanistically analogous to cancer cells, amino acid accumulation in NAFLD activates mTORC1, leading to intracellular lipid and lipid droplet accumulation [338]. In addition, GLS1 is upregulated in NASH, enhancing antioxidant capacity but concurrently exacerbating hepatic lipid deposition [339]. Compared with patients with NASH alone, individuals with NASH-associated HCC exhibit higher serum triglyceride and AST levels [340], reflecting the more severe hepatic steatosis accompanying progression to HCC.

### Integrated metabolic networks and signaling hubs

**Fig. 4:**
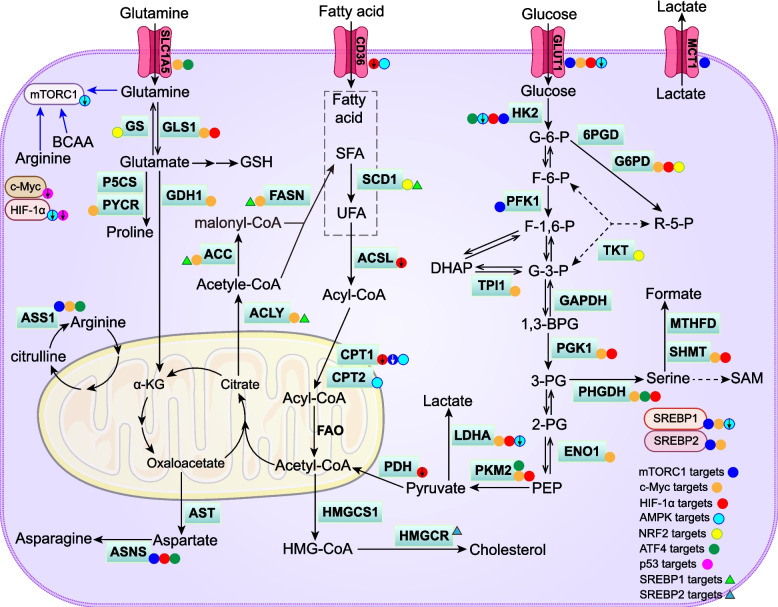
Integrated metabolic networks and signaling hubs in cancer. Carbohydrate, lipid, and amino acid metabolic pathways are directly or indirectly interconnected, forming an integrated metabolic network. mTORC1, c-Myc, and HIF-1α constitute major signaling hubs that reprogram cancer metabolism by regulating downstream metabolic enzymes, thereby enhancing nutrient transport and uptake, glycolysis, the pentose phosphate pathway, de novo lipogenesis, cholesterol synthesis, and amino acid biosynthesis. The transcription factor ATF4 further promotes glycolysis and amino acid synthesis in cancer cells. NRF2 orchestrates metabolic programs that eliminate reactive oxygen species to maintain redox balance and prevent oxidative stress–induced ferroptosis. SREBP1/2, the central regulators of lipid biosynthesis, are activated downstream of mTORC1. p53, a tumor suppressor, is downregulated in cancer cells. Although AMPK acts as a tumor suppressor in normal tissues, in cancer cells it becomes activated under glucose deprivation or energy stress, supporting cell survival. Abbreviations: 1,3-BPG, 1,3-Bisphosphoglycerate; 2-PG, 2-phosphoglycerate; 3-PG, 3-phosphoglycerate; 6PGD, 6-phosphogluconate dehydrogenase; ACC1, acetyl-CoA carboxylase 1; ACLY, ATP-citrate lyase; ACSL, acyl-CoA synthetase long-chain; α-KG, α-ketoglutarate; AMPK, AMP-activated protein kinase; ASNS, asparagine synthetase; ASS1, argininosuccinate synthase-1; AST, aspartate aminotransferase; ATF4, activating transcription factor 4; BCAA, branched-chain amino acids; c-Myc, myelocytomatosis viral oncogene homolog; CPT, carnitine palmitoyltransferase; DHAP, dihydroxyacetone phosphate; ENO1, enolase 1; F-1,6-P, fructose-1,6-bisphosphate; F-6-P, fructose 6-phosphate; FAO, fatty acid β-oxidation; FASN, fatty acid synthase; G-3-P, glyceraldehyde 3-phosphate; G-6-P, glucose 6-phosphate; G6PD, glucose-6-phosphate dehydrogenase; GAPDH, glyceraldehyde-3-phosphate dehydrogenase; GDH1, glutamate dehydrogenase 1; GLS1, glutaminase 1; GLUT1, glucose transporter 1; GS, glutamine synthetase; GSH, glutathione; HIF-1α, hypoxia-inducible factor-1α; HK2, hexokinase 2; HMG-CoA, 3-hydroxy-3-methylglutaryl-CoA; HMGCR, 3-hydroxy-3-methylglutaryl-CoA reductase; HMGCS1, 3-hydroxy-3-methylglutaryl-CoA synthase 1; LDHA, lactate dehydrogenase A; MCT1, monocarboxylate transporter 1; MTHFD, methylenetetrahydrofolate dehydrogenase; mTORC1, mammalian target of rapamycin complex 1; NRF2, nuclear factor erythroid 2–related factor 2; p53, tumor protein 53; P5CS, P5C synthase; PDH, pyruvate dehydrogenase; PEP, phosphoenolpyruvate;PFK1, phosphofructokinase 1; PGK1, phosphoglycerate kinase 1; PHGDH, phosphoglycerate dehydrogenase; PKM2, pyruvate kinase M2; PYCR, pyrroline-5-carboxylate reductase; R5P, ribose-5-phosphate; SAM, S-adenosylmethionine; SFA, saturated fatty acid; SHMT, serine hydroxymethyltransferase; SLC1A5, solute carrier family 1 member 5; SREBP, sterol regulatory element–binding protein; TKT, transketolase; TPI1, triosephosphate isomerase 1; UFA, unsaturated fatty acid

In cancer, glucose, lipid, and amino acid metabolic pathways are tightly interconnected, forming integrated metabolic networks (Fig. 4). Glycolytic intermediates serve as precursors for multiple biosynthetic pathways: glyceraldehyde 3-phosphate contributes to triglyceride synthesis. 3PG is the precursor for serine biosynthesis; and pyruvate is decarboxylated to acetyl-CoA for entry into the TCA cycle. Within the TCA cycle, citrate provides the carbon source for DNL, α-KG is interconvertible with glutamate, and oxaloacetate serves as a substrate for aspartate synthesis. The PPP generates NADPH to support DNL, cholesterol synthesis, and serine biosynthesis, while ribose-5-phosphate fuels nucleotide production. FAO generates acetyl-CoA that can enter the TCA cycle for ATP production, contribute to cholesterol biosynthesis, or serve as a donor for acetylation reactions. Glutamine-derived glutamate donates carbon for α-KG formation to sustain TCA flux, and donates amino groups for the synthesis of serine, aspartate, proline, and alanine. Serine supplies one-carbon units for sustaining SAM and formate production. Formate and aspartate are essential substrates for nucleotide biosynthesis, whereas SAM provides methyl groups for epigenetic and post-translational methylation. Furthermore, glutamine-derived GSH and SCD1-generated monounsaturated fatty acids maintain redox balance and protect against ferroptosis.

Signaling hubs orchestrate the metabolic crosstalk that underlies cancer-associated metabolic reprogramming (Fig. 4). mTORC1 functions as a central nutrient-sensing and metabolic regulatory node. mTORC1 is activated by insulin-like growth factor 1 [341] and amino acids such as leucine, arginine, methionine, and glutamine [218–220]. Once activated, mTORC1 upregulates glycolysis as well as lipid and amino acid biosynthesis by inducing multiple metabolic enzymes. The enzymes include GLUT, MCT1, HK2, 6-phosphofructo-2-kinase/fructose-2,6-bisphosphatase 3, PFK1, SREBP1, ASNS, and ASS1 [69–71, 127, 209–211].

The pro-tumorigenic transcription factor c-Myc transcriptionally activates a broad array of metabolic enzymes. These include LDHA, GLUT1, ENO1, TPI1, PGK1, PKM2, G6PD, ACC1, ACLY, FASN, SREBP1/2, SLC1A5, GLS1, GDH1, PHGDH, SHMT, ASS1, and PYCR1 [62–64, 134–136, 203–208].

HIF-1α is constitutively activated in cancer and enhances glycolysis, the PPP, and amino acid biosynthesis while suppressing FAO. Specifically, HIF-1α upregulates PKM2, GLUT1, LDHA, HK2, PDK1, PGK1, G6PD, GLS1, PHGDH, SHMT2, and ASNS [55–60, 212–215], while repressing CPT1A, ACSL1, and CD36 [170].

Prior to cancer initiation, AMPK acts as a tumor suppressor. However, once cancer is established, whether AMPK promotes or suppresses tumorigenesis depends on the cancer type and contextual conditions [342]. Under conditions of energy deficiency and ATP depletion, AMPK constrains hyperactive metabolic pathways through phosphorylation-dependent regulation. AMPK downregulates HK2, GLUT1, LDHA, SREBP1, mTORC1, and HIF-1α [140, 174, 305, 343], while upregulating CPT1, CPT2, and CD36 [139].

The antioxidant transcription factor NRF2 promotes the synthesis of GSH and monounsaturated fatty acids, critical mediators of ferroptosis resistance. NRF2 upregulates GS, glutamate transporter 1, G6PD, TKT, and SCD1 [104, 217, 323, 324, 334]. By enhancing GSH and unsaturated lipid synthesis and PPP-derived NADPH production, NRF2 facilitates ROS detoxification and protects against oxidative stress and ferroptosis.

In addition, the stress-responsive transcription factor ATF4 upregulates ASNS, ASS1, PHGDH, and SLC1A5 [216], as well as PKM2 and HK2 [322]. The tumor suppressor *TP53*, which is frequently downregulated in cancer, encodes p53—a protein that negatively regulates c-Myc and HIF-1α [66].

## Therapeutic opportunities targeting metabolic reprogramming

As outlined above, the metabolic reprogramming of glucose, lipid, and amino acid pathways in cancer is largely orchestrated by several key molecules. They regulate downstream metabolic enzymes and thereby drive tumor-associated metabolic alterations. Consequently, these metabolic enzymes represent potential therapeutic targets in cancer. Pharmacologic agents directed against these enzymes, including small-molecule inhibitors, may exert antitumor effects. A growing number of novel therapeutics aimed at targeting metabolic reprogramming are currently under development; although most remain in the preclinical stage, encouragingly, some have already progressed to clinical trials. Nevertheless, research on metabolism-targeted therapies is still evolving, and the efficacy and safety profiles of these agents vary considerably. Table 2 summarizes and comments on the current landscape of metabolism-targeted drugs under investigation for cancer treatment.

**Table 2:** Drugs Targeting Metabolic Reprogramming in Cancer

Targets	Drugs	Latest state of development	Comments	References
**Glucose transporter (GLUT)**				
GLUT1	Salvianolic acid B	Preclinical	Salvianolic acid B has sufficient preclinical research, but the depth of its association with the GLUT1 pathway is inadequate	[22]
GLUT1	Rg3-PTX-LPs	Preclinical	The drug has been well-established in vitro and in vivo experiments, with an antitumor rate of 90.3%	[344]
GLUT1	PGL13	Preclinical	This inhibitor exerts cytotoxic effects on cancer cells; further animal studies are recommended	[345]
GLUT1	BAY-876	Preclinical	GLUT1 inhibitor BAY-876 has exhibited anticancer activity in various cancer cells with development potential	[346–348]
GLUTs	KL-11743	Preclinical	The drug provides a novel and promising direction for leukemia treatment	[349]
Sodium-glucose cotransporter (SGLT)				
SGLT2	Canagliflozin	Phase I	Canagliflozin studies for pancreatic and colorectal cancer treatment are in clinical trials, suggesting developmental potential across broader malignancies	[350], NCT07076823
SGLT2	Dapagliflozin	Phase I	The therapeutic effect is limited, and most patients develop disease progression after drug withdrawal, yet the drug has favorable safety	[351]
**Glycolysis**				
Hexokinase 2 (HK2)				
HK2	ketoconazole	Preclinical	Targeting HK2 with azole antifungals for glioblastoma treatment represents a clinically translatable "old drug repurposing" strategy	[352]
HK2	HK2-ASO1	Preclinical	Selective inhibition of HK2 is an innovative therapy, yet its efficacy and safety in humans require further investigation	[353]
HK2	2-deoxy-D-glucose	Phase III	2-Deoxy-D-glucose has been clinically confirmed to selectively enhance tumor radiosensitization with excellent tolerability	[354]
HK2	Resveratrol	Phase II	It exhibits favorable safety and tolerability in clinical trials, but its therapeutic efficacy remains limited	[355]
Pyruvate kinase M2 (PKM2)				
PKM2	Sanguinarine	Preclinical	Research on the natural extract sanguinarine targeting PKM2 for cancer therapy has just begun, and it holds considerable research value	[356]
PKM2	Triclabendazole	Preclinical	Inhibiting glycolysis and suppressing lung cancer cell proliferation by blocking PKM2 nuclear localization represents a novel therapeutic mechanism	[357]
PKM2	Shikonin	Preclinical	Research on shikonin treating cancer by inhibiting PKM2 remains insufficient and incomplete	[358]
**Pentose phosphate pathway (PPP)**				
6-phosphogluconate dehydrogenase (6PGD)				
6PGD	physcion	Preclinical	The optimal combined regimen for Physcion and Metformin lacks thorough investigation and pharmacokinetic validation of in vivo dosing	[359]
6PGD	S3	Preclinical	S3 combined with Enzalutamide demonstrated marked efficacy, but its independent prognostic value requires further investigation	[360]
Glucose-6-phosphate dehydrogenase (G6PD)				
G6PD	6-aminonicotinamide	Preclinical	The clinical translation of 6-aminonicotinamide, a classic nicotinamide antagonist, is primarily hindered by its inherent systemic toxicity and narrow therapeutic window	[361]
G6PD	HuaChanSu	Phase II	Concurrent use with radiotherapy failed to improve locoregional control or survival rates in elderly or chemotherapy-ineligible esophageal squamous cell carcinoma patients	[362]
**Lactate metabolism**				
Monocarboxylate transporter (MCT)				
MCT1	AZD3965	Phase I	AZD3965 is primarily effective in patients with Burkitt lymphoma and diffuse large B-cell lymphoma characterized by low tumor MCT4 levels	[363]
MCT4	α-CHCA	Preclinical	Given that α-CHCA inhibits multiple MCT isoforms, including MCT4, further in vivo investigation should be conducted	[364]
Lactate dehydrogenase A (LDHA)				
LDHA	Quercetin	Preclinical	Given that Quercetin is a multi-target anticancer substance, this study does not demonstrate that its effects are mediated solely through LDHA inhibition	[365]
LDH	RS6212	Preclinical	RS6212 exhibits broad-spectrum anticancer activity; however, since this research constitutes preliminary in vitro studies, animal experiments are required to determine its safety and efficacy	[366]
LDHA	NHI-1	Preclinical	NHI-1 provides a direction for the treatment of chemotherapy-resistant malignant mesothelioma, yet lacks in vivo validation	[345]
LDHA	Oxamate	Preclinical	While Oxamate's efficacy benefits from its multiple synergistic mechanisms, its potential toxicity to normal cerebral cells and its therapeutic window have yet to be clarified	[367]
**Tricarboxylic acid (TCA) cycle**				
Pyruvate dehydrogenase (PDH)				
PDH	dichloroacetate	Phase II	The drug has achieved favorable results in clinical trials and is expected to advance to phases III and IV	[368]
Mutant isocitrate dehydrogenase (mIDH)				
mIDH1/2	Vorasidenib	Phase III	The trial results are historic, showing overwhelming clinical efficacy for Vorasidenib, which more than doubled the median progression-free survival	[369]
mIDH1/2	LY3410738	Phase I	LY3410738 had a limited objective response rate when administered alone, but demonstrated excellent antitumor efficacy in combination with cisplatin and gemcitabine	[370]
mIDH1	safusidenib	Phase II	Safusidenib treatment achieved a high objective response rate of up to 44.4%; however, its adverse event rate was also high, exceeding 40%	[371]
mIDH1	IDH305	Phase I	The trial was prematurely terminated due to a potentially narrow therapeutic window, which directly prevented determination of the Phase II recommended dose	[372]
mIDH1	Ivosidenib	Phase IV	The combination of ivosidenib and azacitidine demonstrates significant efficacy in treating acute myeloid leukemia and exhibits a favorable safety profile	[373]
**Fatty acid transporter**				
CD36	VT1021	Phase I	VT1021 is the only CD36-targeting agent to enter advanced clinical trials; while it demonstrated excellent safety, its objective response rate as a single agent was low	[374]
CD36	Sulfosuccinimidyl Oleate	Preclinical	The drug is highly effective and precise, demonstrating excellent targeting, though the large-scale production of this nanosystem may present a challenge	[375]
**De novo lipogenesis (DNL)**				
ATP-citrate lyase (ACLY)				
ACLY	Bempedoic Acid	Preclinical	Bempedoic acid combined with the cyclin-dependent kinase inhibitor palbociclib suppresses breast cancer, but lacks supporting animal studies and bempedoic acid monotherapy data	[376]
ACLY	SB-204990	Preclinical	Most studies of SB-204990 in cancer involve combination therapy, necessitating further research on its efficacy as a monotherapy	[377]
ACLY	BMS-303141	Preclinical	Inhibition of oesophageal squamous cell carcinoma was demonstrated; further in vivo investigation is recommended	[378]
Acetyl-CoA carboxylase 1 (ACC)				
ACC	ND-66	Preclinical	An association between ACC1 inhibition and protein acetylation was found, but studies on the clinical translation of ND-66 are lacking	[379]
ACC	Firsocostat (GS-0976)	Preclinical	Firsocostat combined with lncRNA showed anticancer effects; however, lncRNA is not suitable for drug target development	[380]
ACC	Tofacitinib	Preclinical	Tofacitinib is clinically used as an anti-inflammatory agent, ensuring its safety while its efficacy requires further investigation	[381]
Fatty acid synthase (FASN)				
FASN	TVB-2640	Phase II	TVB-2640 is the first clinical-grade FASN inhibitor to enter Phase II clinical trials, reinvigorating FASN-targeted therapy	[382], NCT03179904
FASN	orlistat	Preclinical	Given Orlistat's established safety as an approved anti-obesity agent, its use in combination did not yield effects that surpassed the efficacy of single-agent chemotherapy	[383]
FASN	Omeprazole	Phase II	Omeprazole was well tolerated and yielded a promising pathologic complete response rate of 74.4%; however, this was a single-arm study lacking a concurrent randomized controlled group	[384]
FASN	G28	Preclinical	G28, a synthetic derivative of high-safety (-)-epigallocatechin-3-gallate, showed potent anticancer effects in vitro, suggesting the need for further in vivo studies	[385]
FASN	TVB-3664	Preclinical	While TVB-2640 is approved for clinical use, its analog, TVB-3664, shows promising prospects for both efficacy and safety	[386]
FASN	Momordicine-I	Preclinical	Only the effects of momordicine-I on the expression and translation of metabolic enzymes in cancer cells have been identified; further studies on its specific antitumor mechanisms are warranted	[387]
Stearoyl-CoA desaturase (SCD)				
SCD	SSI-4	Preclinical	Sensitivity is independent of mutational profile and correlates with acute myeloid leukemia dependency on fatty acid desaturation. This suggests SSI-4 is potent across diverse leukemia subtypes	[388]
SCD1	Huangqi-Danshen decoction	Preclinical	The experiments were sufficient and reliable, but accelerating its clinical translation requires further elucidation of the precise contribution of the compound drug components	[389]
Sterol regulatory element–binding protein (SREBP)				
SREBPs	Fatostatin	Preclinical	While Fatostatin exhibits strong effects against cancer cells, the nanosystem presented in this study remains difficult to translate clinically	[390]
SREBP-1	Betulin	Preclinical	Given the numerous preclinical studies confirming Betulin's efficacy against cancer, further clinical investigation is warranted	[391, 392]
**Fatty Acid β-Oxidation (FAO)**				
Carnitine palmitoyltransferase (CPT)				
CPT1	Etomoxir	Preclinical	Etomoxir has shown efficacy in early studies of various cancer cells with considerable potential	[393, 394]
CPT1A	DHP-B	Preclinical	With sufficient in vitro and in vivo evidence laying the groundwork for preclinical development, further investigation into effective dosage and safety is recommended	[395]
Fatty acid-binding protein				
Fatty acid-binding protein 4	HTS01037	Preclinical	HTS01037 exhibited potent therapeutic efficacy in mouse models, and no significant adverse effects were observed in the treatment group compared with the control group	[396]
**Glutamine metabolism**				
Glutaminase (GLS)				
GLS1	CB-839	Phase II	CB-839 is in clinical trials for various cancers, demonstrating efficacy and safety	NCT03872427, [397]
GLS1	Compound 968	Preclinical	The drug demonstrates a multi-target mechanism of action and significant preclinical efficacy in cancer therapy, but has yet to enter clinical trials	[398]
GLS1	BPTES	Preclinical	Multiple preclinical studies show that PTBES exhibits greater therapeutic efficacy in combination regimens	[399]
Glutamine synthetase (GS)				
GS	Methionine sulfoximine	Preclinical	Due to its significant toxicity, effective methionine sulfoximine is predominantly employed as a chemical probe	[400]
**Aspartate metabolism**				
Asparagine synthetase (ASNS)				
ASNS	ASX-173	Preclinical	Preliminary data suggest that ASX-173, as an ASNS inhibitor, may hold potential in cancer treatment	[401]
ASNS	Bisabosqual A	Preclinical	Bisabosqual A overcomes the permeability limitations of previous ASNS inhibitors, exhibiting potent synergistic sensitization and multi-faceted anticancer potential	[402]
**Arginine metabolism**				
argininosuccinate synthase (ASS)				
ASS1	ADI-PEG20	Phase II/III	ADI-PEG20 exploits ASS1 deficiency in cancers, not the ASS1 molecule, as its therapeutic target	[403]
Arginase 1				
Arginase 1	CB-1158	Preclinical	Remodeling the tumor microenvironment through metabolic targeting is a novel therapeutic strategy	[404]
**Serine metabolism**				
Serine hydroxymethyltransferase (SHMT)				
SHMT2	W478	Preclinical	W478 showed potent anti-proliferative activity in esophageal cancer; further in vivo and animal studies are required	[405]
SHMT2	SHIN 1	Preclinical	The newly developed SHMT inhibitor yielded favorable results in both in vitro and in vivo experiments	[406]
phosphoglycerate dehydrogenase (PHGDH)				
PHGDH	NCT-503	Preclinical	This study is innovative by integrating Cuproptosis with metabolic reprogramming for cancer therapy	[407]
PHGDH	CBR-5884	Preclinical	Utilizing patient-derived organoid models is beneficial for accelerating the clinical development of this drug	[408]

Metabolic reprogramming of glucose, lipids, and amino acids in HCC promotes tumorigenesis, metastasis, and drug resistance. Targeting metabolic reprogramming in HCC and developing inhibitors of metabolic enzymes are thus promising therapeutic strategies. In recent years, several such agents have been identified or developed. Table 3 summarizes the mechanisms of action of these agents and provides a critical evaluation of their relative therapeutic efficacy.

**Table 3:** Drugs targeting metabolic reprogramming for hepatocellular carcinoma (HCC) treatment in preclinical studies

Drugs	Mechanisms	comments	References
**Targeting glucose metabolism**			
Canagliflozin	Canagliflozin, a sodium-glucose cotransporter 2 inhibitor, suppresses glucose uptake and glycolysis while inducing apoptosis	Although effective against specific cancer types, canagliflozin is ineffective in sodium-glucose cotransporter 2-negative cells, such as HLE, necessitating further investigation into its efficacy	[85]
Shikonin	Shikonin, a pyruvate kinase M2 (PKM2) inhibitor, attenuates glycolysis and suppresses cancer cell growth and metastasis	Shikonin significantly inhibited HCC growth in an orthotopic xenograft mouse model without notable side effects. This finding suggests that Shikonin, a compound derived from traditional Chinese medicine, warrants further clinical trials	[409]
Quercetin	Quercetin reduces hexokinase 2 (HK2) levels in vitro and in vivo, inhibiting the growth of HCC xenografts	Quercetin exhibits notable antitumor efficacy and safety in animal models; however, as a natural flavonoid, it is limited by low oral bioavailability and rapid metabolism	[410]
Astragalin	Astragalin suppresses HK2 via miR-125b upregulation, inhibiting glycolysis, which leads to reactive oxygen species (ROS) accumulation and consequently suppresses HCC proliferation	Astragalin demonstrated favorable anticancer effects in animal studies. As a quercetin derivative, Astragalin exhibits improved water solubility and higher oral bioavailability compared to quercetin	[411]
[Cu(ttpy-tpp)Br2]Br	The drug interferes with HK2 function, blocking glycolysis and inducing mitochondrial damage	The agent demonstrates significant effects on both glycolysis and mitochondrial function. Although no substantial side effects were observed in mice, the long-term risk of copper ion accumulation remains unclear	[412]
Ilicicolin H	Ilicicolin inhibits glycolysis and lactate production by suppressing phosphoglycerate kinase 1	Targeting phosphoglycerate kinase 1 is a novel mechanism, as few inhibitors have been reported. Given that only in vitro experiments have been conducted, further in vivo studies are warranted	[413]
Bruceine D	Bruceine D induces β-catenin degradation, leading to hypoxia-inducible factor-1α (HIF-1α) downregulation and subsequent inhibition of glycolysis	Bruceine D is a natural compound derived from Brucea javanica, whose parent extract is already used clinically. While it is theoretically low in toxicity, further experimental validation of its safety is required	[414]
Interferon-alpha (IFNα)	IFNα inhibits HIF-1α and glucose metabolism, creating a hyperglycemic tumor microenvironment that promotes T cell function and enhances the efficacy of anti-programmed cell death protein 1 therapy	This strategy combines metabolic and immunotherapies. Given that both IFNα and anti-programmed cell death protein 1 agents are already clinically approved, its safety profile is considered reliable	[415]
Biguanides	Biguanides inhibit mitochondrial respiration; combining biguanides with glycolysis inhibitors may enhance therapeutic efficacy	Only in vitro studies have been conducted, lacking in vivo validation. Since the glycolysis inhibitor was unspecified, the efficacy and safety of the combined therapy remain undetermined	[416]
Osthole	Osthole combined with radiotherapy inactivates mammalian target of rapamycin and downregulates intracellular glucose transporter 1 (GLUT1), GLUT3, and PKM2	As the therapeutic mechanism was solely investigated in vitro, in vivo animal studies are recommended to clarify efficacy and safety	[76]
**Targeting lipid metabolism**			
Aspirin	Aspirin reduces nuclear factor-κB in HCC cells and downregulates acyl-CoA synthetase long-chain 1, thereby disrupting cancer cell lipid metabolism	While aspirin's effect on lipid metabolism is confirmed, direct anticancer efficacy remains unproven. Further studies are required to assess its impact on cancer cell proliferation, metastasis, and its effectiveness in animal models	[417]
Aramchol	The stearoyl-CoA desaturase 1 inhibitor aramchol blocks unconventional prefoldin RPB5 interactor mediated resistance to tyrosine kinase inhibitors, thereby enhancing tyrosine kinase inhibitor efficacy	The combination therapy demonstrates significant anticancer effects. Given that aramchol has shown a favorable safety profile in Phase II clinical trials for non-alcoholic steatohepatitis (NASH) treatment, this therapeutic strategy has high potential for clinical translation	[418]
Argatroban (drug for NASH)	MORF4-related gene on chromosome 15 rhythmically activates the transcription of lipid metabolism genes. Its inhibitor argatroban alleviates hepatic steatosis	Argatroban is an FDA-approved drug; however, its original anticoagulant effect presents a potential side effect in this context	[419]
Reproterol (drug for NASH)	Reproterol accelerates lipolysis and fatty acid oxidation, suppressing NASH	Reproterol is an FDA-approved anti-asthmatic agent, ensuring its safety profile. However, the study involved short-term treatment (only six weeks); thus, the efficacy of long-term administration requires further validation	[420]
**Targeting amino acid metabolism**			
Ginsenoside Rk1	Rk1 downregulates the levels of glutaminase 1, reducing glutathione production and leading to ROS accumulation	Rk1 has demonstrated favorable anticancer efficacy and safety in both in vitro and in vivo experiments	[230]
AG-221	AG-221 impairs the stability of the glutamine transporter alanine-serine-cysteine transporter 2 and inhibits HCC progression	AG-221 exhibits potent anticancer activity in preclinical studies. Approved for acute myeloid leukemia with established safety, it has low clinical translation barriers	[421]
Crisantaspase and methionine sulfoximine	Crisantaspase degrades serum glutamine, and methionine sulfoximine inhibits hepatic glutamine synthetase activity; the combination therapy thus blocks glutamine supply	The dual blockade completely arrested tumor growth, demonstrating significant efficacy. However, the treatment is only effective in catenin β1 mutant HCC, limiting its clinical applicability	[422]
RX108	The Na +/K + -ATPase inhibitor RX108 suppresses solute carrier family 1 member 5-mediated glutamine uptake and inhibits HCC growth	The in vivo and in vitro data for this experiment are consistent, and RX108 is already in clinical trials for solid tumors, demonstrating significant developmental promise	[423]
Asparaginase and lenvatinib	Asparaginase degrades serum glutamine and asparagine required by cancer cells. Combining asparaginase with the tyrosine kinase inhibitor lenvatinib induces oxidative stress	This combination prevents the emergence of single-agent resistance. Given that both asparaginase and lenvatinib are already utilized clinically, this combination strategy holds immense potential for clinical translation	[256]
Codelivery of CB-839 and buthionine sulfoximine	The use of a hafnium-based metal–organic framework for codelivery of CB-839 and buthionine sulfoximine synergistically inhibits glutamine metabolism, leading to ROS accumulation	This formulation offers multi-drug synergy and targeting capability. While the UiO-66 nanomaterial exhibits relatively good biocompatibility, the safety profile of its hafnium component requires further clarification	[424]
Glutamine restriction and pyruvate dehydrogenase inhibition	Dietary glutamine restriction or the use of a glutaminase inhibitor BPTES enhances the anticancer effects of a dehydrogenase inhibitor CPI-613	The therapeutic strategy demonstrated excellent targeting, efficacy, and safety in experiments, warranting further clinical trials	[425]
GCN2iB and ABT-263	Dietary arginine restriction, combined with the general control nonderepressible 2 inhibitor GCN2iB, and the senolytic compound ABT-263, constitutes a triple therapy that induces cellular senescence and death	This regimen effectively suppressed tumor proliferation in both in vitro and in vivo experiments without notable side effects. However, its efficacy is restricted to urea cycle-deficient HCC, limiting its clinical applicability	[426]

Numerous therapeutic targets related to metabolic reprogramming in cancer have been identified in recent years. However, some of these targets have only been studied at the gene expression level, without the development of drugs targeting them. We list several target molecules with high drug development potential (Table 4) that may represent avenues for future research.

**Table 4:** several target molecules with high drug development potential

Potential targets	Mechanisms	References
Adrenoceptor beta-2	Adrenoceptor beta-2 knockdown chimeric antigen receptor T cells exhibited enhanced cytotoxicity against prostate cancer cell lines in vitro, by increasing CD69, CD107a, interferon-γ, and glucose transporter 1 (GLUT1)	[427]
GLUT3	Knockdown of GLUT3 effectively inhibits the anaerobic utilization of pyruvate and reduces cell proliferation rate	[428]
circZBTB44	circZBTB44 upregulates hexokinase 3 levels by binding to insulin-like growth factor 2 mRNA-binding protein 3, exerting an oncogenic effect	[429]
phosphoenolpyruvate carboxykinase-2	Inhibition of phosphoenolpyruvate carboxykinase-2 suppresses triple-negative breast cancer proliferation and decreases metabolic flux through pyruvate carboxylase	[430]
Succinate dehydrogenase (SDH)	As SDH subunits were overexpressed in cancer tissues compared to adjacent normal tissues, screening for SDH inhibitors represents a promising therapeutic strategy	[431]
F-box-only protein 7	F-box-only protein 7 downregulation suppresses phosphoglycerate dehydrogenase activation by inhibiting protein arginine methyltransferase 1, thereby curtailing serine synthesis and inhibiting hepatocellular carcinoma (HCC) cell growth	[432]
Glycine decarboxylase	Glycine decarboxylase participates in glycine oxidation, transfers one-carbon units, and supplies precursors for nucleotide synthesis, and its loss inhibits HCC	[433]
Transcription factor 19/cyclin dependent kinase inhibitor 2A	Transcription factor 19 enhances the levels of cyclin dependent kinase inhibitor 2 A, thereby activating glycolysis and promoting the development of osteosarcoma	[434]
G protein-coupled receptors/adrenomedullin	G protein-coupled receptors-mediated high levels of adrenomedullin are directly associated with the activation of metabolic reprogramming in lung adenocarcinoma. Knockdown of adrenomedullin significantly inhibits the proliferation of lung adenocarcinoma cells	[435]
transmembrane protease serine 11B	transmembrane protease serine 11B inhibits lactate uptake. Low levels of transmembrane protease serine 11B are a marker for highly aggressive pancreatic ductal adenocarcinoma	[436]
Solute carrier family 7 member 5	The amino acid transporter solute carrier family 7 member 5 upregulation sustains cancer cell growth	[437]
Peroxisome proliferator-activated receptor δ (PPARδ)	PPARδ contributes to sorafenib resistance in HCC by enhancing glutamine metabolism and reductive carboxylation of glutamine; thus, inhibiting PPARδ overcomes sorafenib resistance	[314]
Glutaminase	Glutaminase inhibitors can enhance the sensitivity of solute carrier family 25 member 15-deficient HCC to programmed death-ligand 1 inhibitors	[313]
Aspartate aminotransferase (AST1)	Under glutamine-deprived conditions, c-Myc abundance increases, thereby promoting AST1 transcription, which maintains glutathione synthesis and inhibits ferroptosis. Inhibiting AST1 enhances the therapeutic efficacy of glutamine deprivation	[438]
Interferon-related developmental regulator 1	The glutaminase 1 selective inhibitor CB-839, when combined with interferon-related developmental regulator 1 deficiency, can induce cellular autophagy and death	[439]

### Targeting glucose metabolism

Metabolic reprogramming of glucose is essential for cancer progression, and therapeutic strategies targeting glucose uptake, glycolysis, the PPP, and lactate metabolism hold substantial promise (Table 2 and Table 3). Antidiabetic SGLT2 inhibitors such as canagliflozin and dapagliflozin were initially developed for glucose-lowering therapy. They have now entered Phase I clinical investigations in multiple malignancies [85, 350, 351], supported by their well-established clinical safety profiles. Moreover, several GLUT inhibitors are under active development and demonstrate potent anticancer activity in preclinical models, although none have yet progressed to clinical evaluation [344, 346–349]. Given the markedly elevated levels of HK2 in cancer cells relative to normal tissues, HK2-targeted agents exhibit limited off-target toxicity. For instance, 2-deoxy-D-glucose and resveratrol have advanced to Phase II–III clinical trials with favorable tolerability [354, 355]. By contrast, therapeutic targeting of PKM2 remains at an early developmental stage [356, 358].

Because normal cells retain dependence on the PPP, systemic toxicity remains a major barrier to clinical translation of PPP inhibitors [361]. Combinatorial strategies integrating PPP blockade with established anticancer agents may, however, enhance therapeutic efficacy [359, 362]. The accumulation of lactate and increased lactate production represent metabolic hallmarks of cancer, making MCT and LDHA inhibition attractive strategies. MCT1 inhibitors have advanced to Phase I trials [363], whereas LDHA-targeted therapies remain largely preclinical [345, 366].

mIDH inhibitors represent a landmark example of successful clinical translation of metabolism-targeted therapies (Table 2). Because mIDH mutations are largely tumor-specific and minimally involved in essential metabolic pathways in normal tissues, mIDH inhibitors act with high precision and therapeutic selectivity. Multiple mIDH inhibitors have already achieved clinical approval [373, 440], and several additional agents are approaching regulatory submission [345, 366].

### Targeting lipid metabolism

Lipid metabolism plays essential roles in membrane biogenesis, energy production, and the maintenance of redox homeostasis. Targeting lipid uptake, DNL, and FAO has therefore emerged as a promising therapeutic strategy (Table 2). Drug development directed at lipid uptake remains relatively limited; VT1021 is currently the only CD36-targeting agent that has advanced into late-stage clinical trials. Although VT1021 exhibits an excellent safety profile, its objective antitumor activity has been modest [374].

Substantial efforts have been devoted to targeting key DNL enzymes such as ACC and ACLY, but clinical translation has progressed slowly. ACLY inhibitors demonstrate improved therapeutic efficacy in combination regimens [376, 377], whereas the anticancer effects of some ACC inhibitors require further validation [379, 381]. In contrast, drug development targeting FASN has advanced rapidly. Although no FASN inhibitor has yet received regulatory approval, the FASN inhibitor TVB-2640, alone or combined with omeprazole, has progressed to Phase II clinical trials [382, 384]. The trials have shown encouraging results, suggesting that clinical application may be forthcoming. Additionally, several preclinical FASN inhibitors exhibit strong potential for future clinical testing [385, 386]. Inhibition of SCD1, which reduces the production of monounsaturated fatty acids, also represents a feasible therapeutic approach [388, 389]. Therapies directed at FAO have shown substantial antitumor efficacy with limited adverse effects, highlighting their translational potential [393, 394]. However, the potential cardiotoxicity associated with CPT1 inhibition warrants careful consideration.

Notably, lipid metabolism–targeted agents have therapeutic relevance not only to cancers, including HCC, but also to metabolic liver diseases such as NASH (Table 3). Argatroban and Reproterol, both FDA-approved for other indications, enhance lipolysis and reduce lipid accumulation, and are currently under investigation for NASH treatment [419, 420].

### Targeting amino acid metabolism

Targeting glutamine, asparagine, arginine, and serine metabolism in cancer therapy has achieved notable progress (Table 2). Glutamine metabolism supports nucleotide synthesis and the production of the antioxidant GSH, making it an important therapeutic target in cancer. Multiple GLS1-targeting agents are under development and have demonstrated promising anticancer activity in preclinical studies [398, 399]. Among them, CB-839 has advanced to Phase II clinical trials (NCT03872427), where its efficacy and safety have been confirmed [397]. Similarly, inhibitors of the glutamine transporter SLC1A5 show therapeutic potential, particularly for HCC [421, 423]. By contrast, GS inhibitors exhibit substantial toxicity and are generally limited to use as chemical probes, making clinical translation difficult [400].

Asparaginase, which hydrolyzes circulating asparagine, is clinically effective in leukemia, where cancer cells lack the capacity for de novo asparagine synthesis. However, asparaginase shows little efficacy against solid tumors, which typically have high ASNS levels [441]. Pharmacologic inhibition of ASNS for solid tumor therapy remains in the preclinical stage [401, 402]. Serine metabolism provides essential substrates for nucleotide biosynthesis; thus, targeting PHGDH or SHMT to block DNA synthesis and epigenetic regulation represents a rational therapeutic strategy. Several inhibitors of serine metabolism have shown favorable efficacy and safety profiles, although all remain in preclinical development [406, 408]. Given that PHGDH and SHMT are also required in normal tissues, potential toxicities warrant careful evaluation.

Amino acid–restricted diets have emerged as a novel therapeutic approach based on the higher amino acid demand of cancer cells compared with normal cells. Current dietary interventions are being explored primarily as adjuvant strategies to potentiate the effects of anticancer therapies [425, 426].

### Combinatorial approaches targeting multiple pathways

Reports of single agents that simultaneously target multiple metabolic pathways in cancer are limited. The ChREBP inhibitor SBI-993 suppresses the PI3K/AKT pathway, thereby inhibiting glycolysis, glutamine metabolism, and lipogenesis, ultimately restraining HCC growth [310]. In addition, newly developed metformin–loaded hyaluronic acid–derived carbon dots inhibit GLS1 and downregulate GLUT1. The drug leads to glutamine and glucose deprivation, intracellular ROS accumulation, and subsequent cancer cell apoptosis [239]. The serine-cysteine transporter 2 promotes DNL in cancer cells, and its inhibitor C118P can suppress lipid metabolism [442]. Moreover, nanomaterial-based delivery of siRNA targeting both GLS1 and glycolytic enzymes effectively impedes tumor growth [443]. These multi-pathway metabolic therapeutics remain in the preclinical stage but show promising potential for further development.

Targeting multiple metabolic pathways through combination therapy also shows therapeutic potential in cancer. The mTOR inhibitor everolimus, when combined with the oral GLS inhibitor telaglenastat, simultaneously targets glucose and glutamine metabolism. This combination has completed a phase I clinical trial in renal cell carcinoma, demonstrating encouraging clinical activity and tolerability [444, 445]. Co-administration of sodium oxamate, which inhibits glycolysis and lipid synthesis, can enhance the efficacy of conventional chemotherapy [304]. In addition, inhibition of MET suppresses the Warburg effect and glutamine catabolism, leading to oxidative stress. Combining a MET inhibitor with an autophagy inhibitor further augments cancer cell damage [228]. Canagliflozin downregulates PKM2 in HCC, indirectly decreasing GLS1 and inducing ferroptosis, thereby sensitizing cancer cells to cisplatin [232].

### Early diagnosisviatargeting metabolic reprogramming

Tumor cells exhibit characteristic alterations in metabolic enzyme production, which, in principle, may be leveraged for diagnostic purposes. However, these changes are difficult to detect directly, as enzyme assessment generally requires biopsy and immunohistochemical analysis. In contrast, targeted imaging modalities such as positron emission tomography (PET) provide a safe and noninvasive means for tumor detection and localization. Fluorodeoxyglucose is transported by GLUT but does not undergo glycolysis. Thus, the radiotracer 18F-fluorodeoxyglucose, widely used for the diagnosis of various tumors, accumulates in glucose-avid lesions and is visualized by PET imaging [446–450]. Numerous cancer-specific PET tracers are under development, including probes targeting metabolic enzymes. For example, [89Zr]11B6-PET targets the prostate cancer–associated enzyme HK2 [451]. DASA-23, an 18F-labeled PKM2-specific radiotracer, selectively accumulates in PKM2-high tumors [452].

In recent years, numerous cancer-associated alterations in metabolic enzymes have been identified, providing potential diagnostic and differential diagnostic value. For example, GLUT1 is highly upregulated in myxofibrosarcoma, enabling distinction from morphologically similar nodular fasciitis [453]. The lipolytic enzymes ACSL3 and ACSL4 are specifically upregulated in HCC; increased ACSL3 distinguishes tumors from normal liver tissue, whereas ACSL4 differentiates HCC from other tumor types [177]. In addition, upstream regulators of metabolic enzymes also represent promising diagnostic targets. For instance, p52-ZER6 enhances G6PD transcription in tumor cells [454]. Dihydrolipoyl transacetylase, which is upregulated in HCC, correlates positively with GLUT1 and promotes glycolysis [455].

Tumor metabolism alters the abundance of metabolites in both tumor tissues and blood, a feature that has been explored for early cancer detection [456]. In tumor tissues, several metabolites show characteristic changes. For instance, γ-aminobutyric acid is markedly elevated in oral squamous cell carcinoma and pancreatic ductal adenocarcinoma compared with adjacent non-tumor tissues [456, 457]. Cysteine is also highly enriched in colorectal tumors relative to paired non-neoplastic tissues [458].

Blood-based assays provide a non-invasive and convenient diagnostic approach, with particular value in the early detection of HCC. Alpha-fetoprotein is widely used as an auxiliary diagnostic marker; however, serum GS levels have been reported to exhibit greater sensitivity, enabling the detection of alpha-fetoprotein–negative HCC [246]. Plasma phosphatidylcholine is decreased in HCC patients, and its concentration may help identify the risk of HCC among HBV-related liver disease patients [459]. Furthermore, lipid metabolic reprogramming in HCC leads to elevated serum angiopoietin-like protein 6, which effectively distinguishes non-malignant HBV patients from alpha-fetoprotein–normal individuals with early HBV-associated HCC [460].

### Challenges faced by metabolism-targeted therapy

Although research on the mechanisms of metabolic reprogramming in cancer and targeted therapeutic drugs has gained significant momentum in recent years, challenges in clinical translation are evident from previous texts. Off-target effects and toxicity to normal tissues remain major challenges for metabolic therapies. For instance, the CPT1A inhibitor Etomoxir has not been widely used in cancer clinical trials. The reason is that Etomoxir exerts adverse effects on the heart, liver, and insulin sensitivity [395] and suppresses T-cell proliferation [461]. However, 2-DG, currently in clinical trials, induces adverse events such as reversible hyperglycemia and gastrointestinal bleeding only at high doses [462], while lower doses are generally well tolerated.

Another potential challenge is the development of drug resistance due to metabolic plasticity. For example, after GLS inhibitors suppress glutamine breakdown, cancer cells may increase glucose uptake via glycolysis or activate aspartate-mediated pathways to maintain the TCA cycle. Similarly, using glycolysis inhibitors may lead to compensatory enhancement of FAO, providing acetyl-CoA for the TCA cycle. In addition, high levels of HK2 render cells resistant to glycolysis inhibitors [463]. Fortunately, current research has found that FASN inhibitors can be used to counteract resistance to glycolytic inhibitors, while glycolysis inhibitors can be combined to overcome resistance to GLS inhibitors. Therefore, approaches such as combining multiple metabolism-targeting drugs may help address resistance development via metabolic plasticity.

### Emerging strategies: immunometabolism and metabolomics

Recent studies have extensively investigated the effect of metabolic reprogramming on tumor immunity. In HCC, immune cell functionality is associated with dysregulated lipid metabolism [464]. Obesity promotes hepatocarcinogenesis by remodeling an immunosuppressive microenvironment through lipid dysregulation and metabolic reprogramming [465]. Emerging strategies to enhance immune response rates and overcome immune resistance in HCC via metabolic interventions are under investigation [464, 466]. Immunotherapy is increasingly being evaluated as a primary therapeutic strategy for unresectable HCC [467]. The rational integration of metabolic strategies with existing targeted therapies or immunotherapies exhibits significant therapeutic potential. For example, treatment with the CD36 inhibitor Sulfosuccinimidyl Oleate upregulated the cancer cell surface levels of major histocompatibility complex class II. The drug also upregulated programmed death-ligand 1 and augmented T cell proliferation [468]. Another CD36 inhibitor, PLT012, increased the ratio of CD8⁺ T cells to regulatory T cells and shifted TME from an "immunosuppressive type" to an "immunosupportive type" [469]. Regarding glucose metabolism, the highly enriched glycolytic pathway in tumor cells generates copious lactate, which inhibits the anticancer immunity of CD4⁺ T and CD8⁺ T cells. Interrupting glycolysis via treatment with sodium oxamate activates T cells, thereby exerting potent anticancer efficacy [37, 38]. Encouragingly, a few drugs capable of targeting metabolic reprogramming to remodel the TME and reverse immunosuppression have now entered clinical studies (NCT02071862, NCT01791595, NCT03414229). These include the GLS1 inhibitor CB-839, the MCT4 inhibitor AZD3965, and the indole 2,3-dioxygenase 1 inhibitor Epacadostat [470–472]. Although distinguishing whether their anticancer effects arise from immunometabolic modulation or direct metabolic inhibition is challenging, their efficacy and safety have been validated in clinical trials.

Metabolomics has recently offered great scientific support for identifying new therapeutic targets and enhancing conventional treatment approaches. Metabolomics also guides post-treatment monitoring and prognosis by quantitatively analyzing small molecule metabolite profiles in HCC tissues, blood, or urine. In the development of new therapies, metabolomics has identified numerous therapeutic targets for aberrant metabolic pathways by systematically comparing the metabolic differences between cancer and surrounding tissues. The targets include hexokinase domain containing 1, lysophosphatidylcholine acyltransferase 1, ceramide synthase 5, and OCTN2 [115, 473, 474]. Metabolomics has been widely applied in improving traditional treatment methods by studying how to alleviate the toxic side effects of traditional therapeutic drugs such as sorafenib on HCC patients [168]. Moreover, metabolomics overcomes drug resistance in advanced HCC patients [475–477], and assesses tolerance to radiation therapy [478]. In terms of treatment monitoring and prognosis, metabolomics can be integrated with other emerging technologies such as liquid biopsy and spatial transcriptomics. This combination enables personalized treatment monitoring and tailored intervention strategies, ultimately helping reduce the recurrence rate of HCC patients after resection [479].

## Conclusion and future directions

Metabolic reprogramming is widely recognized as a hallmark of cancer and plays a central role in the initiation, progression, and therapeutic resistance of HCC. This review summarizes the key features and underlying mechanisms of glucose, lipid, and amino acid metabolic reprogramming in cancer. Cancer cells exhibit enhanced uptake of glucose, lipids, and amino acids, along with increased glycolysis, pentose phosphate pathway activity, de novo lipogenesis, cholesterol biosynthesis, and amino acid biosynthesis. In contrast, OXPHOS and FAO may be either enhanced or suppressed depending on cancer type and microenvironmental context, while gluconeogenesis is generally inhibited. These metabolic alterations are orchestrated by major signaling pathways, including HIF-1α, c-Myc, PI3K/AKT/mTOR, NRF2, ATF4, and SREBP1. Metabolic reprogramming supplies biosynthetic precursors and energy to support tumor proliferation, activates oncogenic signaling, protects against oxidative stress and apoptosis, enhances adaptation to the tumor microenvironment, and promotes the development of drug resistance. We further summarize therapeutic agents under development that target glucose, lipid, and amino acid metabolism in cancer. Notably, several metabolism-targeted agents have entered clinical trials, highlighting their potential for further development and clinical application.

Importantly, accumulating evidence highlights metabolic reprogramming as a critical driver of therapeutic resistance in HCC. Enhanced glycolysis, increased fatty acid synthesis, cholesterol accumulation, elevated GLS levels, and increased glutamine uptake contribute to resistance to anticancer drugs in HCC. For example, cholesterol accumulation leads to tyrosine kinase inhibitor resistance [189]. Cisplatin resistance in HCC cells is mediated by enhanced GLS synthesis and glutamine uptake [229]. In HCC, loss of EVA1A upregulates CD36 expression, enhances fatty acid uptake, and confers resistance to lenvatinib [147, 480]. Encouragingly, several therapeutic strategies have emerged that target metabolic reprogramming to overcome anticancer drug resistance. The SCD inhibitor aramchol can reduce donafenib resistance in HCC [418]. The phase II FASN inhibitor TVB-2640 reverses cisplatin resistance in cervical cancer and endocrine resistance in breast cancer when used in combination therapy [481, 482]. Similarly, the GLS1 inhibitor CB-839, also currently in Phase II clinical trials, is being investigated for its ability to reverse cisplatin resistance [483] and treat osimertinib-resistant lung cancer [484]. The HK2 inhibitor 2-DG, which has advanced to Phase III for some cancers, can treat imatinib-resistant leukemic cells [485]. Moreover, inhibition of PPARδ abolishes its driving effect on glutamine metabolism and simultaneously disrupts its crosstalk regulation of the PPP and lipid synthesis pathway, thereby reversing sorafenib resistance in HCC [314]. Collectively, some of these agents have advanced into clinical trials and are poised to complete efficacy and safety studies, facilitating their transition into clinical use.

Future studies should focus on refining metabolic‐targeted therapies to improve tumor selectivity while minimizing systemic toxicity. Inhibitors targeting glycolysis, lipid synthesis, and amino acid metabolism have shown encouraging activity in preclinical and early clinical studies. However, the essential roles of many metabolic pathways in normal tissues underscore the need for careful therapeutic selection. Tumor‐specific metabolic dependencies, such as mIDH, HK2, or FASN, represent particularly promising targets with favorable therapeutic windows.

Given the pronounced metabolic plasticity of cancer cells, monotherapies are often insufficient or may induce resistance. Rational combination strategies that simultaneously target multiple metabolic pathways, or integrate metabolic inhibitors with chemotherapy, targeted therapy, or immunotherapy, warrant further investigation. Emerging multi-pathway inhibitors, nanotechnology-based drug delivery systems, and RNA-based approaches offer innovative platforms for improving safety and overcoming therapeutic resistance. Increasing evidence highlights immunometabolism as a key regulator of the tumor microenvironment and therapeutic responses in HCC. Interventions that remodel glucose and lipid metabolism have shown potential to restore antitumor immunity and enhance immune checkpoint blockade. In addition, metabolomics has emerged as a powerful tool for identifying novel metabolic vulnerabilities, monitoring treatment responses, and elucidating mechanisms of resistance.

Overall, targeting metabolic reprogramming has evolved from a conceptual framework into a clinically actionable strategy. While challenges such as toxicity, metabolic plasticity, and patient heterogeneity remain, rational combination therapies, immunometabolic interventions, and metabolomics-guided precision medicine are poised to define the next phase of metabolism-based cancer therapy.

## Data Availability

Not applicable.

## Abbreviations

1,3-BPG: 1,3-Bisphosphoglycerate
2-PG: 2-Phosphoglycerate
27HC: 27-Hydroxycholesterol
3-PG: 3-Phosphoglycerate
6PGD: 6-Phosphogluconate dehydrogenase
ACC1: Acetyl-CoA carboxylase 1
ACLY: ATP-citrate lyase
ACSL: Acyl-CoA synthetase long-chain
AKR: Aldo-keto reductase
AKR1C2: Aldo-keto reductase family 1 member C2
AKT: Protein kinase B
ALT: Alanine aminotransferase
AMPK: AMP-activated protein kinase
AREs: Antioxidant response elements
ASNS: Asparagine synthetase
ASS1: Argininosuccinate synthase-1
AST: Aspartate aminotransferase
ATF4: Activating transcription factor 4
BACH1: BTB and CNC homolog 1
BCAA: Branched-chain amino acids
BCAT: Branched-chain amino acid transaminase
BCKAs: Branched-chain α-keto acids
BCKDH: Branched-chain α-keto acid dehydrogenase
BCKDK: Branched-chain α-keto acid dehydrogenase kinase
CCL2: C-C motif chemokine ligand 2
CD: Cluster of differentiation
ChREBP: Carbohydrate-responsive element–binding protein
c-Myc: Myelocytomatosis viral oncogene homolog
CoA: Coenzyme A
CPT: Carnitine palmitoyltransferase
DHAP: Dihydroxyacetone phosphate
DNL: De novo lipogenesis
ENO1: Enolase 1
EVA1A: Eva-1 homolog A
F-1,6-P: Fructose-1,6-bisphosphate
F-6-P: Fructose 6-phosphate
FABP5: Fatty acid-binding protein 5
FAO: Fatty acid β-oxidation
FASN: Fatty acid synthase
G-3-P: Glyceraldehyde 3-phosphate
G-6-P: Glucose 6-phosphate
G6PD: Glucose-6-phosphate dehydrogenase
GAPDH: Glyceraldehyde-3-phosphate dehydrogenase
GDH1: Glutamate dehydrogenase 1
GLS: Glutaminase
GLUT: Glucose transporter
GPC3: Proteoglycan glypican-3
GPX4: Glutathione peroxidase 4
GRP75: Glucose-regulated protein 75
GS: Glutamine synthetase
GSH: Glutathione
HBV: Hepatitis B virus
HBXIP: Hepatitis B X-interacting protein
HCC: Hepatocellular carcinoma
HCV: Hepatitis C virus
HGF: Hepatocyte growth factor
HIF-1α: Hypoxia-inducible factor-1α
HK2: Hexokinase 2
HMG: 3-Hydroxy-3-methylglutaryl
HMGB1: High-mobility group box 1
HMGCR: 3-Hydroxy-3-methylglutaryl-CoA reductase
HMGCS1: 3-Hydroxy-3-methylglutaryl-CoA synthase 1
ID1: Inhibitor of differentiation 1
IDH: Isocitrate dehydrogenase
IFN: Interferon
IGFBP3: Insulin-like growth factor binding protein-3
KEAP1: Kelch-like ECH-associated protein 1
KRAS: Kirsten rat sarcoma viral oncogene homolog
LDHA: Lactate dehydrogenase A
MAT: Methionine adenosyltransferase
MCT1: Monocarboxylate transporter 1
MET: Mesenchymal-epithelial transition factor
METTL5: Methyltransferase-like 5
mIDH: Mutant isocitrate dehydrogenase
MTHFD: Methylenetetrahydrofolate dehydrogenase
mTOR: Mammalian target of rapamycin
mTORC1: Mammalian target of rapamycin complex 1
NADPH: Nicotinamide adenine dinucleotide phosphate
NAFLD: Non-alcoholic fatty liver disease
NAMPT: Nicotinamide phosphoribosyltransferase
NASH: Non-alcoholic steatohepatitis
NEAT1: Nuclear enriched abundant transcript 1
NOP2: Nucleolar protein 2
NRF2: Nuclear factor erythroid 2–related factor 2
OCTN2: Organic cation/carnitine transporter 2
OXPHOS: Oxidative phosphorylation
P5C: Pyrroline-5-carboxylate
P5CS: P5C synthase
PCK: Phosphoenolpyruvate carboxykinase
PDH: Pyruvate dehydrogenase
PDK1: Pyruvate dehydrogenase kinase 1
PEP: Phosphoenolpyruvate
PET: Positron emission tomography
PFK1: Phosphofructokinase 1
PGC-1α: Peroxisome proliferator-activated receptor gamma coactivator 1α
PGK1: Phosphoglycerate kinase 1
PHGDH: Phosphoglycerate dehydrogenase
PI3K: Phosphatidylinositol-3-kinase
PKM2: Pyruvate kinase M2
PPAR: Peroxisome proliferator-activated receptor
PPP: Pentose phosphate pathway
PRMT1: Protein arginine methyltransferase 1
PTBP1: Polypyrimidine tract-binding protein 1
PYCR: Pyrroline-5-carboxylate reductase
R-5-P: Ribose-5-phosphate
RIPK3: Receptor-interacting serine/threonine kinase 3
ROS: Reactive oxygen species
SAM: S-adenosylmethionine
SCD1: Stearoyl-CoA desaturase 1
SDH: Succinate dehydrogenase
SEC63: Protein transport protein Sec63
SFA: Saturated fatty acid
SGLT: Sodium-glucose cotransporter
SHMT: Serine hydroxymethyltransferase
SLC: Solute carrier
SOAT1: Sterol O-acyltransferase 1
SREBP: Sterol regulatory element–binding protein
STARD1: Steroidogenic acute regulatory protein D1
STIM1: Stromal interaction molecule 1
TARBP1: TAR RNA binding protein 1
TAZ: Transcriptional coactivator with a PDZ-binding motif
TCA: Tricarboxylic acid
TIGAR: TP53-induced glycolysis and apoptosis regulator
TKT: Transketolase
TME: Tumor microenvironment
TPI1: Triosephosphate isomerase 1
UFA: Unsaturated fatty acid
USP: Ubiquitin-specific peptidase
YAP: Yes-associated protein
ZNF263: Zinc finger protein 263
α-KG: α-Ketoglutarate

## References

[1] Siegel RL, Kratzer TB, Giaquinto AN, Sung H, Jemal A. Cancer statistics, 2025. CA Cancer J Clin. 2025;75(1):10–45. 10.3322/caac.21871. 39817679 10.3322/caac.21871 PMC11745215

[2] Mao Y, Xia Z, Xia W, Jiang P. Metabolic reprogramming, sensing, and cancer therapy. Cell Rep. 2024;43(12):115064. 10.1016/j.celrep.2024.115064. 39671294 10.1016/j.celrep.2024.115064

[3] Du LX, Sheng GL, Shi AD, Li KS, Liu ZL, Tang YC, et al. Prognostic nomogram for patients with advanced unresectable hepatocellular carcinoma treated with TAE combined with HAIC. Front Pharmacol. 2024;15:1426912. 10.3389/fphar.2024.1426912. 39234115 10.3389/fphar.2024.1426912 PMC11371787

[4] Kazi IA, Jahagirdar V, Kabir BW, Syed AK, Kabir AW, Perisetti A. Role of imaging in screening for hepatocellular carcinoma. Cancers (Basel). 2024. 10.3390/cancers16193400. 39410020 10.3390/cancers16193400 PMC11476228

[5] Lu JJ, Yan S, Chen L, Ju LL, Cai WH, Wu JZ. Retrospective analysis of patients with hepatocellular carcinoma complicated with human immunodeficiency virus infection after hepatectomy. World J Gastrointest Oncol. 2024;16(9):3851–64. 10.4251/wjgo.v16.i9.3851. 39350989 10.4251/wjgo.v16.i9.3851 PMC11438767

[6] Elhinnawi MA, Boushra MI, Hussien DM, Hussein FH, Abdelmawgood IA. Mitochondria’s role in the maintenance of cancer stem cells in hepatocellular carcinoma. Stem Cell Rev Rep. 2025;21(1):198–210. 10.1007/s12015-024-10797-1. 39422808 10.1007/s12015-024-10797-1

[7] Attal N, Sullivan MT, Girardi CA, Thompson KJ, McKillop IH. Fatty acid binding protein-4 promotes alcohol-dependent hepatosteatosis and hepatocellular carcinoma progression. Transl Oncol. 2021;14(1):100975. 10.1016/j.tranon.2020.100975. 33290990 10.1016/j.tranon.2020.100975 PMC7719965

[8] Li HY, Jing YM, Shen X, Tang MY, Shen HH, Li XW, et al. Protein tyrosine phosphatase non-receptor II: a possible biomarker of poor prognosis and mediator of immune evasion in hepatocellular carcinoma. World J Gastrointest Oncol. 2024;16(9):3913–31. 10.4251/wjgo.v16.i9.3913. 39350977 10.4251/wjgo.v16.i9.3913 PMC11438766

[9] Gringeri E, Furlanetto A, Billato I, Cescon M, De Carlis L, Mazzaferro V, et al. The Italian experience on liver transplantation for unresectable peri-hilar cholangiocarcinoma: a national survey and future perspectives. Updates Surg. 2024;76(7):2505–13. 10.1007/s13304-024-01889-1. 39210194 10.1007/s13304-024-01889-1

[10] Du J, Bai D, Gu C, Zhao J, Zhou C, Wang Y, et al. Sorafenib-mediated cleavage of p62 initiates cellular senescence as a mechanism to evade its anti-hepatocellular carcinoma efficacy. Oncogene. 2024;43(40):3003–17. 10.1038/s41388-024-03142-w. 39232218 10.1038/s41388-024-03142-w

[11] Park S, Hall MN. Metabolic reprogramming in hepatocellular carcinoma: mechanisms and therapeutic implications. Exp Mol Med. 2025;57(3):515–23. 10.1038/s12276-025-01415-2. 40025169 10.1038/s12276-025-01415-2 PMC11958682

[12] Yang F, Hilakivi-Clarke L, Shaha A, Wang Y, Wang X, Deng Y, et al. Metabolic reprogramming and its clinical implication for liver cancer. Hepatology. 2023;78(5):1602–24. 10.1097/hep.0000000000000005. 36626639 10.1097/HEP.0000000000000005 PMC10315435

[13] Wang H, Lun Y, Xu D, Jiang H, Yan Y, Yang X. Research progress and therapeutic strategies in hepatocellular carcinoma metabolic reprogramming. J Adv Res. 2025. 10.1016/j.jare.2025.08.023.40850678 10.1016/j.jare.2025.08.023

[14] Wang K, Li X, Guo S, Chen J, Lv Y, Guo Z, et al. Metabolic reprogramming of glucose: the metabolic basis for the occurrence and development of hepatocellular carcinoma. Front Oncol. 2025;15:1545086. 10.3389/fonc.2025.1545086. 39980550 10.3389/fonc.2025.1545086 PMC11839411

[15] Wong TL, Kong Y, Ma S. Lipid metabolism in cancer cells: Its role in hepatocellular carcinoma progression and therapeutic resistance. Hepatol Commun. 2025;9(11). 10.1097/hc9.0000000000000837. 10.1097/HC9.0000000000000837 PMC12548999 41118283

[16] Ye Y, Yu B, Wang H, Yi F. Glutamine metabolic reprogramming in hepatocellular carcinoma. Front Mol Biosci. 2023;10:1242059. 10.3389/fmolb.2023.1242059. 37635935 10.3389/fmolb.2023.1242059 PMC10452011

[17] Liao M, Yao D, Wu L, Luo C, Wang Z, Zhang J, et al. Targeting the Warburg effect: a revisited perspective from molecular mechanisms to traditional and innovative therapeutic strategies in cancer. Acta Pharm Sin B. 2024;14(3):953–1008. 10.1016/j.apsb.2023.12.003. 38487001 10.1016/j.apsb.2023.12.003 PMC10935242

[18] Khan F, Elsori D, Verma M, Pandey S, Obaidur Rab S, Siddiqui S, et al. Unraveling the intricate relationship between lipid metabolism and oncogenic signaling pathways. Front Cell Dev Biol. 2024;12:1399065. 10.3389/fcell.2024.1399065. 38933330 10.3389/fcell.2024.1399065 PMC11199418

[19] Tufail M, Jiang CH, Li N. Altered metabolism in cancer: insights into energy pathways and therapeutic targets. Mol Cancer. 2024;23(1):203. 10.1186/s12943-024-02119-3. 39294640 10.1186/s12943-024-02119-3 PMC11409553

[20] Dou Q, Grant AK, de Coutinto Souza P, Moussa M, Nasser I, Ahmed M, et al. Characterizing metabolic heterogeneity of hepatocellular carcinoma with hyperpolarized (13)C pyruvate MRI and mass spectrometry. Radiol Imaging Cancer. 2024;6(2):e230056. 10.1148/rycan.230056. 38426887 10.1148/rycan.230056 PMC10988335

[21] Liu C, Chen G, Liao J, Wu D, Chen X, Wang N. M6A modification-mediated regulation of DCAF13 promotes glycolytic metabolism and drives hepatocellular carcinoma progression via interaction with G6PD. J Gastroenterol Hepatol. 2025;40(9):2311–23. 10.1111/jgh.70015. 40708455 10.1111/jgh.70015

[22] Liu H, Li X, Zhang C, Hao X, Cao Y, Wang Y, et al. GJB2 promotes HCC progression by activating glycolysis through cytoplasmic translocation and generating a suppressive tumor microenvironment based on single cell RNA sequencing. Adv Sci Weinh. 2024;11(39):e2402115. 10.1002/advs.202402115. 39162005 10.1002/advs.202402115 PMC11497106

[23] Nakahara R, Maeda K, Aki S, Osawa T. Metabolic adaptations of cancer in extreme tumor microenvironments. Cancer Sci. 2023;114(4):1200–7. 10.1111/cas.15722. 36630222 10.1111/cas.15722 PMC10067430

[24] Fujimoto T, Teraishi F, Kanehira N, Tajima T, Sakurai Y, Kondo N, et al. Bnct pancreatic cancer treatment strategy with glucose-conjugated boron drug. Biomaterials. 2024;309:122605. 10.1016/j.biomaterials.2024.122605. 38754291 10.1016/j.biomaterials.2024.122605

[25] Nam M, Xia W, Mir AH, Jerrett A, Spinelli JB, Huang TT, et al. Glucose limitation protects cancer cells from apoptosis induced by pyrimidine restriction and replication inhibition. Nat Metab. 2024;6(12):2338–53. 10.1038/s42255-024-01166-w. 39592843 10.1038/s42255-024-01166-w PMC12019718

[26] Wang T, Zhang Y, Liu Y, Huang Y, Wang W. Amino acid-starved cancer cells utilize macropinocytosis and ubiquitin-proteasome system for nutrient acquisition. Adv Sci (Weinh). 2024;11(1):e2304791. 10.1002/advs.202304791. 37983609 10.1002/advs.202304791 PMC10767443

[27] Jiang C, Qian Y, Bai X, Li S, Zhang L, Xie Y, et al. SLC7A5/E2F1/PTBP1/PKM2 axis mediates progression and therapy effect of triple-negative breast cancer through the crosstalk of amino acid metabolism and glycolysis pathway. Cancer Lett. 2025;617:217612. 10.1016/j.canlet.2025.217612. 40054655 10.1016/j.canlet.2025.217612

[28] Zhong S, Chen W, Wang B, Gao C, Liu X, Song Y, et al. Energy stress modulation of AMPK/FoxO3 signaling inhibits mitochondria-associated ferroptosis. Redox Biol. 2023;63:102760. 10.1016/j.redox.2023.102760. 37267686 10.1016/j.redox.2023.102760 PMC10244700

[29] Liu N, Yan M, Tao Q, Wu J, Chen J, Chen X, et al. Inhibition of TCA cycle improves the anti-PD-1 immunotherapy efficacy in melanoma cells via ATF3-mediated PD-L1 expression and glycolysis. J Immunother Cancer. 2023. 10.1136/jitc-2023-007146. 37678921 10.1136/jitc-2023-007146 PMC10496672

[30] Lan T, Arastu S, Lam J, Kim H, Wang W, Wang S, et al. Glucose-6-phosphate dehydrogenase maintains redox homeostasis and biosynthesis in LKB1-deficient KRAS-driven lung cancer. Nat Commun. 2024;15(1):5857. 10.1038/s41467-024-50157-8. 38997257 10.1038/s41467-024-50157-8 PMC11245543

[31] Dong S, Liang S, Cheng Z, Zhang X, Luo L, Li L, et al. ROS/PI3K/Akt and Wnt/β-catenin signalings activate HIF-1α-induced metabolic reprogramming to impart 5-fluorouracil resistance in colorectal cancer. J Exp Clin Cancer Res. 2022;41(1):15. 10.1186/s13046-021-02229-6. 34998404 10.1186/s13046-021-02229-6 PMC8742403

[32] Chen Y, Xu W, Jin H, Zhang M, Liu S, Liu Y, et al. Nutritional glutamine-modified iron-delivery system with enhanced endocytosis for ferroptosis therapy of pancreatic tumors. ACS Nano. 2024;18(46):31846–68. 10.1021/acsnano.4c08083. 39512234 10.1021/acsnano.4c08083

[33] Tharp KM, Kersten K, Maller O, Timblin GA, Stashko C, Canale FP, et al. Tumor-associated macrophages restrict CD8(+) T cell function through collagen deposition and metabolic reprogramming of the breast cancer microenvironment. Nat Cancer. 2024;5(7):1045–62. 10.1038/s43018-024-00775-4. 38831058 10.1038/s43018-024-00775-4 PMC12204312

[34] Lan T, Gao F, Cai Y, Lv Y, Zhu J, Liu H, et al. The protein circPETH-147aa regulates metabolic reprogramming in hepatocellular carcinoma cells to remodel immunosuppressive microenvironment. Nat Commun. 2025;16(1):333. 10.1038/s41467-024-55577-0. 39747873 10.1038/s41467-024-55577-0 PMC11696079

[35] Tang W, Zhou J, Yang W, Feng Y, Wu H, Mok MTS, et al. Aberrant cholesterol metabolic signaling impairs antitumor immunosurveillance through natural killer T cell dysfunction in obese liver. Cell Mol Immunol. 2022;19(7):834–47. 10.1038/s41423-022-00872-3. 35595819 10.1038/s41423-022-00872-3 PMC9243114

[36] Liu P, Huang F, Lin P, Liu J, Zhou P, Wang J, et al. Histidine metabolism drives liver cancer progression via immune microenvironment modulation through metabolic reprogramming. J Transl Med. 2025;23(1):262. 10.1186/s12967-025-06267-y. 40038727 10.1186/s12967-025-06267-y PMC11877819

[37] Ma Z, Yang J, Jia W, Li L, Li Y, Hu J, et al. Histone lactylation-driven B7-H3 expression promotes tumor immune evasion. Theranostics. 2025;15(6):2338–59. 10.7150/thno.105947. 39990209 10.7150/thno.105947 PMC11840737

[38] Sun T, Liu B, Li Y, Wu J, Cao Y, Yang S, et al. Oxamate enhances the efficacy of CAR-T therapy against glioblastoma via suppressing ectonucleotidases and CCR8 lactylation. J Exp Clin Cancer Res. 2023;42(1):253. 10.1186/s13046-023-02815-w. 37770937 10.1186/s13046-023-02815-w PMC10540361

[39] Wu L, Jin Y, Zhao X, Tang K, Zhao Y, Tong L, et al. Tumor aerobic glycolysis confers immune evasion through modulating sensitivity to T cell-mediated bystander killing via TNF-α. Cell Metab. 2023;35(9):1580-96.e9. 10.1016/j.cmet.2023.07.001. 37506695 10.1016/j.cmet.2023.07.001

[40] Chen Y, Yan Q, Ruan S, Cui J, Li Z, Zhang Z, et al. GCLM lactylation mediated by ACAT2 promotes ferroptosis resistance in KRAS(G12D)-mutant cancer. Cell Rep. 2025;44(6):115774. 10.1016/j.celrep.2025.115774. 40503938 10.1016/j.celrep.2025.115774

[41] Gurung S, Budden T, Mallela K, Jenkins B, von Kriegsheim A, Manrique E, et al. Stromal lipid species dictate melanoma metastasis and tropism. Cancer Cell. 2025;43(6):1108-24.e11. 10.1016/j.ccell.2025.04.001. 40280124 10.1016/j.ccell.2025.04.001 PMC7618647

[42] Dahboul F, Sun J, Buchard B, Abeywickrama-Samarakoon N, Pujos-Guillot E, Durand S, et al. Simultaneous activation of beta-oxidation and de novo lipogenesis in MASLD-HCC: a new paradigm. Liver Int. 2025;45(2):e70006. 10.1111/liv.70006. 39840890 10.1111/liv.70006 PMC11752690

[43] Chen F, Tang H, Li C, Kang R, Tang D, Liu J. CYP51A1 drives resistance to pH-dependent cell death in pancreatic cancer. Nat Commun. 2025;16(1):2278. 10.1038/s41467-025-57583-2. 40055353 10.1038/s41467-025-57583-2 PMC11889236

[44] Teng H, Hang Q, Zheng C, Yan Y, Liu S, Zhao Y, et al. In vivo CRISPR activation screen identifies acyl-CoA-binding protein as a driver of bone metastasis. Sci Transl Med. 2025;17(799):eado7225. 10.1126/scitranslmed.ado7225. 40397713 10.1126/scitranslmed.ado7225 PMC12697304

[45] Zeng Y, Zhao L, Zeng K, Zhan Z, Zhan Z, Li S, et al. TRAF3 loss protects glioblastoma cells from lipid peroxidation and immune elimination via dysregulated lipid metabolism. J Clin Invest. 2025. 10.1172/jci178550. 39932808 10.1172/JCI178550 PMC11957706

[46] Luo Y, Zeng Y, Liu Y, Liu S, Dai Y, Pan W, et al. ACSL5 regulated acetyl-CoA to promote bladder cancer cellular senescence via 53BP1 acetylation. Oncogene. 2025;44(34):3096–112. 10.1038/s41388-025-03474-1. 40595416 10.1038/s41388-025-03474-1

[47] Yi Y, Wang G, Zhang W, Yu S, Fei J, An T, et al. Mitochondrial-cytochrome c oxidase II promotes glutaminolysis to sustain tumor cell survival upon glucose deprivation. Nat Commun. 2025;16(1):212. 10.1038/s41467-024-55768-9. 39747079 10.1038/s41467-024-55768-9 PMC11695821

[48] Avram L, Crișan D, Moldovan RC, Bogos LG, Iuga CA, Andraș D, et al. Metabolomic exploration of colorectal cancer through amino acids and acylcarnitines profiling of serum samples. Cancers (Basel). 2025. 10.3390/cancers17030427. 39941796 10.3390/cancers17030427 PMC11816151

[49] Yamamoto T, Hayashida T, Masugi Y, Oshikawa K, Hayakawa N, Itoh M, et al. PRMT1 sustains de novo fatty acid synthesis by methylating PHGDH to drive chemoresistance in triple-negative breast cancer. Cancer Res. 2024;84(7):1065–83. 10.1158/0008-5472.Can-23-2266. 38383964 10.1158/0008-5472.CAN-23-2266 PMC10982647

[50] Lu B, Chen S, Guan X, Chen X, Du Y, Yuan J, et al. Lactate accumulation induces H4K12la to activate super-enhancer-driven RAD23A expression and promote niraparib resistance in ovarian cancer. Mol Cancer. 2025;24(1):83. 10.1186/s12943-025-02295-w. 40102876 10.1186/s12943-025-02295-w PMC11921584

[51] Wei Z, Xia J, Li J, Cai J, Shan J, Zhang C, et al. SIRT1 promotes glucolipid metabolic conversion to facilitate tumor development in colorectal carcinoma. Int J Biol Sci. 2023;19(6):1925–40. 10.7150/ijbs.76704. 37063423 10.7150/ijbs.76704 PMC10092765

[52] Macheda ML, Rogers S, Best JD. Molecular and cellular regulation of glucose transporter (GLUT) proteins in cancer. J Cell Physiol. 2005;202(3):654–62. 10.1002/jcp.20166. 15389572 10.1002/jcp.20166

[53] Ran J, Li F, Zhan L, Jin Y, Dong Q, Li X, et al. Hypoxia regulates glycolysis through the HIF-1α/BMAL1/ALDOC axis to reduce oxaliplatin sensitivity in colorectal cancer. J Cancer. 2025;16(8):2503–15. 10.7150/jca.108582. 40535800 10.7150/jca.108582 PMC12171004

[54] Zamudio-Martínez E, Delgado-Bellido D, Borrego-Pérez J, Garcia-Diaz A, Herrera-Campos AB, Rodríguez-Vargas JM, et al. Tankyrases modulate the hypoxia response through non-catalytic mechanisms affecting HIF-1α. Cell Commun Signal. 2025;23(1):477. 10.1186/s12964-025-02480-w. 41194181 10.1186/s12964-025-02480-w PMC12587615

[55] Hao Z, Wang Y, Li J, Liu W, Zhao W, Wang J. Expression of HIF-1α/PKM2 axis correlates to biological and clinical significance in papillary thyroid carcinoma. Medicine (Baltimore). 2023;102(10):e33232. 10.1097/md.0000000000033232. 36897686 10.1097/MD.0000000000033232 PMC9997831

[56] Yi H, Han Y, Li Q, Lin R, Zhang J, Yang Y, et al. Prognostic impact of the combination of HIF‑1α and GLUT1 in patients with oesophageal squamous cell carcinoma. Oncol Lett. 2023;26(3):404. 10.3892/ol.2023.13990. 37600334 10.3892/ol.2023.13990 PMC10433721

[57] Zhang Q, Qin Y, Sun X, Bian Z, Liu L, Liu H, et al. Sodium butyrate blocks the growth of colorectal cancer by inhibiting the aerobic glycolysis mediated by SIRT4/HIF-1α. Chem Biol Interact. 2024;403:111227. 10.1016/j.cbi.2024.111227. 39241941 10.1016/j.cbi.2024.111227

[58] Yi X, Qi M, Huang M, Zhou S, Xiong J. Honokiol inhibits HIF-1α-mediated glycolysis to halt breast cancer growth. Front Pharmacol. 2022;13:796763. 10.3389/fphar.2022.796763. 35350760 10.3389/fphar.2022.796763 PMC8957822

[59] Wei S, Zhang J, Zhao R, Shi R, An L, Yu Z, et al. Histone lactylation promotes malignant progression by facilitating USP39 expression to target PI3K/AKT/HIF-1α signal pathway in endometrial carcinoma. Cell Death Discov. 2024;10(1):121. 10.1038/s41420-024-01898-4. 38459014 10.1038/s41420-024-01898-4 PMC10923933

[60] Thakor P, Siddiqui MQ, Patel TR. Analysis of the interlink between glucose-6-phosphate dehydrogenase (G6PD) and lung cancer through multi-omics databases. Heliyon. 2024;10(15):e35158. 10.1016/j.heliyon.2024.e35158. 39165939 10.1016/j.heliyon.2024.e35158 PMC11334843

[61] Peñuelas-Haro I, Espinosa-Sotelo R, Crosas-Molist E, Herranz-Itúrbide M, Caballero-Díaz D, Alay A, et al. The NADPH oxidase NOX4 regulates redox and metabolic homeostasis preventing HCC progression. Hepatology. 2023;78(2):416–33. 10.1002/hep.32702. 35920301 10.1002/hep.32702 PMC10344438

[62] Xie H, Tong G, Zhang Y, Liang S, Tang K, Yang Q. PGK1 drives hepatocellular carcinoma metastasis by enhancing metabolic process. Int J Mol Sci. 2017. 10.3390/ijms18081630. 28749413 10.3390/ijms18081630 PMC5578020

[63] Xia P, Zhang H, Lu H, Xu K, Jiang X, Jiang Y, et al. METTL5 stabilizes c-Myc by facilitating USP5 translation to reprogram glucose metabolism and promote hepatocellular carcinoma progression. Cancer Commun (Lond). 2023;43(3):338–64. 10.1002/cac2.12403. 36602428 10.1002/cac2.12403 PMC10009668

[64] Tang YC, Hsiao JR, Jiang SS, Chang JY, Chu PY, Liu KJ, et al. c-MYC-directed NRF2 drives malignant progression of head and neck cancer via glucose-6-phosphate dehydrogenase and transketolase activation. Theranostics. 2021;11(11):5232–47. 10.7150/thno.53417. 33859744 10.7150/thno.53417 PMC8039948

[65] Wu YQ, Zhang CS, Xiong J, Cai DQ, Wang CZ, Wang Y, et al. Low glucose metabolite 3-phosphoglycerate switches PHGDH from serine synthesis to p53 activation to control cell fate. Cell Res. 2023;33(11):835–50. 10.1038/s41422-023-00874-4. 37726403 10.1038/s41422-023-00874-4 PMC10624847

[66] Koyasu S, Horita S, Saito K, Kobayashi M, Ishikita H, Chow CC, et al. ZBTB2 links p53 deficiency to HIF-1-mediated hypoxia signaling to promote cancer aggressiveness. EMBO Rep. 2023;24(1):e54042. 10.15252/embr.202154042. 36341521 10.15252/embr.202154042 PMC9827547

[67] Xia T, Meng L, Xu G, Sun H, Chen H. TRIM33 promotes glycolysis through regulating P53 K48-linked ubiquitination to promote esophageal squamous cell carcinoma growth. Cell Death Dis. 2024;15(10):740. 10.1038/s41419-024-07137-z. 39389957 10.1038/s41419-024-07137-z PMC11467421

[68] Ding R, Wang Y, Xu L, Sang S, Wu G, Yang W, et al. QiDongNing induces lung cancer cell apoptosis via triggering P53/DRP1-mediated mitochondrial fission. J Cell Mol Med. 2024;28(9):e18353. 10.1111/jcmm.18353. 38682742 10.1111/jcmm.18353 PMC11057058

[69] Chen S, Tao Y, Wang Q, Ren J, Jing Y, Huang J, et al. Glucose induced-AKT/mTOR activation accelerates glycolysis and promotes cell survival in acute myeloid leukemia. Leuk Res. 2023;128:107059. 10.1016/j.leukres.2023.107059. 36989577 10.1016/j.leukres.2023.107059

[70] Huang J, Gao W, Liu H, Yin G, Duan H, Huang Z, et al. Up-regulated ANP32E promotes the thyroid carcinoma cell proliferation and migration via activating AKT/mTOR/HK2-mediated glycolysis. Gene. 2020;750:144681. 10.1016/j.gene.2020.144681. 32304784 10.1016/j.gene.2020.144681

[71] Park SY, Chung YS, Park SY, Kim SH. Role of AMPK in regulation of oxaliplatin-resistant human colorectal cancer. Biomedicines. 2022. 10.3390/biomedicines10112690. 36359211 10.3390/biomedicines10112690 PMC9687437

[72] Urrutia AA, Mesa-Ciller C, Guajardo-Grence A, Alkan HF, Soro-Arnáiz I, Vandekeere A, et al. HIF1α-dependent uncoupling of glycolysis suppresses tumor cell proliferation. Cell Rep. 2024;43(4):114103. 10.1016/j.celrep.2024.114103. 38607920 10.1016/j.celrep.2024.114103 PMC11063627

[73] Fujita M, Orisaka M, Mizutani T, Fujita Y, Onuma T, Tsuyoshi H, et al. YAP/TAZ promote GLUT1 expression and are associated with prognosis in endometrial cancer. Cancers (Basel). 2025. 10.3390/cancers17152554. 40805250 10.3390/cancers17152554 PMC12345884

[74] Hanbazazh M, Samman A, Samargandy S, Al-Maghrabi J. Prognostic value of glucose transporter proteins-1 (GLUT1) in breast carcinoma. Libyan J Med. 2023;18(1):2283953. 10.1080/19932820.2023.2283953. 37988377 10.1080/19932820.2023.2283953 PMC11018318

[75] Kim TH, Kwak Y, Song C, Lee HS, Kim DW, Oh HK, et al. GLUT-1 may predict metastases and death in patients with locally advanced rectal cancer. Front Oncol. 2023;13:1094480. 10.3389/fonc.2023.1094480. 36968998 10.3389/fonc.2023.1094480 PMC10036037

[76] Huang H, Xue J, Xie T, Xie ML. Osthole increases the radiosensitivity of hepatoma cells by inhibiting GSK-3β/AMPK/mTOR pathway-controlled glycolysis. Naunyn Schmiedebergs Arch Pharmacol. 2023;396(4):683–92. 10.1007/s00210-022-02347-8. 36445387 10.1007/s00210-022-02347-8

[77] Deng M, Liao S, Deng J, Li C, Liu L, Han Q, et al. S100A2 promotes clear cell renal cell carcinoma tumor metastasis through regulating GLUT2 expression. Cell Death Dis. 2025;16(1):135. 10.1038/s41419-025-07418-1. 40011447 10.1038/s41419-025-07418-1 PMC11865524

[78] Kokilakanit P, Koontongkaew S, Utispan K. Nitric oxide has diverse effects on head and neck cancer cell proliferation and glycolysis. Biomed Rep. 2024;21(1):106. 10.3892/br.2024.1794. 38868526 10.3892/br.2024.1794 PMC11168032

[79] Heo HJ, Park Y, Lee JH, Kim Y, Kim EK, Kim GH, et al. Clinical big-data-based design of GLUT2-targeted carbon nanodots for accurate diagnosis of hepatocellular carcinoma. Nanoscale. 2022;14(45):17053–64. 10.1039/d2nr04238j. 36367284 10.1039/d2nr04238j

[80] Lei Y, Hu Q, Gu J. Expressions of carbohydrate response element binding protein and glucose transporters in liver cancer and clinical significance. Pathol Oncol Res. 2020;26(2):1331–40. 10.1007/s12253-019-00708-y. 31407220 10.1007/s12253-019-00708-y PMC7242283

[81] Kim Y, Yeuni Y, Heo HJ, Kim ES, Myung K, Baryawno N, et al. Solute carrier family 2 member 2 (glucose transporter 2): a common factor of hepatocyte and hepatocellular carcinoma differentiation. PLoS ONE. 2025;20(4):e0321020. 10.1371/journal.pone.0321020. 40279337 10.1371/journal.pone.0321020 PMC12026939

[82] Iwai S, Motono N, Oyama T, Shioya A, Yamada S, Uramoto H. The clinical relevance of the expression of SGLT2 in lung adenocarcinoma. Oncology. 2024;102(8):710–9. 10.1159/000536060. 38232717 10.1159/000536060

[83] Ding L, Chen X, Zhang W, Dai X, Guo H, Pan X, et al. Canagliflozin primes antitumor immunity by triggering PD-L1 degradation in endocytic recycling. J Clin Invest. 2023. 10.1172/jci154754. 36594471 10.1172/JCI154754 PMC9797339

[84] Zhou J, Zhu J, Yu SJ, Ma HL, Chen J, Ding XF, et al. Sodium-glucose co-transporter-2 (SGLT-2) inhibition reduces glucose uptake to induce breast cancer cell growth arrest through AMPK/mTOR pathway. Biomed Pharmacother. 2020. 10.1016/j.biopha.2020.110821. 33068934 10.1016/j.biopha.2020.110821

[85] Kaji K, Nishimura N, Seki K, Sato S, Saikawa S, Nakanishi K, et al. Sodium glucose cotransporter 2 inhibitor canagliflozin attenuates liver cancer cell growth and angiogenic activity by inhibiting glucose uptake. Int J Cancer. 2018;142(8):1712–22. 10.1002/ijc.31193. 29205334 10.1002/ijc.31193

[86] Mao X, Zhang X, Kam L, Chien N, Lai R, Cheung KS, et al. Synergistic association of sodium-glucose cotransporter-2 inhibitor and metformin on liver and non-liver complications in patients with type 2 diabetes mellitus and metabolic dysfunction-associated steatotic liver disease. Gut. 2024;73(12):2054–61. 10.1136/gutjnl-2024-332481. 39122360 10.1136/gutjnl-2024-332481

[87] Nath S, Balling R. The Warburg effect reinterpreted 100 yr on: a first-principles stoichiometric analysis and interpretation from the perspective of ATP metabolism in cancer cells. Function. 2024;5(3):zqae008. 10.1093/function/zqae008. 38706962 10.1093/function/zqae008 PMC11065116

[88] DeBerardinis RJ, Chandel NS. We need to talk about the Warburg effect. Nat Metab. 2020;2(2):127–9. 10.1038/s42255-020-0172-2. 32694689 10.1038/s42255-020-0172-2

[89] Zhang Y, Wu Y, Cheng A, Cui F, Yang Z, Guo J. The lactylation of glucose-6-phosphate dehydrogenase promotes malignant phenotypes in cancer cell lines. Mol Biol Rep. 2025;52(1):861. 10.1007/s11033-025-10960-y. 40900394 10.1007/s11033-025-10960-y

[90] Sowers ML, Herring J, Zhang W, Tang H, Ou Y, Gu W, et al. Analysis of glucose-derived amino acids involved in one-carbon and cancer metabolism by stable-isotope tracing gas chromatography mass spectrometry. Anal Biochem. 2019;566:1–9. 10.1016/j.ab.2018.10.026. 30409761 10.1016/j.ab.2018.10.026 PMC6814379

[91] Yang J, Tong X, Wang W, Yu X, Xu J, Shi S. Targeting CA9 restricts pancreatic cancer progression through pH regulation and ROS production. Cell Oncol (Dordr). 2024;47(6):2367–82. 10.1007/s13402-024-01022-9. 39656421 10.1007/s13402-024-01022-9 PMC12974058

[92] Steeghs J, Offermans K, Jenniskens JCA, Samarska I, Fazzi GE, van den Brandt PA, et al. Relationship between the Warburg effect in tumour cells and the tumour microenvironment in colorectal cancer patients: results from a large multicentre study. Pathol Res Pract. 2023;247:154518. 10.1016/j.prp.2023.154518. 37209573 10.1016/j.prp.2023.154518

[93] Zhang H, Zhai X, Liu Y, Xia Z, Xia T, Du G, et al. NOP2-mediated m5C modification of c-Myc in an EIF3A-dependent manner to reprogram glucose metabolism and promote hepatocellular carcinoma progression. Research Wash D C. 2023;6:0184. 10.34133/research.0184. 37398932 10.34133/research.0184 PMC10313139

[94] Zhang H, Su X, Burley SK, Zheng XFS. mTOR regulates aerobic glycolysis through NEAT1 and nuclear paraspeckle-mediated mechanism in hepatocellular carcinoma. Theranostics. 2022;12(7):3518–33. 10.7150/thno.72581. 35547764 10.7150/thno.72581 PMC9065186

[95] Luo X, Zheng E, Wei L, Zeng H, Qin H, Zhang X, et al. The fatty acid receptor CD36 promotes HCC progression through activating Src/PI3K/AKT axis-dependent aerobic glycolysis. Cell Death Dis. 2021;12(4):328. 10.1038/s41419-021-03596-w. 33771982 10.1038/s41419-021-03596-w PMC7997878

[96] Bergamini C, Leoni I, Rizzardi N, Melli M, Galvani G, Coada CA, et al. MiR-494 induces metabolic changes through G6pc targeting and modulates sorafenib response in hepatocellular carcinoma. J Exp Clin Cancer Res. 2023;42(1):145. 10.1186/s13046-023-02718-w. 37301960 10.1186/s13046-023-02718-w PMC10257313

[97] Wu Y, Song L, Kong J, Wen Q, Jiao J, Wang X, et al. Scribble promotes fibrosis-dependent mechanisms of hepatocarcinogenesis by p53/PUMA-mediated glycolysis. Biochim Biophys Acta Mol Basis Dis. 2023;1869(8):166823. 10.1016/j.bbadis.2023.166823. 37632981 10.1016/j.bbadis.2023.166823

[98] Wang Y, Mang X, Li D, Chen Y, Cai Z, Tan F. Piezoeletric cold atmospheric plasma induces apoptosis and autophagy in human hepatocellular carcinoma cells through blocking glycolysis and AKT/mTOR/HIF-1α pathway. Free Radic Biol Med. 2023;208:134–52. 10.1016/j.freeradbiomed.2023.07.036. 37543168 10.1016/j.freeradbiomed.2023.07.036

[99] Zhang Y, Zhang C, Zhao Q, Wei W, Dong Z, Shao L, et al. The miR-873/NDFIP1 axis promotes hepatocellular carcinoma growth and metastasis through the AKT/mTOR-mediated Warburg effect. Am J Cancer Res. 2019;9(5):927–44. 31218102 PMC6556606

[100] Li X, Zhang CC, Lin XT, Zhang J, Zhang YJ, Yu HQ, et al. Elevated expression of WSB2 degrades p53 and activates the IGFBP3-AKT-mTOR-dependent pathway to drive hepatocellular carcinoma. Exp Mol Med. 2024;56(1):177–91. 10.1038/s12276-023-01142-6. 38177295 10.1038/s12276-023-01142-6 PMC10834962

[101] Huang Q, Li J, Xing J, Li W, Li H, Ke X, et al. CD147 promotes reprogramming of glucose metabolism and cell proliferation in HCC cells by inhibiting the p53-dependent signaling pathway. J Hepatol. 2014;61(4):859–66. 10.1016/j.jhep.2014.04.035. 24801417 10.1016/j.jhep.2014.04.035

[102] Yao G, Yin J, Wang Q, Dong R, Lu J. Glypican-3 enhances reprogramming of glucose metabolism in liver cancer cells. Biomed Res Int. 2019;2019:2560650. 10.1155/2019/2560650. 31781603 10.1155/2019/2560650 PMC6875211

[103] Sun J, Li J, Guo Z, Sun L, Juan C, Zhou Y, et al. Overexpression of pyruvate dehydrogenase E1α subunit inhibits Warburg effect and induces cell apoptosis through mitochondria-mediated pathway in hepatocellular carcinoma. Oncol Res. 2019;27(4):407–14. 10.3727/096504018x15180451872087. 29444744 10.3727/096504018X15180451872087 PMC7848459

[104] Xu IM, Lai RK, Lin SH, Tse AP, Chiu DK, Koh HY, et al. Transketolase counteracts oxidative stress to drive cancer development. Proc Natl Acad Sci U S A. 2016;113(6):E725–34. 10.1073/pnas.1508779113. 26811478 10.1073/pnas.1508779113 PMC4760787

[105] Hu J, Liu N, Song D, Steer CJ, Zheng G, Song G. A positive feedback between cholesterol synthesis and the pentose phosphate pathway rather than glycolysis promotes hepatocellular carcinoma. Oncogene. 2023;42(39):2892–904. 10.1038/s41388-023-02757-9. 37596320 10.1038/s41388-023-02757-9 PMC10516751

[106] Jin X, Lv Y, Bie F, Duan J, Ma C, Dai M, et al. METTL3 confers oxaliplatin resistance through the activation of G6PD-enhanced pentose phosphate pathway in hepatocellular carcinoma. Cell Death Differ. 2025;32(3):466–79. 10.1038/s41418-024-01406-2. 39472692 10.1038/s41418-024-01406-2 PMC11894169

[107] Chen H, Wu D, Bao L, Yin T, Lei D, Yu J, et al. 6PGD inhibition sensitizes hepatocellular carcinoma to chemotherapy via AMPK activation and metabolic reprogramming. Biomed Pharmacother. 2019;111:1353–8. 10.1016/j.biopha.2019.01.028. 30841449 10.1016/j.biopha.2019.01.028

[108] Yin X, Tang B, Li JH, Wang Y, Zhang L, Xie XY, et al. ID1 promotes hepatocellular carcinoma proliferation and confers chemoresistance to oxaliplatin by activating pentose phosphate pathway. J Exp Clin Cancer Res. 2017;36(1):166. 10.1186/s13046-017-0637-7. 29169374 10.1186/s13046-017-0637-7 PMC5701377

[109] Yang C, Cui XW, Ding ZW, Jiang TY, Feng XF, Pan YF, et al. Gankyrin and TIGAR cooperatively accelerate glucose metabolism toward the PPP and TCA cycle in hepatocellular carcinoma. Cancer Sci. 2022;113(12):4151–64. 10.1111/cas.15593. 36114745 10.1111/cas.15593 PMC9746032

[110] Toshida K, Itoh S, Iseda N, Tanaka S, Nakazono K, Tomiyama T, et al. The Impact of TP53-Induced Glycolysis and Apoptosis Regulator on Prognosis in Hepatocellular Carcinoma: Association with Tumor Microenvironment and Ferroptosis. Liver Cancer. 2025;14(1):36–57. 10.1159/000540180. 40144470 10.1159/000540180 PMC11936447

[111] Li Y, Chen H, Xie X, Yang B, Wang X, Zhang J, et al. PINK1-mediated mitophagy promotes oxidative phosphorylation and redox homeostasis to induce drug-tolerant persister cancer cells. Cancer Res. 2023;83(3):398–413. 10.1158/0008-5472.Can-22-2370. 36480196 10.1158/0008-5472.CAN-22-2370

[112] Liu YM, Ge JY, Chen YF, Liu T, Chen L, Liu CC, et al. Combined single-cell and spatial transcriptomics reveal the metabolic evolvement of breast cancer during early dissemination. Adv Sci (Weinh). 2023;10(6):e2205395. 10.1002/advs.202205395. 36594618 10.1002/advs.202205395 PMC9951304

[113] Liu Y, Tian W, Ge C, Zhang C, Huang Z, Zhang C, et al. SNX17 mediates STAT3 activation to promote hepatocellular carcinoma progression via a retromer dependent mechanism. Int J Biol Sci. 2025;21(6):2762–79. 10.7150/ijbs.110506. 40303303 10.7150/ijbs.110506 PMC12035908

[114] Ji X, Yang Z, Li C, Zhu S, Zhang Y, Xue F, et al. Mitochondrial ribosomal protein L12 potentiates hepatocellular carcinoma by regulating mitochondrial biogenesis and metabolic reprogramming. Metabolism. 2024;152:155761. 10.1016/j.metabol.2023.155761. 38104924 10.1016/j.metabol.2023.155761

[115] Yang T, Liang N, Zhang J, Bai Y, Li Y, Zhao Z, et al. OCTN2 enhances PGC-1α-mediated fatty acid oxidation and OXPHOS to support stemness in hepatocellular carcinoma. Metabolism. 2023;147:155628. 10.1016/j.metabol.2023.155628. 37315888 10.1016/j.metabol.2023.155628

[116] Yuan T, Zhou T, Qian M, Du J, Liu Y, Wang J, et al. SDHA/B reduction promotes hepatocellular carcinoma by facilitating the deNEDDylation of cullin1 and stabilizing YAP/TAZ. Hepatology. 2023;78(1):103–19. 10.1002/hep.32621. 35713976 10.1002/hep.32621

[117] Li J, Liang N, Long X, Zhao J, Yang J, Du X, et al. SDHC-related deficiency of SDH complex activity promotes growth and metastasis of hepatocellular carcinoma via ROS/NFκB signaling. Cancer Lett. 2019;461:44–55. 10.1016/j.canlet.2019.07.001. 31278950 10.1016/j.canlet.2019.07.001

[118] Tseng PL, Wu WH, Hu TH, Chen CW, Cheng HC, Li CF, et al. Decreased succinate dehydrogenase B in human hepatocellular carcinoma accelerates tumor malignancy by inducing the Warburg effect. Sci Rep. 2018;8(1):3081. 10.1038/s41598-018-21361-6. 29449614 10.1038/s41598-018-21361-6 PMC5814459

[119] Li X, Du Y, Jiang W, Dong S, Li W, Tang H, et al. Integrated transcriptomics, proteomics and metabolomics-based analysis uncover TAM2-associated glycolysis and pyruvate metabolic remodeling in pancreatic cancer. Front Immunol. 2023;14:1170223. 10.3389/fimmu.2023.1170223. 37662928 10.3389/fimmu.2023.1170223 PMC10470650

[120] Tang X, Xue J, Zhang J, Zhou J. A gluconeogenesis-related genes model for predicting prognosis, tumor microenvironment infiltration, and drug sensitivity in hepatocellular carcinoma. J Hepatocell Carcinoma. 2024;11:1907–26. 10.2147/jhc.S483664. 39386981 10.2147/JHC.S483664 PMC11463187

[121] Liu MX, Jin L, Sun SJ, Liu P, Feng X, Cheng ZL, et al. Metabolic reprogramming by PCK1 promotes TCA cataplerosis, oxidative stress and apoptosis in liver cancer cells and suppresses hepatocellular carcinoma. Oncogene. 2018;37(12):1637–53. 10.1038/s41388-017-0070-6. 29335519 10.1038/s41388-017-0070-6 PMC5860930

[122] Xiang J, Chen C, Liu R, Gou D, Chang L, Deng H, et al. Gluconeogenic enzyme PCK1 deficiency promotes CHK2 O-GlcNAcylation and hepatocellular carcinoma growth upon glucose deprivation. J Clin Invest. 2021. 10.1172/jci144703. 33690219 10.1172/JCI144703 PMC8262473

[123] Tanaka K, Tsuji K, Hiraoka A, Tada T, Hirooka M, Kariyama K, et al. Clinical Utility of a Prognostic Scoring System Based on LDH and CRP in HCC Patients Receiving Atezolizumab Plus Bevacizumab. Liver Int. 2025;45(10):e70286. 10.1111/liv.70286. 40970650 10.1111/liv.70286

[124] Fang Y, Liu W, Tang Z, Ji X, Zhou Y, Song S, et al. Monocarboxylate transporter 4 inhibition potentiates hepatocellular carcinoma immunotherapy through enhancing T cell infiltration and immune attack. Hepatology. 2023;77(1):109–23. 10.1002/hep.32348. 35043976 10.1002/hep.32348

[125] Yang Z, Yan C, Ma J, Peng P, Ren X, Cai S, et al. Lactylome analysis suggests lactylation-dependent mechanisms of metabolic adaptation in hepatocellular carcinoma. Nat Metab. 2023;5(1):61–79. 10.1038/s42255-022-00710-w. 36593272 10.1038/s42255-022-00710-w

[126] Zhao Y, Li M, Yao X, Fei Y, Lin Z, Li Z, et al. HCAR1/MCT1 regulates tumor ferroptosis through the lactate-mediated AMPK-SCD1 activity and its therapeutic implications. Cell Rep. 2020;33(10):108487. 10.1016/j.celrep.2020.108487. 33296645 10.1016/j.celrep.2020.108487

[127] Cho S, Chun Y, He L, Ramirez CB, Ganesh KS, Jeong K, et al. FAM120A couples SREBP-dependent transcription and splicing of lipogenesis enzymes downstream of mTORC1. Mol Cell. 2023;83(16):3010-26.e8. 10.1016/j.molcel.2023.07.017. 37595559 10.1016/j.molcel.2023.07.017 PMC10494788

[128] Liu Y, Wang F, Yan G, Tong Y, Guo W, Li S, et al. CPT1A loss disrupts BCAA metabolism to confer therapeutic vulnerability in TP53-mutated liver cancer. Cancer Lett. 2024;595:217006. 10.1016/j.canlet.2024.217006. 38823763 10.1016/j.canlet.2024.217006

[129] Li B, Cheng B, Huang H, Huang S, Yu S, Li Z, et al. Darolutamide-mediated phospholipid remodeling induces ferroptosis through the SREBP1-FASN axis in prostate cancer. Int J Biol Sci. 2024;20(12):4635–53. 10.7150/ijbs.101039. 39309439 10.7150/ijbs.101039 PMC11414384

[130] Chen M, Li H, Zheng S, Shen J, Chen Y, Li Y, et al. Nobiletin targets SREBP1/ACLY to induce autophagy-dependent cell death of gastric cancer cells through PI3K/Akt/mTOR signaling pathway. Phytomedicine. 2024;128:155360. 10.1016/j.phymed.2024.155360. 38547624 10.1016/j.phymed.2024.155360

[131] Liu C, Liu J, Liu G, Song Y, Yang X, Gao H, et al. Anthocyanins and flavonoids derived from *Clitoria ternatea* L. flower inhibit bladder cancer growth via suppressing fatty acid synthesis mediated by SREBP1 pathway. Acta Biochim Biophys Sin. 2024;57(5):770–81. 10.3724/abbs.2024192. 39468929 10.3724/abbs.2024192 PMC12130705

[132] Wang S, Zhou Y, Yu R, Ling J, Li B, Yang C, et al. Loss of hepatic FTCD promotes lipid accumulation and hepatocarcinogenesis by upregulating PPARγ and SREBP2. JHEP Rep. 2023;5(10):100843. 10.1016/j.jhepr.2023.100843. 37675273 10.1016/j.jhepr.2023.100843 PMC10477690

[133] Zhu Z, Jiang W, Zhou J, Maldeney AR, Liang J, Yang J, et al. The combined inhibition of SREBP and mTORC1 signaling synergistically inhibits B-cell lymphoma. Cancer Med. 2024;13(21):e70342. 10.1002/cam4.70342. 39501600 10.1002/cam4.70342 PMC11538279

[134] Liang JH, Wang WT, Wang R, Gao R, Du KX, Duan ZW, et al. PRMT5 activates lipid metabolic reprogramming via MYC contributing to the growth and survival of mantle cell lymphoma. Cancer Lett. 2024;591:216877. 10.1016/j.canlet.2024.216877. 38615930 10.1016/j.canlet.2024.216877

[135] Morelli E, Ribeiro CF, Rodrigues SD, Gao C, Socciarelli F, Maisano D, et al. Targeting acetyl-CoA carboxylase suppresses de novo lipogenesis and tumor cell growth in multiple myeloma. Clin Cancer Res. 2025;31(10):1975–87. 10.1158/1078-0432.Ccr-24-2000. 40053701 10.1158/1078-0432.CCR-24-2000 PMC12081190

[136] Singh KB, Hahm ER, Kim SH, Wendell SG, Singh SV. A novel metabolic function of Myc in regulation of fatty acid synthesis in prostate cancer. Oncogene. 2021;40(3):592–602. 10.1038/s41388-020-01553-z. 33199826 10.1038/s41388-020-01553-z PMC7855479

[137] Zhu K, Ma S, Chen H, Xie J, Huang D, Fu C, et al. Value of Laboratory Indicators in Predicting Pneumonia in Symptomatic COVID-19 Patients Infected with the SARS-CoV-2 Omicron Variant. Infect Drug Resist. 2023;16:1159–70. 10.2147/idr.S397231. 36879854 10.2147/IDR.S397231 PMC9985399

[138] Lorenz NI, Sauer B, Urban H, Weinem JB, Parmar BS, Zeiner PS, et al. AMP-activated protein kinase mediates adaptation of glioblastoma cells to conditions of the tumor microenvironment. J Exp Clin Cancer Res. 2025;44(1):104. 10.1186/s13046-025-03346-2. 40122814 10.1186/s13046-025-03346-2 PMC11931870

[139] Luo J, Pan S, Luo J, Wang L, Yin J, Zhao H, et al. *GNA15* induces drug resistance in B cell acute lymphoblastic leukemia by promoting fatty acid oxidation via activation of the AMPK pathway. Mol Cell Biochem. 2025;480(6):3719–33. 10.1007/s11010-024-05198-4. 39812998 10.1007/s11010-024-05198-4 PMC12095422

[140] Lu L, Hu X, Han Y, Wang H, Tian Z, Zhang Y, et al. ENPP2 promotes progression and lipid accumulation via AMPK/SREBP1/FAS pathway in chronic lymphocytic leukemia. Cell Mol Biol Lett. 2024;29(1):159. 10.1186/s11658-024-00675-6. 39731014 10.1186/s11658-024-00675-6 PMC11681649

[141] Yang F, Li Y, Shang X, Zhu Y, Hou W, Liu Y, et al. PLIN2 promotes colorectal cancer progression through CD36-mediated epithelial-mesenchymal transition. Cell Death Dis. 2025;16(1):510. 10.1038/s41419-025-07836-1. 40640171 10.1038/s41419-025-07836-1 PMC12246428

[142] Yang Y, Liu X, Yang D, Li L, Li S, Lu S, et al. Interplay of *CD36*, autophagy, and lipid metabolism: insights into cancer progression. Metabolism. 2024;155:155905. 10.1016/j.metabol.2024.155905. 38548128 10.1016/j.metabol.2024.155905

[143] Peche VS, Pietka TA, Jacome-Sosa M, Samovski D, Palacios H, Chatterjee-Basu G, et al. Endothelial cell *CD36* regulates membrane ceramide formation, exosome fatty acid transfer and circulating fatty acid levels. Nat Commun. 2023;14(1):4029. 10.1038/s41467-023-39752-3. 37419919 10.1038/s41467-023-39752-3 PMC10329018

[144] Tian Y, Yang B, Qiu W, Hao Y, Zhang Z, Yang B, et al. ER-residential Nogo-B accelerates NAFLD-associated HCC mediated by metabolic reprogramming of oxLDL lipophagy. Nat Commun. 2019;10(1):3391. 10.1038/s41467-019-11274-x. 31358770 10.1038/s41467-019-11274-x PMC6662851

[145] Tao L, Ding X, Yan L, Xu G, Zhang P, Ji A, et al. *CD36* accelerates the progression of hepatocellular carcinoma by promoting FAs absorption. Med Oncol. 2022;39(12):202. 10.1007/s12032-022-01808-7. 36175596 10.1007/s12032-022-01808-7

[146] Xu Q, Liao Z, Gong Z, Liu X, Yang Y, Wang Z, et al. Down-regulation of *EVA1A* by miR-103a-3p promotes hepatocellular carcinoma cells proliferation and migration. Cell Mol Biol Lett. 2022;27(1):93. 10.1186/s11658-022-00388-8. 36273122 10.1186/s11658-022-00388-8 PMC9588234

[147] Yang D, Li L, Zang K, Ma W, Yang Y, Sun Y, et al. EVA1A regulates hepatic lipid homeostasis by modulating CD36 expression and its palmitoylation. Research Wash D C. 2025;8:1001. 10.34133/research.1001. 41306774 10.34133/research.1001 PMC12645495

[148] Hong J, Liu J, Zhang Y, Ding L, Ye Q. MiR-3180 inhibits hepatocellular carcinoma growth and metastasis by targeting lipid synthesis and uptake. Cancer Cell Int. 2023;23(1):66. 10.1186/s12935-023-02915-9. 37041584 10.1186/s12935-023-02915-9 PMC10091558

[149] Deng T, Zhao J, Tong Y, Chen Z, He B, Li J, et al. Crosstalk between endothelial progenitor cells and HCC through periostin/CCL2/CD36 supports formation of the pro-metastatic microenvironment in HCC. Oncogene. 2024;43(13):944–61. 10.1038/s41388-024-02960-2. 38351345 10.1038/s41388-024-02960-2

[150] Guo W, Li Z, Anagnostopoulos G, Kong WT, Zhang S, Chakarov S, et al. Notch signaling regulates macrophage-mediated inflammation in metabolic dysfunction-associated steatotic liver disease. Immunity. 2024;57(10):2310-27.e6. 10.1016/j.immuni.2024.08.016. 39317200 10.1016/j.immuni.2024.08.016

[151] Zhu GQ, Tang Z, Huang R, Qu WF, Fang Y, Yang R, et al. CD36(+) cancer-associated fibroblasts provide immunosuppressive microenvironment for hepatocellular carcinoma via secretion of macrophage migration inhibitory factor. Cell Discov. 2023;9(1):25. 10.1038/s41421-023-00529-z. 36878933 10.1038/s41421-023-00529-z PMC9988869

[152] Wang H, Liu F, Wu X, Zhu G, Tang Z, Qu W, et al. Cancer-associated fibroblasts contributed to hepatocellular carcinoma recurrence and metastasis via CD36-mediated fatty-acid metabolic reprogramming. Exp Cell Res. 2024;435(2):113947. 10.1016/j.yexcr.2024.113947. 38301989 10.1016/j.yexcr.2024.113947

[153] Berndt N, Eckstein J, Heucke N, Gajowski R, Stockmann M, Meierhofer D, et al. Characterization of lipid and lipid droplet metabolism in human HCC. Cells. 2019. 10.3390/cells8050512. 31137921 10.3390/cells8050512 PMC6562484

[154] Li L, Zhang X, Xu G, Xue R, Li S, Wu S, et al. Transcriptional Regulation of De Novo Lipogenesis by SIX1 in Liver Cancer Cells. Adv Sci (Weinh). 2024;11(41):e2404229. 10.1002/advs.202404229. 39258807 10.1002/advs.202404229 PMC11538671

[155] Tamarindo GH, Ribeiro CF, Rodrigues S, Góes RM, Loda M. DHA suppresses hormone-sensitive and castration-resistant prostate cancer growth by decreasing de novo lipogenesis. Biochim Biophys Acta Mol Cell Biol Lipids. 2025;1870(5):159634. 10.1016/j.bbalip.2025.159634.40383250 10.1016/j.bbalip.2025.159634

[156] Liu HH, Xu Y, Li CJ, Hsu SJ, Lin XH, Zhang R, et al. An SCD1-dependent mechanoresponsive pathway promotes HCC invasion and metastasis through lipid metabolic reprogramming. Mol Ther. 2022;30(7):2554–67. 10.1016/j.ymthe.2022.03.015. 35358687 10.1016/j.ymthe.2022.03.015 PMC9263248

[157] Ni J, Zhang L, Feng G, Bao W, Wang Y, Huang Y, et al. Vanillic acid restores homeostasis of intestinal epithelium in colitis through inhibiting CA9/STIM1-mediated ferroptosis. Pharmacol Res. 2024;202:107128. 10.1016/j.phrs.2024.107128. 38438089 10.1016/j.phrs.2024.107128

[158] Huang G, Cai Y, Ren M, Zhang X, Fu Y, Cheng R, et al. Salidroside sensitizes Triple-negative breast cancer to ferroptosis by SCD1-mediated lipogenesis and NCOA4-mediated ferritinophagy. J Adv Res. 2025;74:589–607. 10.1016/j.jare.2024.09.027. 39353532 10.1016/j.jare.2024.09.027 PMC12302663

[159] Zhou X, Cui G, Hu E, Wang X, Tang D, Zhang X, et al. The impact of de novo lipogenesis on predicting survival and clinical therapy: an exploration based on a multigene prognostic model in hepatocellular carcinoma. J Transl Med. 2025;23(1):679. 10.1186/s12967-025-06704-y. 40533802 10.1186/s12967-025-06704-y PMC12178006

[160] Tu DY, Peng R, Jin SJ, Su BB, Fan SS, Zhang JH, et al. MARCH8 suppresses hepatocellular carcinoma by promoting SREBP1 degradation and modulating fatty acid de novo synthesis. Cell Death Dis. 2025;16(1):391. 10.1038/s41419-025-07707-9. 40379644 10.1038/s41419-025-07707-9 PMC12084374

[161] Chen J, Ding C, Chen Y, Hu W, Yu C, Peng C, et al. ACSL4 reprograms fatty acid metabolism in hepatocellular carcinoma via c-Myc/SREBP1 pathway. Cancer Lett. 2021;502:154–65. 10.1016/j.canlet.2020.12.019. 33340617 10.1016/j.canlet.2020.12.019

[162] Li J, Huang Q, Long X, Zhang J, Huang X, Aa J, et al. CD147 reprograms fatty acid metabolism in hepatocellular carcinoma cells through Akt/mTOR/SREBP1c and P38/PPARα pathways. J Hepatol. 2015;63(6):1378–89. 10.1016/j.jhep.2015.07.039. 26282231 10.1016/j.jhep.2015.07.039

[163] He XL, Guo HJ, Lei YR, Li J, Li JY, Li MH, et al. NAMPT promotes the malignant progression of HBV-associated hepatocellular carcinoma through activation of SREBP1-mediated lipogenesis. FASEB J. 2024;38(2):e23444. 10.1096/fj.202300070RRR. 38252081 10.1096/fj.202300070RRR

[164] Zhang L, Li XM, Shi XH, Ye K, Fu XL, Wang X, et al. Sorafenib triggers ferroptosis via inhibition of HBXIP/SCD axis in hepatocellular carcinoma. Acta Pharmacol Sin. 2023;44(3):622–34. 10.1038/s41401-022-00981-9. 36109580 10.1038/s41401-022-00981-9 PMC9958095

[165] Hu C, Xin Z, Sun X, Hu Y, Zhang C, Yan R, et al. Activation of ACLY by SEC63 deploys metabolic reprogramming to facilitate hepatocellular carcinoma metastasis upon endoplasmic reticulum stress. J Exp Clin Cancer Res. 2023;42(1):108. 10.1186/s13046-023-02656-7. 37122003 10.1186/s13046-023-02656-7 PMC10150531

[166] Yu L, Liebenberg K, Shen Y, Liu F, Xu Z, Hao X, et al. Tumor-derived arachidonic acid reprograms neutrophils to promote immune suppression and therapy resistance in triple-negative breast cancer. Immunity. 2025;58(4):909-25.e7. 10.1016/j.immuni.2025.03.002. 40157359 10.1016/j.immuni.2025.03.002 PMC11981829

[167] Lee H, Horbath A, Kondiparthi L, Meena JK, Lei G, Dasgupta S, et al. Cell cycle arrest induces lipid droplet formation and confers ferroptosis resistance. Nat Commun. 2024;15(1):79. 10.1038/s41467-023-44412-7. 38167301 10.1038/s41467-023-44412-7 PMC10761718

[168] Wu C, Dai C, Li X, Sun M, Chu H, Xuan Q, et al. AKR1C3-dependent lipid droplet formation confers hepatocellular carcinoma cell adaptability to targeted therapy. Theranostics. 2022;12(18):7681–98. 10.7150/thno.74974. 36451864 10.7150/thno.74974 PMC9706585

[169] Li S, Hao L, Deng J, Zhang J, Hu X. Coptidis rhizoma and evodiae fructus against lipid droplet deposition in nonalcoholic fatty liver disease-related liver cancer by AKT. Chem Biol Drug Des. 2023;102(4):828–42. 10.1111/cbdd.14295. 37460115 10.1111/cbdd.14295

[170] Matsufuji S, Kitajima Y, Higure K, Kimura N, Maeda S, Yamada K, et al. A HIF-1α inhibitor combined with palmitic acid and L-carnitine treatment can prevent the fat metabolic reprogramming under hypoxia and induce apoptosis in hepatocellular carcinoma cells. Cancer Metab. 2023;11(1):25. 10.1186/s40170-023-00328-w. 38066600 10.1186/s40170-023-00328-w PMC10709876

[171] Huang D, Li T, Li X, Zhang L, Sun L, He X, et al. HIF-1-mediated suppression of acyl-CoA dehydrogenases and fatty acid oxidation is critical for cancer progression. Cell Rep. 2014;8(6):1930–42. 10.1016/j.celrep.2014.08.028. 25242319 10.1016/j.celrep.2014.08.028

[172] Liao D, Zeng S, Li C, Yao Y, Guo M, Cui Y, et al. FOXK2 regulates fatty acid metabolism and promotes cervical cancer progression by activating the mTOR/DRP1 signaling axis. Front Cell Dev Biol. 2025;13:1615454. 10.3389/fcell.2025.1615454. 40641601 10.3389/fcell.2025.1615454 PMC12240978

[173] Feng X, Ji Z, Fan X, Kong Y, Yu Y, Shao Y, et al. ASS1 enhances anoikis resistance via AMPK/CPT1A-mediated fatty acid metabolism in ovarian cancer. Cancer Lett. 2024:217082. 10.1016/j.canlet.2024.217082. 10.1016/j.canlet.2024.217082 38914306

[174] Pandey S, Anang V, Schumacher MM. Tumor microenvironment induced switch to mitochondrial metabolism promotes suppressive functions in immune cells. Int Rev Cell Mol Biol. 2024;389:67–103. 10.1016/bs.ircmb.2024.07.003. 39396850 10.1016/bs.ircmb.2024.07.003

[175] Senni N, Savall M, Cabrerizo Granados D, Alves-Guerra MC, Sartor C, Lagoutte I, et al. β-catenin-activated hepatocellular carcinomas are addicted to fatty acids. Gut. 2019;68(2):322–34. 10.1136/gutjnl-2017-315448. 29650531 10.1136/gutjnl-2017-315448

[176] Zhao H, Yan G, Zheng L, Zhou Y, Sheng H, Wu L, et al. STIM1 is a metabolic checkpoint regulating the invasion and metastasis of hepatocellular carcinoma. Theranostics. 2020;10(14):6483–99. 10.7150/thno.44025. 32483465 10.7150/thno.44025 PMC7255033

[177] Ndiaye H, Liu JY, Hall A, Minogue S, Morgan MY, Waugh MG. Immunohistochemical staining reveals differential expression of ACSL3 and ACSL4 in hepatocellular carcinoma and hepatic gastrointestinal metastases. 2020. Biosci Rep. 10.1042/bsr20200219. 10.1042/BSR20200219 PMC7198044 32286604

[178] Wang C, Chen Z, Yi Y, Ding Y, Xu F, Kang H, et al. RBM45 reprograms lipid metabolism promoting hepatocellular carcinoma via Rictor and ACSL1/ACSL4. Oncogene. 2024;43(5):328–40. 10.1038/s41388-023-02902-4. 38040804 10.1038/s41388-023-02902-4

[179] González-Romero F, Mestre D, Aurrekoetxea I, O’Rourke CJ, Andersen JB, Woodhoo A, et al. E2F1 and E2F2-mediated repression of CPT2 establishes a lipid-rich tumor-promoting environment. Cancer Res. 2021;81(11):2874–87. 10.1158/0008-5472.Can-20-2052. 33771899 10.1158/0008-5472.CAN-20-2052

[180] Fujiwara N, Nakagawa H, Enooku K, Kudo Y, Hayata Y, Nakatsuka T, et al. CPT2 downregulation adapts HCC to lipid-rich environment and promotes carcinogenesis via acylcarnitine accumulation in obesity. Gut. 2018;67(8):1493–504. 10.1136/gutjnl-2017-315193. 29437870 10.1136/gutjnl-2017-315193 PMC6039238

[181] Liu J, Sun B, Guo K, Yang Z, Zhao Y, Gao M, et al. Lipid-related FABP5 activation of tumor-associated monocytes fosters immune privilege via PD-L1 expression on Treg cells in hepatocellular carcinoma. Cancer Gene Ther. 2022;29(12):1951–60. 10.1038/s41417-022-00510-0. 35902729 10.1038/s41417-022-00510-0

[182] Wu L, Zhang X, Zheng L, Zhao H, Yan G, Zhang Q, et al. RIPK3 Orchestrates Fatty Acid Metabolism in Tumor-Associated Macrophages and Hepatocarcinogenesis. Cancer Immunol Res. 2020;8(5):710–21. 10.1158/2326-6066.Cir-19-0261. 32122992 10.1158/2326-6066.CIR-19-0261

[183] Che L, Chi W, Qiao Y, Zhang J, Song X, Liu Y, et al. Cholesterol biosynthesis supports the growth of hepatocarcinoma lesions depleted of fatty acid synthase in mice and humans. Gut. 2020;69(1):177–86. 10.1136/gutjnl-2018-317581. 30954949 10.1136/gutjnl-2018-317581 PMC6943247

[184] Zhang J, Liu B, Xu C, Ji C, Yin A, Liu Y, et al. Cholesterol homeostasis confers glioma malignancy triggered by hnRNPA2B1-dependent regulation of SREBP2 and LDLR. Neuro Oncol. 2024;26(4):684–700. 10.1093/neuonc/noad233. 38070488 10.1093/neuonc/noad233 PMC10995519

[185] Xiao MY, Pei WJ, Li S, Li FF, Xie P, Luo HT, et al. Gypenoside L inhibits hepatocellular carcinoma by targeting the SREBP2-HMGCS1 axis and enhancing immune response. Bioorg Chem. 2024;150:107539. 10.1016/j.bioorg.2024.107539. 38861912 10.1016/j.bioorg.2024.107539

[186] Wang Y, Wang J, Li X, Xiong X, Wang J, Zhou Z, et al. N(1)-methyladenosine methylation in tRNA drives liver tumourigenesis by regulating cholesterol metabolism. Nat Commun. 2021;12(1):6314. 10.1038/s41467-021-26718-6. 34728628 10.1038/s41467-021-26718-6 PMC8563902

[187] Jiang J, Zheng Q, Zhu W, Chen X, Lu H, Chen D, et al. Alterations in glycolytic/cholesterogenic gene expression in hepatocellular carcinoma. Aging (Albany NY). 2020;12(11):10300–16. 10.18632/aging.103254. 32479426 10.18632/aging.103254 PMC7346031

[188] Shao WQ, Zhu WW, Luo MJ, Fan MH, Li Q, Wang SH, et al. Cholesterol suppresses GOLM1-dependent selective autophagy of RTKs in hepatocellular carcinoma. Cell Rep. 2022;39(3):110712. 10.1016/j.celrep.2022.110712. 35443161 10.1016/j.celrep.2022.110712

[189] Mok EHK, Leung CON, Zhou L, Lei MML, Leung HW, Tong M, et al. Caspase-3-Induced Activation of SREBP2 Drives Drug Resistance via Promotion of Cholesterol Biosynthesis in Hepatocellular Carcinoma. Cancer Res. 2022;82(17):3102–15. 10.1158/0008-5472.Can-21-2934. 35767704 10.1158/0008-5472.CAN-21-2934

[190] Li Z, Wang Y, Xing R, Zeng H, Yu XJ, Zhang YJ, et al. Cholesterol efflux drives the generation of immunosuppressive macrophages to promote the progression of human hepatocellular carcinoma. Cancer Immunol Res. 2023;11(10):1400–13. 10.1158/2326-6066.Cir-22-0907. 37467346 10.1158/2326-6066.CIR-22-0907

[191] Cao J, Wang Z, Zhu M, Huang Y, Jin Z, Xiong Z. Low-density lipoprotein cholesterol and risk of hepatocellular carcinoma: a Mendelian randomization and mediation analysis. Lipids Health Dis. 2023;22(1):110. 10.1186/s12944-023-01877-1. 37525197 10.1186/s12944-023-01877-1 PMC10388495

[192] Zhu Y, Gu L, Lin X, Zhou X, Lu B, Liu C, et al. P53 deficiency affects cholesterol esterification to exacerbate hepatocarcinogenesis. Hepatology. 2023;77(5):1499–511. 10.1002/hep.32518. 35398929 10.1002/hep.32518 PMC11186660

[193] de la Conde Rosa L, Garcia-Ruiz C, Vallejo C, Baulies A, Nuñez S, Monte MJ, et al. STARD1 promotes NASH-driven HCC by sustaining the generation of bile acids through the alternative mitochondrial pathway. J Hepatol. 2021;74(6):1429–41. 10.1016/j.jhep.2021.01.028. 33515644 10.1016/j.jhep.2021.01.028 PMC8573791

[194] Huang J, Pan H, Sun J, Wu J, Xuan Q, Wang J, et al. TMEM147 aggravates the progression of HCC by modulating cholesterol homeostasis, suppressing ferroptosis, and promoting the M2 polarization of tumor-associated macrophages. J Exp Clin Cancer Res. 2023;42(1):286. 10.1186/s13046-023-02865-0. 37891677 10.1186/s13046-023-02865-0 PMC10612308

[195] Jin M, Yang Y, Dai Y, Cai R, Wu L, Jiao Y, et al. 27-Hydroxycholesterol is a specific factor in the neoplastic microenvironment of HCC that causes MDR via GRP75 regulation of the redox balance and metabolic reprogramming. Cell Biol Toxicol. 2022;38(2):311–24. 10.1007/s10565-021-09607-y. 33880675 10.1007/s10565-021-09607-y

[196] Yuan Y, Xu J, Jiang Q, Yang C, Wang N, Liu X, et al. Ficolin 3 promotes ferroptosis in HCC by downregulating IR/SREBP axis-mediated MUFA synthesis. J Exp Clin Cancer Res. 2024;43(1):133. 10.1186/s13046-024-03047-2. 38698462 10.1186/s13046-024-03047-2 PMC11067213

[197] Qiu Y, Wang X, Sun Y, Jin T, Tang R, Zhou X, et al. ACSL4-mediated membrane phospholipid remodeling induces integrin β1 activation to facilitate triple-negative breast cancer metastasis. Cancer Res. 2024;84(11):1856–71. 10.1158/0008-5472.Can-23-2491. 38471082 10.1158/0008-5472.CAN-23-2491 PMC11148537

[198] Holdaway CM, Leonard KA, Nelson R, van der Veen J, Das C, Watts R, et al. Alterations in phosphatidylethanolamine metabolism impacts hepatocellular lipid storage, energy homeostasis, and proliferation. Biochim Biophys Acta Mol Cell Biol Lipids. 2025;1870(4):159608. 10.1016/j.bbalip.2025.159608. 40154596 10.1016/j.bbalip.2025.159608

[199] Chen X, Lu T, Ding M, Cai Y, Yu Z, Zhou X, et al. Targeting YTHDF2 inhibits tumorigenesis of diffuse large B-cell lymphoma through ACER2-mediated ceramide catabolism. J Adv Res. 2024;63:17–33. 10.1016/j.jare.2023.10.010. 37865189 10.1016/j.jare.2023.10.010 PMC11379987

[200] Wang X, Guo W, Shi X, Chen Y, Yu Y, Du B, et al. S1PR1/S1PR3-YAP signaling and S1P-ALOX15 signaling contribute to an aggressive behavior in obesity-lymphoma. J Exp Clin Cancer Res. 2023;42(1):3. 10.1186/s13046-022-02589-7. 36600310 10.1186/s13046-022-02589-7 PMC9814427

[201] Jiang J, Gao Y, Wang J, Huang Y, Yang R, Zhang Y, et al. Hepatic sphingomyelin phosphodiesterase 3 promotes steatohepatitis by disrupting membrane sphingolipid metabolism. Cell Metab. 2025;37(5):1119-36.e13. 10.1016/j.cmet.2025.01.016.40015281 10.1016/j.cmet.2025.01.016

[202] Povero D, Chen Y, Johnson SM, McMahon CE, Pan M, Bao H, et al. HILPDA promotes NASH-driven HCC development by restraining intracellular fatty acid flux in hypoxia. J Hepatol. 2023;79(2):378–93. 10.1016/j.jhep.2023.03.041. 37061197 10.1016/j.jhep.2023.03.041 PMC11238876

[203] Yu M, Su M, Tian Z, Pan L, Li Z, Huang E, et al. Extracellular Vesicle-Packaged Linc-ZNF25-1 from Pancreatic Cancer Cell Promotes Pancreatic Stellate Cell Uptake of Asparagine to Advance Chemoresistance. Adv Sci (Weinh). 2025;12(16):e2413439. 10.1002/advs.202413439. 40041969 10.1002/advs.202413439 PMC12021039

[204] Yang J, Chen F, Lang L, Yang F, Fu Z, Martinez J, et al. Therapeutic targeting of the GLS1-c-Myc positive feedback loop suppresses glutaminolysis and inhibits progression of head and neck cancer. Cancer Res. 2024;84(19):3223–34. 10.1158/0008-5472.Can-24-0254. 39024547 10.1158/0008-5472.CAN-24-0254 PMC11444885

[205] Qu T, Song L, Xu J, Lu X, Yin D, Dai J, et al. MYLK-AS1 enhances glutamine metabolism to promote EGFR inhibitor resistance in non-small cell lung cancer. Cancer Res. 2025;85(16):3052–71. 10.1158/0008-5472.Can-23-3748. 40366631 10.1158/0008-5472.CAN-23-3748

[206] Adiamah M, Poole B, Lindsey JC, Kohe S, Morcavallo A, Burté F, et al. MYC-dependent upregulation of the de novo serine and glycine synthesis pathway is a targetable metabolic vulnerability in group 3 medulloblastoma. Neuro Oncol. 2025;27(1):237–53. 10.1093/neuonc/noae179. 39377369 10.1093/neuonc/noae179 PMC11726242

[207] Situ Y, Zhang J, Liao W, Liang Q, Lu L, Xu Q, et al. SHMT as a potential therapeutic target for renal cell carcinoma. Front Biosci (Landmark Ed). 2023;28(9):196. 10.31083/j.fbl2809196. 37796681 10.31083/j.fbl2809196

[208] Fang K, Sun M, Leng Z, Chu Y, Zhao Z, Li Z, et al. Targeting IGF1R signaling enhances the sensitivity of cisplatin by inhibiting proline and arginine metabolism in oesophageal squamous cell carcinoma under hypoxia. J Exp Clin Cancer Res. 2023;42(1):73. 10.1186/s13046-023-02623-2. 36978187 10.1186/s13046-023-02623-2 PMC10044411

[209] Wang M, Li J, Yang X, Yan Q, Wang H, Xu X, et al. Targeting TLK2 inhibits the progression of gastric cancer by reprogramming amino acid metabolism through the mTOR/ASNS axis. Cancer Gene Ther. 2023;30(11):1485–97. 10.1038/s41417-023-00653-8. 37542132 10.1038/s41417-023-00653-8

[210] Li J, Fang Z, Dal E, Zhang H, Yu K, Ma M, et al. Transmembrane protein 176B regulates amino acid metabolism through the PI3K-Akt-mTOR signaling pathway and promotes gastric cancer progression. Cancer Cell Int. 2024;24(1):95. 10.1186/s12935-024-03279-4. 38438907 10.1186/s12935-024-03279-4 PMC10913232

[211] Amleh A, Chen HP, Watad L, Abramovich I, Agranovich B, Gottlieb E, et al. Arginine depletion attenuates renal cystogenesis in tuberous sclerosis complex model. Cell Rep Med. 2023;4(6):101073. 10.1016/j.xcrm.2023.101073. 37290438 10.1016/j.xcrm.2023.101073 PMC10313931

[212] Bian Y, Yin G, Wang G, Liu T, Liang L, Yang X, et al. Degradation of HIF-1α induced by curcumol blocks glutaminolysis and inhibits epithelial-mesenchymal transition and invasion in colorectal cancer cells. Cell Biol Toxicol. 2023;39(5):1957–78. 10.1007/s10565-021-09681-2. 35083610 10.1007/s10565-021-09681-2

[213] Yun HJ, Li M, Guo D, Jeon SM, Park SH, Lim JS, et al. AMPK-HIF-1α signaling enhances glucose-derived de novo serine biosynthesis to promote glioblastoma growth. J Exp Clin Cancer Res. 2023;42(1):340. 10.1186/s13046-023-02927-3. 38098117 10.1186/s13046-023-02927-3 PMC10722853

[214] Wang SQ, Liu N, Zhang Q, Li BQ, Zhao FY, Li C, et al. TFE3 and HIF1α regulates the expression of SHMT2 isoforms via alternative promoter utilization in ovarian cancer cells. Cell Death Dis. 2025;16(1):178. 10.1038/s41419-025-07445-y. 40097394 10.1038/s41419-025-07445-y PMC11914208

[215] van der Mijn JC, Chen Q, Laursen KB, Khani F, Wang X, Dorsaint P, et al. Transcriptional and metabolic remodeling in clear cell renal cell carcinoma caused by ATF4 activation and the integrated stress response (ISR). Mol Carcinog. 2022;61(9):851–64. 10.1002/mc.23437. 35726553 10.1002/mc.23437 PMC9378514

[216] Zhang ZJ, Wu QF, Ren AQ, Chen Q, Shi JZ, Li JP, et al. ATF4 renders human T-cell acute lymphoblastic leukemia cell resistance to FGFR1 inhibitors through amino acid metabolic reprogramming. Acta Pharmacol Sin. 2023;44(11):2282–95. 10.1038/s41401-023-01108-4. 37280363 10.1038/s41401-023-01108-4 PMC10618259

[217] Tian Z, Su M, Yu M, Huang E, Hu B, Chen Y. KRAS/ACTN4/p65-NR2A axis mediates glutamine-glutamate metabolic coupling between schwann cells and pancreatic cancer promoting perineural invasion. J Adv Res. 2025. 10.1016/j.jare.2025.10.030.41135873 10.1016/j.jare.2025.10.030

[218] Guo S, Xing S, Wu Z, Chen F, Pan X, Li Q, et al. Leucine restriction ameliorates *Fusobacterium nucleatum*-driven malignant progression and radioresistance in nasopharyngeal carcinoma. Cell Rep Med. 2024;5(10):101753. 10.1016/j.xcrm.2024.101753. 39357525 10.1016/j.xcrm.2024.101753 PMC11513822

[219] He J, Lin M, Zhang X, Zhang R, Tian T, Zhou Y, et al. TET2 is required to suppress mTORC1 signaling through urea cycle with therapeutic potential. Cell Discov. 2023;9(1):84. 10.1038/s41421-023-00567-7. 37550284 10.1038/s41421-023-00567-7 PMC10406918

[220] Figlia G, Müller S, Garcia-Cortizo F, Neff M, Klinke G, Poschet G, et al. mTORC1 senses glutamine and other amino acids through GCN2. EMBO J. 2025;44(17):4825–66. 10.1038/s44318-025-00505-1. 40691417 10.1038/s44318-025-00505-1 PMC12402317

[221] Feng HG, Wu CX, Zhong GC, Gong JP, Miao CM, Xiong B. Integrative analysis reveals that SLC38A1 promotes hepatocellular carcinoma development via PI3K/AKT/mTOR signaling via glutamine mediated energy metabolism. J Cancer Res Clin Oncol. 2023;149(17):15879–98. 10.1007/s00432-023-05360-3. 37673823 10.1007/s00432-023-05360-3 PMC11796876

[222] Liu X. SLC family transporters. Adv Exp Med Biol. 2019;1141:101–202. 10.1007/978-981-13-7647-4_3. 31571165 10.1007/978-981-13-7647-4_3

[223] Tambay V, Raymond VA, Voisin L, Meloche S, Bilodeau M. Reprogramming of glutamine amino acid transporters expression and prognostic significance in hepatocellular carcinoma. Int J Mol Sci. 2024. 10.3390/ijms25147558. 39062801 10.3390/ijms25147558 PMC11277143

[224] Chai B, Zhang A, Liu Y, Zhang X, Kong P, Zhang Z, et al. KLF7 promotes hepatocellular carcinoma progression through regulating SLC1A5-mediated tryptophan metabolism. J Cell Mol Med. 2024;28(23):e70245. 10.1111/jcmm.70245. 39648156 10.1111/jcmm.70245 PMC11625504

[225] Shi X, Zhang Y, Wang Y, Wang J, Gao Y, Wang R, et al. The tRNA Gm18 methyltransferase TARBP1 promotes hepatocellular carcinoma progression via metabolic reprogramming of glutamine. Cell Death Differ. 2024. 10.1038/s41418-024-01323-4. 38867004 10.1038/s41418-024-01323-4 PMC11368932

[226] Zhang T, Cui Y, Wu Y, Meng J, Han L, Zhang J, et al. Mitochondrial GCN5L1 regulates glutaminase acetylation and hepatocellular carcinoma. Clin Transl Med. 2022;12(5):e852. 10.1002/ctm2.852. 35538890 10.1002/ctm2.852 PMC9091986

[227] Li B, Cao Y, Meng G, Qian L, Xu T, Yan C, et al. Targeting glutaminase 1 attenuates stemness properties in hepatocellular carcinoma by increasing reactive oxygen species and suppressing Wnt/beta-catenin pathway. EBioMedicine. 2019;39:239–54. 10.1016/j.ebiom.2018.11.063. 30555042 10.1016/j.ebiom.2018.11.063 PMC6355660

[228] Huang X, Gan G, Wang X, Xu T, Xie W. The HGF-MET axis coordinates liver cancer metabolism and autophagy for chemotherapeutic resistance. Autophagy. 2019;15(7):1258–79. 10.1080/15548627.2019.1580105. 30786811 10.1080/15548627.2019.1580105 PMC6613896

[229] Tian R, Li Y, Shen X, Li Y. Targeting PTBP1 blocks glutamine metabolism to improve the cisplatin sensitivity of hepatocarcinoma cells through modulating the mRNA stability of glutaminase. Open Med (Wars). 2023;18(1):20230756. 10.1515/med-2023-0756. 37724122 10.1515/med-2023-0756 PMC10505300

[230] Lu H, Yin H, Qu L, Ma X, Fu R, Fan D. Ginsenoside Rk1 regulates glutamine metabolism in hepatocellular carcinoma through inhibition of the ERK/c-Myc pathway. Food Funct. 2022;13(7):3793–811. 10.1039/d1fo03728e. 35316310 10.1039/d1fo03728e

[231] Xu K, Ding J, Zhou L, Li D, Luo J, Wang W, et al. SMYD2 promotes hepatocellular carcinoma progression by reprogramming glutamine metabolism via c-Myc/GLS1 axis. Cells. 2022. 10.3390/cells12010025. 36611819 10.3390/cells12010025 PMC9818721

[232] Zeng Y, Jiang H, Zhang X, Xu J, Wu X, Xu Q, et al. Canagliflozin reduces chemoresistance in hepatocellular carcinoma through PKM2-c-Myc complex-mediated glutamine starvation. Free Radic Biol Med. 2023;208:571–86. 10.1016/j.freeradbiomed.2023.09.006. 37696420 10.1016/j.freeradbiomed.2023.09.006

[233] Deng J, Li Y, Yin L, Liu S, Li Y, Liao W, et al. Histone lactylation enhances GCLC expression and thus promotes chemoresistance of colorectal cancer stem cells through inhibiting ferroptosis. Cell Death Dis. 2025;16(1):193. 10.1038/s41419-025-07498-z. 40113760 10.1038/s41419-025-07498-z PMC11926133

[234] Liu X, Cao Z, Wang W, Zou C, Wang Y, Pan L, et al. Engineered extracellular vesicle-delivered CRISPR/Cas9 for radiotherapy sensitization of glioblastoma. ACS Nano. 2023;17(17):16432–47. 10.1021/acsnano.2c12857. 37646615 10.1021/acsnano.2c12857 PMC10510715

[235] Suzuki S, Venkatesh D, Kanda H, Nakayama A, Hosokawa H, Lee E, et al. GLS2 is a tumor suppressor and a regulator of ferroptosis in hepatocellular carcinoma. Cancer Res. 2022;82(18):3209–22. 10.1158/0008-5472.Can-21-3914. 35895807 10.1158/0008-5472.CAN-21-3914 PMC11057045

[236] Thomas D, Wu M, Nakauchi Y, Zheng M, Thompson-Peach CAL, Lim K, et al. Dysregulated Lipid Synthesis by Oncogenic IDH1 Mutation Is a Targetable Synthetic Lethal Vulnerability. Cancer Discov. 2023;13(2):496–515. 10.1158/2159-8290.Cd-21-0218. 36355448 10.1158/2159-8290.CD-21-0218 PMC9900324

[237] Gunn K, Myllykoski M, Cao JZ, Ahmed M, Huang B, Rouaisnel B, et al. (R)-2-hydroxyglutarate inhibits KDM5 histone lysine demethylases to drive transformation in IDH-mutant cancers. Cancer Discov. 2023;13(6):1478–97. 10.1158/2159-8290.Cd-22-0825. 36847506 10.1158/2159-8290.CD-22-0825 PMC10238656

[238] Yang C, Ko B, Hensley CT, Jiang L, Wasti AT, Kim J, et al. Glutamine oxidation maintains the TCA cycle and cell survival during impaired mitochondrial pyruvate transport. Mol Cell. 2014;56(3):414–24. 10.1016/j.molcel.2014.09.025. 25458842 10.1016/j.molcel.2014.09.025 PMC4268166

[239] Ghosh A, Ghosh AK, Zaman A, Das PK. Metformin-loaded hyaluronic acid-derived carbon dots for targeted therapy against hepatocellular carcinoma by glutamine metabolic reprogramming. Mol Pharm. 2023;20(12):6391–406. 10.1021/acs.molpharmaceut.3c00772. 37933877 10.1021/acs.molpharmaceut.3c00772

[240] Wang B, Pei J, Xu S, Liu J, Yu J. System analysis based on glutamine catabolic-related enzymes identifies GPT2 as a novel immunotherapy target for lung adenocarcinoma. Comput Biol Med. 2023;165:107415. 10.1016/j.compbiomed.2023.107415. 37657356 10.1016/j.compbiomed.2023.107415

[241] Li MR, Li JZ, Li JY, Wang CC, Yuan RK, Ye LH, et al. Clinical features of non-alcoholic fatty liver disease in the non-lean population. Obes Facts. 2023;16(5):427–34. 10.1159/000530845. 37231905 10.1159/000530845 PMC10601616

[242] Wang Y, Cheng C, Lu Y, Lian Z, Liu Q, Xu Y, et al. β-Catenin activation reprograms ammonia metabolism to promote senescence resistance in hepatocellular carcinoma. Cancer Res. 2024;84(10):1643–58. 10.1158/0008-5472.Can-23-0673. 38417136 10.1158/0008-5472.CAN-23-0673

[243] You HJ, Li Q, Ma LH, Wang X, Zhang HY, Wang YX, et al. Inhibition of GLUD1 mediated by LASP1 and SYVN1 contributes to hepatitis B virus X protein-induced hepatocarcinogenesis. J Mol Cell Biol. 2024. 10.1093/jmcb/mjae014. 38587834 10.1093/jmcb/mjae014 PMC11440430

[244] Zhou Y, Yu H, Cheng S, Chen Y, He L, Ren J, et al. Glutamate dehydrogenase 1 mediated glutaminolysis sustains HCC cells survival under glucose deprivation. J Cancer. 2022;13(3):1061–72. 10.7150/jca.64195. 35154470 10.7150/jca.64195 PMC8824882

[245] Wei Y, Tang X, Ren Y, Yang Y, Song F, Fu J, et al. An RNA-RNA crosstalk network involving HMGB1 and RICTOR facilitates hepatocellular carcinoma tumorigenesis by promoting glutamine metabolism and impedes immunotherapy by PD-L1+ exosomes activity. Signal Transduct Target Ther. 2021;6(1):421. 10.1038/s41392-021-00801-2. 34916485 10.1038/s41392-021-00801-2 PMC8677721

[246] Liu P, Lu D, Al-Ameri A, Wei X, Ling S, Li J, et al. Glutamine synthetase promotes tumor invasion in hepatocellular carcinoma through mediating epithelial-mesenchymal transition. Hepatol Res. 2020;50(2):246–57. 10.1111/hepr.13433. 31652385 10.1111/hepr.13433

[247] Kurebayashi Y, Tsujikawa H, Sugimoto K, Yunaiyama D, Araki Y, Saito K, et al. Tumor steatosis and glutamine synthetase expression in patients with advanced hepatocellular carcinoma receiving atezolizumab plus bevacizumab therapy. Hepatol Res. 2023;53(10):1008–20. 10.1111/hepr.13933. 37300323 10.1111/hepr.13933

[248] Shao M, Tao Q, Xu Y, Xu Q, Shu Y, Chen Y, et al. Glutamine synthetase-negative hepatocellular carcinoma has better prognosis and response to sorafenib treatment after hepatectomy. Chin Med J (Engl). 2023;136(17):2066–76. 10.1097/cm9.0000000000002380. 37249521 10.1097/CM9.0000000000002380 PMC10476731

[249] Jiang J, Hu Y, Fang D, Luo J. Glutamine synthetase and hepatocellular carcinoma. Clin Res Hepatol Gastroenterol. 2023;47(10):102248. 10.1016/j.clinre.2023.102248. 37979911 10.1016/j.clinre.2023.102248

[250] He M, Huang Y, Du Z, Lai Z, Ouyang H, Shen J, et al. Lenvatinib, Toripalimab plus FOLFOX Chemotherapy in Hepatocellular Carcinoma Patients with Extrahepatic Metastasis: A Biomolecular Exploratory, Phase II Trial (LTSC). Clin Cancer Res. 2023;29(24):5104–15. 10.1158/1078-0432.Ccr-23-0060. 37819944 10.1158/1078-0432.CCR-23-0060

[251] Millman SE, Chaves-Perez A, Janaki-Raman S, Ho YJ, Morris JPt, Narendra V, et al. α-ketoglutarate dehydrogenase is a therapeutic vulnerability in acute myeloid leukemia. Blood. 2025;145(13):1422–36. 10.1182/blood.2024025245. 39791576 10.1182/blood.2024025245 PMC11969269

[252] Lim LQJ, Adler L, Hajaj E, Soria LR, Perry RB, Darzi N, et al. ASS1 metabolically contributes to the nuclear and cytosolic p53-mediated DNA damage response. Nat Metab. 2024;6(7):1294–309. 10.1038/s42255-024-01060-5. 38858597 10.1038/s42255-024-01060-5 PMC11272581

[253] Bai J, Tang R, Zhou K, Chang J, Wang H, Zhang Q, et al. An asparagine metabolism-based classification reveals the metabolic and immune heterogeneity of hepatocellular carcinoma. BMC Med Genomics. 2022;15(1):222. 10.1186/s12920-022-01380-z. 36284275 10.1186/s12920-022-01380-z PMC9594908

[254] Liu Y, Miao Z, Yang Q. AGC1-mediated Metabolic Reprogramming and Autophagy Sustain Survival of Hepatocellular Carcinoma Cells under Glutamine Deprivation. Cell Biochem Biophys. 2024. 10.1007/s12013-024-01311-y. 38789662 10.1007/s12013-024-01311-y

[255] Özdemir F, Didem Orhan M, Atasavum ZT, Tülek A. Biochemical characterization and detection of antitumor activity of l-asparaginase from thermophilic *Geobacillus kaustophilus* DSM 7263(T). Protein Expr Purif. 2022;199:106146. 10.1016/j.pep.2022.106146. 35863721 10.1016/j.pep.2022.106146

[256] Okuda K, Umemura A, Kataoka S, Yano K, Takahashi A, Okishio S, et al. Enhanced Antitumor Effect in Liver Cancer by Amino Acid Depletion-Induced Oxidative Stress. Front Oncol. 2021;11:758549. 10.3389/fonc.2021.758549. 34796113 10.3389/fonc.2021.758549 PMC8593418

[257] Riaz A, Kaleem A, Abdullah R, Iqtedar M, Hoessli DC, Aftab M. In silico approaches to study the human asparagine synthetase: an insight of the interaction between the enzyme active sites and its substrates. PLoS ONE. 2024;19(8):e0307448. 10.1371/journal.pone.0307448. 39093903 10.1371/journal.pone.0307448 PMC11296641

[258] Zhang B, Dong LW, Tan YX, Zhang J, Pan YF, Yang C, et al. Asparagine synthetase is an independent predictor of surgical survival and a potential therapeutic target in hepatocellular carcinoma. Br J Cancer. 2013;109(1):14–23. 10.1038/bjc.2013.293. 23764751 10.1038/bjc.2013.293 PMC3708586

[259] Zhou Q, Li L, Sha F, Lei Y, Tian X, Chen L, et al. PTTG1 reprograms asparagine metabolism to promote hepatocellular carcinoma progression. Cancer Res. 2023;83(14):2372–86. 10.1158/0008-5472.Can-22-3561. 37159932 10.1158/0008-5472.CAN-22-3561

[260] Li W, Dong C. Polymorphism in asparagine synthetase is associated with overall survival of hepatocellular carcinoma patients. BMC Gastroenterol. 2017;17(1):79. 10.1186/s12876-017-0635-4. 28629319 10.1186/s12876-017-0635-4 PMC5477286

[261] Wang K, Luo L, Fu S, Wang M, Wang Z, Dong L, et al. PHGDH arginine methylation by PRMT1 promotes serine synthesis and represents a therapeutic vulnerability in hepatocellular carcinoma. Nat Commun. 2023;14(1):1011. 10.1038/s41467-023-36708-5. 36823188 10.1038/s41467-023-36708-5 PMC9950448

[262] Ma S, Sandhoff R, Luo X, Shang F, Shi Q, Li Z, et al. Serine enrichment in tumors promotes regulatory T cell accumulation through sphinganine-mediated regulation of c-Fos. Sci Immunol. 2024;9(94):eadg8817. 10.1126/sciimmunol.adg8817. 38640251 10.1126/sciimmunol.adg8817

[263] Lu Y, Zhu J, Zhang Y, Li W, Xiong Y, Fan Y, et al. Lactylation-driven IGF2BP3-mediated serine metabolism reprogramming and RNA m6A-modification promotes lenvatinib resistance in HCC. Adv Sci Weinh. 2024;11(46):e2401399. 10.1002/advs.202401399. 39450426 10.1002/advs.202401399 PMC11633555

[264] Liu X, Liu B, Wang J, Liu H, Wu J, Qi Y, et al. PHGDH activation fuels glioblastoma progression and radioresistance via serine synthesis pathway. J Exp Clin Cancer Res. 2025;44(1):99. 10.1186/s13046-025-03361-3. 40102981 10.1186/s13046-025-03361-3 PMC11921657

[265] Huang Z, Zhang K, Jiang Y, Wang M, Li M, Guo Y, et al. Molecular glue triggers degradation of PHGDH by enhancing the interaction between DDB1 and PHGDH. Acta Pharm Sin B. 2024;14(9):4001–13. 10.1016/j.apsb.2024.06.001. 39309493 10.1016/j.apsb.2024.06.001 PMC11413658

[266] O’Connor C, Schneider M, Katinas JM, Nayeen MJ, Shah K, Magdum T, et al. Role of mitochondrial and cytosolic folylpolyglutamate synthetase in one-carbon metabolism and antitumor efficacy of mitochondrial-targeted antifolates. Mol Pharmacol. 2024;106(4):173–87. 10.1124/molpharm.124.000912. 39048308 10.1124/molpharm.124.000912 PMC11413923

[267] Green AC, Marttila P, Kiweler N, Chalkiadaki C, Wiita E, Cookson V, et al. Formate overflow drives toxic folate trapping in MTHFD1 inhibited cancer cells. Nat Metab. 2023;5(4):642–59. 10.1038/s42255-023-00771-5. 37012496 10.1038/s42255-023-00771-5 PMC10132981

[268] Wang J, Zeng L, Wu N, Liang Y, Jin J, Fan M, et al. Inhibition of phosphoglycerate dehydrogenase induces ferroptosis and overcomes enzalutamide resistance in castration-resistant prostate cancer cells. Drug Resist Updat. 2023;70:100985. 10.1016/j.drup.2023.100985. 37423117 10.1016/j.drup.2023.100985

[269] Deng H, Wang Y, Xiao L, Feng M, Dou W, Pan Y. SHMT inhibitor synergizes with 5-Fu to suppress gastric cancer via cell cycle arrest and chemoresistance alleviation. NPJ Precis Oncol. 2025;9(1):135. 10.1038/s41698-025-00926-5. 40346149 10.1038/s41698-025-00926-5 PMC12064653

[270] Hu Q, Dai J, Zhang Z, Yu H, Zhang J, Zhu X, et al. ASS1-mediated reductive carboxylation of cytosolic glutamine confers ferroptosis resistance in cancer cells. Cancer Res. 2023;83(10):1646–65. 10.1158/0008-5472.Can-22-1999. 36892426 10.1158/0008-5472.CAN-22-1999

[271] Yang C, Pataskar A, Feng X, Montenegro Navarro J, Paniagua I, Jacobs JJL, et al. Arginine deprivation enriches lung cancer proteomes with cysteine by inducing arginine-to-cysteine substitutants. Mol Cell. 2024;84(10):1904-16.e7. 10.1016/j.molcel.2024.04.012. 38759626 10.1016/j.molcel.2024.04.012 PMC11129317

[272] Bibi K, Fatima T, Sohrab S, Haider G, Zarina S, Ilyas A. Polymorphic variants of ASS1 gene related to arginine metabolism and the risk of HCC. Protein Pept Lett. 2023;30(7):587–96. 10.2174/0929866530666230529143121. 37254538 10.2174/0929866530666230529143121

[273] Luo W, Zou Z, Nie Y, Luo J, Ming Z, Hu X, et al. ASS1 inhibits triple-negative breast cancer by regulating PHGDH stability and de novo serine synthesis. Cell Death Dis. 2024;15(5):319. 10.1038/s41419-024-06672-z. 38710705 10.1038/s41419-024-06672-z PMC11074131

[274] Mossmann D, Müller C, Park S, Ryback B, Colombi M, Ritter N, et al. Arginine reprograms metabolism in liver cancer via RBM39. Cell. 2023;186(23):5068-83.e23. 10.1016/j.cell.2023.09.011. 37804830 10.1016/j.cell.2023.09.011 PMC10642370

[275] Zhao M, Yuan D, Wei M, Zhang J, Yang W, Qin S, et al. FOXO1-mediated argininosuccinate lyase transcription inhibits ammonia metabolism and breast cancer cell metastasis. J Biol Chem. 2025;301(10):110677. 10.1016/j.jbc.2025.110677. 40907897 10.1016/j.jbc.2025.110677 PMC12509978

[276] Geng X, Li M, Zhang L, Cai Y, Chen X, Mu X, et al. P5CS deacetylation mediated by SIRT2 facilitates tumor growth by enhancing mitochondrial respiration in hepatocellular carcinoma. Oncogene. 2025;44(31):2746–61. 10.1038/s41388-025-03456-3. 40425834 10.1038/s41388-025-03456-3

[277] Ding Z, Ericksen RE, Escande-Beillard N, Lee QY, Loh A, Denil S, et al. Metabolic pathway analyses identify proline biosynthesis pathway as a promoter of liver tumorigenesis. J Hepatol. 2020;72(4):725–35. 10.1016/j.jhep.2019.10.026. 31726117 10.1016/j.jhep.2019.10.026

[278] Qiao W, Wang H, Zhang X, Luo K. Proline-rich protein 11 silencing inhibits hepatocellular carcinoma growth and epithelial-mesenchymal transition through β-catenin signaling. Gene. 2019;681:7–14. 10.1016/j.gene.2018.09.036. 30248355 10.1016/j.gene.2018.09.036

[279] Tang L, Zeng J, Geng P, Fang C, Wang Y, Sun M, et al. Global metabolic profiling identifies a pivotal role of proline and hydroxyproline metabolism in supporting hypoxic response in hepatocellular carcinoma. Clin Cancer Res. 2018;24(2):474–85. 10.1158/1078-0432.Ccr-17-1707. 29084919 10.1158/1078-0432.CCR-17-1707

[280] Phang JM. The regulatory mechanisms of proline and hydroxyproline metabolism: recent advances in perspective. Front Oncol. 2022;12:1118675. 10.3389/fonc.2022.1118675. 36818667 10.3389/fonc.2022.1118675 PMC9930595

[281] Li Z, Liu Z, Lin M, Pan H, Liu Y, Liu Y, et al. Acetylation-induced degradation of ECHS1 enhances BCAA accumulation and proliferation in KRAS-mutant colorectal cancer. J Exp Clin Cancer Res. 2025;44(1):164. 10.1186/s13046-025-03399-3. 40437561 10.1186/s13046-025-03399-3 PMC12117712

[282] Wang N, Lu S, Cao Z, Li H, Xu J, Zhou Q, et al. Pyruvate metabolism enzyme DLAT promotes tumorigenesis by suppressing leucine catabolism. Cell Metab. 2025;37(6):1381-99.e9. 10.1016/j.cmet.2025.02.008. 40112809 10.1016/j.cmet.2025.02.008

[283] Chen J, Liu X, Zou Y, Gong J, Ge Z, Lin X, et al. A high-fat diet promotes cancer progression by inducing gut microbiota-mediated leucine production and PMN-MDSC differentiation. Proc Natl Acad Sci U S A. 2024;121(20):e2306776121. 10.1073/pnas.2306776121. 38709933 10.1073/pnas.2306776121 PMC11098111

[284] Hiraoka A, Kato M, Marui K, Murakami T, Onishi K, Adachi T, et al. Easy clinical predictor for low BCAA to tyrosine ratio in chronic liver disease patients with hepatocellular carcinoma: Usefulness of ALBI score as nutritional prognostic marker. Cancer Med. 2021;10(11):3584–92. 10.1002/cam4.3908. 33960691 10.1002/cam4.3908 PMC8178498

[285] Ghanem SE, Abdel-Samiee M, El-Said H, Youssef MI, ElZohry HA, Abdelsameea E, et al. Evaluation of amino acids profile as non-invasive biomarkers of hepatocellular carcinoma in Egyptians. Trop Med Infect Dis. 2022. 10.3390/tropicalmed7120437. 36548692 10.3390/tropicalmed7120437 PMC9786038

[286] Qian L, Li N, Lu XC, Xu M, Liu Y, Li K, et al. Enhanced BCAT1 activity and BCAA metabolism promotes RhoC activity in cancer progression. Nat Metab. 2023;5(7):1159–73. 10.1038/s42255-023-00818-7. 37337119 10.1038/s42255-023-00818-7

[287] Ibrahim SL, Abed MN, Mohamed G, Price JC, Abdullah MI, Richardson A. Inhibition of branched-chain alpha-keto acid dehydrogenase kinase augments the sensitivity of ovarian and breast cancer cells to paclitaxel. Br J Cancer. 2023;128(5):896–906. 10.1038/s41416-022-02095-9. 36526674 10.1038/s41416-022-02095-9 PMC9977917

[288] Bo T, Osaki T, Fujii J. Dephosphorylation of branched-chain α-keto acid dehydrogenase E1α (BCKDHA) promotes branched-chain amino acid catabolism and renders cancer cells resistant to X-rays by mitigating DNA damage. Biochem Biophys Res Commun. 2025;742:151154. 10.1016/j.bbrc.2024.151154. 39672007 10.1016/j.bbrc.2024.151154

[289] Lin K, Wei L, Wang R, Li L, Song S, Wang F, et al. Disrupted methionine cycle triggers muscle atrophy in cancer cachexia through epigenetic regulation of REDD1. Cell Metab. 2025;37(2):460-76.e8. 10.1016/j.cmet.2024.10.017. 39729999 10.1016/j.cmet.2024.10.017

[290] Fan Y, Wang Y, Dan W, Zhang Y, Nie L, Ma Z, et al. PRMT5-mediated arginine methylation stabilizes GPX4 to suppress ferroptosis in cancer. Nat Cell Biol. 2025;27(4):641–53. 10.1038/s41556-025-01610-3. 40033101 10.1038/s41556-025-01610-3

[291] Han T, Wang Y, Cheng M, Hu Q, Wan X, Huang M, et al. Phosphorylated SHMT2 regulates oncogenesis through m(6)A modification in lung adenocarcinoma. Adv Sci (Weinh). 2024;11(18):e2307834. 10.1002/advs.202307834. 38460155 10.1002/advs.202307834 PMC11095143

[292] Gou D, Liu R, Shan X, Deng H, Chen C, Xiang J, et al. Gluconeogenic enzyme PCK1 supports S-adenosylmethionine biosynthesis and promotes H3K9me3 modification to suppress hepatocellular carcinoma progression. J Clin Invest. 2023. 10.1172/jci161713. 37166978 10.1172/JCI161713 PMC10313362

[293] Shen S, Liu R, Huang J, Sun Y, Tan Q, Luo Q. MAT1A activation of glycolysis to promote NSCLC progression depends on stabilizing CCND1. Cell Death Dis. 2024;15(10):768. 10.1038/s41419-024-07113-7. 39438468 10.1038/s41419-024-07113-7 PMC11496809

[294] Li F, Liu P, Mi W, Li L, Anderson NM, Lesner NP, et al. Blocking methionine catabolism induces senescence and confers vulnerability to GSK3 inhibition in liver cancer. Nat Cancer. 2024;5(1):131–46. 10.1038/s43018-023-00671-3. 38168934 10.1038/s43018-023-00671-3 PMC11277537

[295] Guo J, Buettner R, Du L, Li Z, Liu W, Su R, et al. 8-Cl-Ado and 8-NH(2)-Ado synergize with venetoclax to target the methionine-MAT2A-SAM axis in acute myeloid leukemia. Leukemia. 2024;38(6):1236–45. 10.1038/s41375-024-02222-w. 38643304 10.1038/s41375-024-02222-w PMC11147765

[296] Chen R, Ma C, Qian H, Xie X, Zhang Y, Lu D, et al. Mutant KRAS and CK2 cooperatively stimulate SLC16A3 activity to drive intrahepatic cholangiocarcinoma progression. Cancer Res. 2025;85(7):1253–69. 10.1158/0008-5472.Can-24-2097. 39854318 10.1158/0008-5472.CAN-24-2097

[297] Fu Z, Deng M, Zhou Q, Li S, Liu W, Cao S, et al. Arsenic activated GLUT1-mTORC1/HIF-1α-PKM2 positive feedback networks promote proliferation and migration of bladder epithelial cells. Sci Total Environ. 2024;947:174538. 10.1016/j.scitotenv.2024.174538. 38977090 10.1016/j.scitotenv.2024.174538

[298] Chen ST, Chang KS, Lin YH, Hou CP, Lin WY, Hsu SY, et al. Glucose Upregulates ChREBP via Phosphorylation of AKT and AMPK to Modulate MALT1 and WISP1 Expression. J Cell Physiol. 2025;240(1):e31478. 10.1002/jcp.31478. 39530300 10.1002/jcp.31478

[299] Engeler M, Karim M, Gischke M, Willer F, Leiner H, Prey J, et al. Carbohydrate-responsive element-binding protein-associated metabolic changes in chemically induced hepatocarcinogenesis mouse model. Int J Mol Sci. 2025. 10.3390/ijms26146932. 40725179 10.3390/ijms26146932 PMC12295894

[300] Zhang F, Wu Z, Xiang Y, He Q, Li W, Yang K, et al. SOX4 reprograms fatty acid metabolism through the CHREBP to inhibit ferroptosis in hepatocellular carcinoma. Cell Death Discov. 2025;11(1):246. 10.1038/s41420-025-02527-4. 40399256 10.1038/s41420-025-02527-4 PMC12095664

[301] Karim M, Prey J, Willer F, Leiner H, Yasser M, Dombrowski F, et al. Hepatic deletion of carbohydrate response element binding protein impairs hepatocarcinogenesis in a high-fat diet-induced mouse model. Int J Mol Sci. 2025. 10.3390/ijms26052246. 40076869 10.3390/ijms26052246 PMC11900174

[302] Meng Y, Guo D, Lin L, Zhao H, Xu W, Luo S, et al. Glycolytic enzyme PFKL governs lipolysis by promoting lipid droplet-mitochondria tethering to enhance β-oxidation and tumor cell proliferation. Nat Metab. 2024;6(6):1092–107. 10.1038/s42255-024-01047-2. 38773347 10.1038/s42255-024-01047-2

[303] Chen YJ, Liao WX, Huang SZ, Yu YF, Wen JY, Chen J, et al. Prognostic and immunological role of CD36: a pan-cancer analysis. J Cancer. 2021;12(16):4762–73. 10.7150/jca.50502. 34234847 10.7150/jca.50502 PMC8247371

[304] Cassim S, Raymond VA, Dehbidi-Assadzadeh L, Lapierre P, Bilodeau M. Metabolic reprogramming enables hepatocarcinoma cells to efficiently adapt and survive to a nutrient-restricted microenvironment. Cell Cycle. 2018;17(7):903–16. 10.1080/15384101.2018.1460023. 29633904 10.1080/15384101.2018.1460023 PMC6056217

[305] Zeb A, Khan W, Ul Islam W, Khan F, Khan A, Khan H, et al. Exploring the Anticancer Potential of Astragalin in Triple Negative Breast Cancer Cells by Attenuating Glycolytic Pathway through AMPK/mTOR. Curr Med Chem. 2024. 10.2174/0109298673304759240722064518. 39069711 10.2174/0109298673304759240722064518

[306] Wang MD, Wu H, Fu GB, Zhang HL, Zhou X, Tang L, et al. Acetyl-coenzyme A carboxylase alpha promotion of glucose-mediated fatty acid synthesis enhances survival of hepatocellular carcinoma in mice and patients. Hepatology. 2016;63(4):1272–86. 10.1002/hep.28415. 26698170 10.1002/hep.28415

[307] Shen J, Wu Z, Zhou Y, Yang D, Wang X, Yu B, et al. Knockdown of SLC16A3 decreases extracellular lactate concentration in hepatocellular carcinoma, alleviates hypoxia and induces ferroptosis. Biochem Biophys Res Commun. 2024;733:150709. 10.1016/j.bbrc.2024.150709. 39303526 10.1016/j.bbrc.2024.150709

[308] Wang T, Yao W, Li J, He Q, Shao Y, Huang F. Acetyl-CoA from inflammation-induced fatty acids oxidation promotes hepatic malate-aspartate shuttle activity and glycolysis. Am J Physiol Endocrinol Metab. 2018;315(4):E496-e510. 10.1152/ajpendo.00061.2018. 29763372 10.1152/ajpendo.00061.2018

[309] Fang Y, Zhan Y, Xie Y, Du S, Chen Y, Zeng Z, et al. Integration of glucose and cardiolipin anabolism confers radiation resistance of HCC. Hepatology. 2022;75(6):1386–401. 10.1002/hep.32177. 34580888 10.1002/hep.32177 PMC9299851

[310] Benichou E, Seffou B, Topçu S, Renoult O, Lenoir V, Planchais J, et al. The transcription factor ChREBP orchestrates liver carcinogenesis by coordinating the PI3K/AKT signaling and cancer metabolism. Nat Commun. 2024;15(1):1879. 10.1038/s41467-024-45548-w. 38424041 10.1038/s41467-024-45548-w PMC10904844

[311] Zhou X, Zhang J, Sun Y, Shen J, Sun B, Ma Q. Glutamine ameliorates liver steatosis via regulation of glycolipid metabolism and gut microbiota in high-fat diet-induced obese mice. J Agric Food Chem. 2023;71(42):15656–67. 10.1021/acs.jafc.3c05566. 37847053 10.1021/acs.jafc.3c05566

[312] Zhou P, Chang WY, Gong DA, Xia J, Chen W, Huang LY, et al. High dietary fructose promotes hepatocellular carcinoma progression by enhancing O-GlcNAcylation via microbiota-derived acetate. Cell Metab. 2023;35(11):1961-75.e6. 10.1016/j.cmet.2023.09.009. 37797623 10.1016/j.cmet.2023.09.009

[313] Zhang Q, Wei T, Jin W, Yan L, Shi L, Zhu S, et al. Deficiency in SLC25A15, a hypoxia-responsive gene, promotes hepatocellular carcinoma by reprogramming glutamine metabolism. J Hepatol. 2024;80(2):293–308. 10.1016/j.jhep.2023.10.024. 38450598 10.1016/j.jhep.2023.10.024

[314] Kim MJ, Choi YK, Park SY, Jang SY, Lee JY, Ham HJ, et al. PPARδ reprograms glutamine metabolism in Sorafenib-resistant HCC. Mol Cancer Res. 2017;15(9):1230–42. 10.1158/1541-7786.Mcr-17-0061. 28584024 10.1158/1541-7786.MCR-17-0061

[315] Kong Y, Wu M, Wan X, Sun M, Zhang Y, Wu Z, et al. Lipophagy-mediated cholesterol synthesis inhibition is required for the survival of hepatocellular carcinoma under glutamine deprivation. Redox Biol. 2023;63:102732. 10.1016/j.redox.2023.102732. 37150151 10.1016/j.redox.2023.102732 PMC10195991

[316] Jiang X, Peng J, Xie Y, Xu Y, Liu Q, Cheng C, et al. Oxoglutarate dehydrogenase-like inhibits the progression of hepatocellular carcinoma by inducing DNA damage through non-canonical function. Cell Death Differ. 2023;30(8):1931–42. 10.1038/s41418-023-01186-1. 37419985 10.1038/s41418-023-01186-1 PMC10406884

[317] Dai W, Xu L, Yu X, Zhang G, Guo H, Liu H, et al. OGDHL silencing promotes hepatocellular carcinoma by reprogramming glutamine metabolism. J Hepatol. 2020;72(5):909–23. 10.1016/j.jhep.2019.12.015. 31899205 10.1016/j.jhep.2019.12.015

[318] Lengauer F, Geisslinger F, Gabriel A, von Schwarzenberg K, Vollmar AM, Bartel K. A metabolic shift toward glycolysis enables cancer cells to maintain survival upon concomitant glutamine deprivation and V-ATPase inhibition. Front Nutr. 2023;10:1124678. 10.3389/fnut.2023.1124678. 37255933 10.3389/fnut.2023.1124678 PMC10225586

[319] Benzarti M, Neises L, Oudin A, Krötz C, Viry E, Gargiulo E, et al. PKM2 diverts glycolytic flux in dependence on mitochondrial one-carbon cycle. Cell Rep. 2024;43(3):113868. 10.1016/j.celrep.2024.113868. 38421868 10.1016/j.celrep.2024.113868

[320] Mancini C, Menegazzi G, Peppicelli S, Versienti G, Guasti D, Pieraccini G, et al. BCR::ABL1 expression in chronic myeloid leukemia cells in low oxygen is regulated by glutamine via CD36-mediated fatty acid uptake. Cancer Cell Int. 2025;25(1):176. 10.1186/s12935-025-03805-y. 40369538 10.1186/s12935-025-03805-y PMC12080266

[321] Li Z, Guan Y, Gao J, Zhu L, Zeng Z, Jing Q, et al. PPDPF-mediated regulation of BCAA metabolism enhances mTORC1 activity and drives cholangiocarcinoma progression. Oncogene. 2025;44(19):1415–33. 10.1038/s41388-025-03320-4. 40025229 10.1038/s41388-025-03320-4

[322] Zhou Z, Ye S, Chen J, Dai F, Chen L, Ye R, et al. ATF4 promotes glutaminolysis and glycolysis in colorectal cancer by transcriptionally inducing SLC1A5. Acta Biochim Biophys Sin (Shanghai). 2024;57(7):1093–105. 10.3724/abbs.2024226. 39696988 10.3724/abbs.2024226 PMC12383793

[323] Liu R, Li X, Xu J, Yan L, Hu K, Shi M, et al. The contrasting regulatory effects of valproic acid on ferroptosis and disulfidptosis in hepatocellular carcinoma. Theranostics. 2025;15(17):9091–113. 10.7150/thno.115661. 40963919 10.7150/thno.115661 PMC12439337

[324] Chu X, Zhong L, Dan W, Wang X, Zhang Z, Liu Z, et al. DNMT3A R882H mutation promotes acute leukemic cell survival by regulating glycolysis through the NRF2/NQO1 axis. Cell Signal. 2023;105:110626. 10.1016/j.cellsig.2023.110626. 36758683 10.1016/j.cellsig.2023.110626

[325] Li M, Thorne RF, Wang R, Cao L, Cheng F, Sun X, et al. Sestrin2-mediated disassembly of stress granules dampens aerobic glycolysis to overcome glucose starvation. Cell Death Discov. 2023;9(1):127. 10.1038/s41420-023-01411-3. 37059726 10.1038/s41420-023-01411-3 PMC10103035

[326] Chaneton B, Hillmann P, Zheng L, Martin ACL, Maddocks ODK, Chokkathukalam A, et al. Serine is a natural ligand and allosteric activator of pyruvate kinase M2. Nature. 2012;491(7424):458–62. 10.1038/nature11540. 23064226 10.1038/nature11540 PMC3894725

[327] Ye J, Mancuso A, Tong X, Ward PS, Fan J, Rabinowitz JD, et al. Pyruvate kinase M2 promotes de novo serine synthesis to sustain mTORC1 activity and cell proliferation. Proc Natl Acad Sci U S A. 2012;109(18):6904–9. 10.1073/pnas.1204176109. 22509023 10.1073/pnas.1204176109 PMC3345000

[328] Kurihara-Shimomura M, Sasahira T, Nakashima C, Kuniyasu H, Shimomura H, Kirita T. The multifarious functions of pyruvate kinase M2 in oral cancer cells. Int J Mol Sci. 2018. 10.3390/ijms19102907. 30257458 10.3390/ijms19102907 PMC6213602

[329] Dai W, Wang Z, Wang G, Wang QA, DeBerardinis R, Jiang L. FASN deficiency induces a cytosol-to-mitochondria citrate flux to mitigate detachment-induced oxidative stress. Cell Rep. 2023;42(8):112971. 10.1016/j.celrep.2023.112971. 37578864 10.1016/j.celrep.2023.112971 PMC10528718

[330] Chen J, Zhao L, Li W, Wang S, Li J, Lv Z, et al. Glutamine-driven metabolic reprogramming promotes CAR-T cell function through mTOR-SREBP2 mediated HMGCS1 upregulation in ovarian cancer. J Transl Med. 2025;23(1):803. 10.1186/s12967-025-06853-0. 40676647 10.1186/s12967-025-06853-0 PMC12273263

[331] Chatterjee S, Prashanth P, Rawat V, Ghosh Roy S. Regulation of lipid and serine metabolism by the oncogene c-Myc. Int Rev Cell Mol Biol. 2024;389:236–56. 10.1016/bs.ircmb.2024.03.005. 39396848 10.1016/bs.ircmb.2024.03.005

[332] Ma X, Zhang B, Yin X, Yang S, Lin Z, Yang Y, et al. CPT1A/HIF-1α positive feedback loop induced fatty acid oxidation metabolic pathway contributes to the L-ascorbic acid-driven angiogenesis in breast cancer. Breast Cancer Res. 2025;27(1):74. 10.1186/s13058-025-02039-0. 40355947 10.1186/s13058-025-02039-0 PMC12067761

[333] Sen U, Coleman C, Gandhi N, Jethalia V, Demircioglu D, Elliott A, et al. SCD1 inhibition blocks the AKT-NRF2-SLC7A11 pathway to induce lipid metabolism remodeling and ferroptosis priming in lung adenocarcinoma. Cancer Res. 2025;85(13):2485–503. 10.1158/0008-5472.Can-24-2745. 40198901 10.1158/0008-5472.CAN-24-2745 PMC12221774

[334] Kim MJ, Kim HS, Kang HW, Lee DE, Hong WC, Kim JH, et al. SLC38A5 modulates ferroptosis to overcome gemcitabine resistance in pancreatic cancer. Cells. 2023. 10.3390/cells12202509. 37887353 10.3390/cells12202509 PMC10605569

[335] Gotvaldová K, Špačková J, Novotný J, Baslarová K, Ježek P, Rossmeislová L, et al. BCAA metabolism in pancreatic cancer affects lipid balance by regulating fatty acid import into mitochondria. Cancer Metab. 2024;12(1):10. 10.1186/s40170-024-00335-5. 38532464 10.1186/s40170-024-00335-5 PMC10967191

[336] Yan Y, Wu X, Wang P, Zhang S, Sun L, Zhao Y, et al. Homocysteine promotes hepatic steatosis by activating the adipocyte lipolysis in a HIF1α-ERO1α-dependent oxidative stress manner. Redox Biol. 2020;37:101742. 10.1016/j.redox.2020.101742. 33045621 10.1016/j.redox.2020.101742 PMC7559542

[337] Jegatheesan P, Beutheu S, Ventura G, Sarfati G, Nubret E, Kapel N, et al. Effect of specific amino acids on hepatic lipid metabolism in fructose-induced non-alcoholic fatty liver disease. Clin Nutr. 2016;35(1):175–82. 10.1016/j.clnu.2015.01.021. 25736031 10.1016/j.clnu.2015.01.021

[338] Liu K, Qiu D, Liang X, Huang Y, Wang Y, Jia X, et al. Lipotoxicity-induced STING1 activation stimulates MTORC1 and restricts hepatic lipophagy. Autophagy. 2022;18(4):860–76. 10.1080/15548627.2021.1961072. 34382907 10.1080/15548627.2021.1961072 PMC9037528

[339] Simon J, Nuñez-García M, Fernández-Tussy P, Barbier-Torres L, Fernández-Ramos D, Gómez-Santos B, et al. Targeting hepatic glutaminase 1 ameliorates non-alcoholic steatohepatitis by restoring very-low-density lipoprotein triglyceride assembly. Cell Metab. 2020;31(3):605-22.e10. 10.1016/j.cmet.2020.01.013. 32084378 10.1016/j.cmet.2020.01.013 PMC7259377

[340] Ahmed EA, El-Derany MO, Anwar AM, Saied EM, Magdeldin S. Metabolomics and lipidomics screening reveal reprogrammed signaling pathways toward cancer development in non-alcoholic steatohepatitis. Int J Mol Sci. 2022. 10.3390/ijms24010210. 36613653 10.3390/ijms24010210 PMC9820351

[341] Yang XM, Wang XQ, Hu LP, Feng MX, Zhou YQ, Li DX, et al. Nucleolar HEAT repeat containing 1 up-regulated by the mechanistic target of rapamycin complex 1 signaling promotes hepatocellular carcinoma growth by dominating ribosome biogenesis and proteome homeostasis. Gastroenterology. 2023;165(3):629–46. 10.1053/j.gastro.2023.05.029. 37247644 10.1053/j.gastro.2023.05.029

[342] Sadria M, Seo D, Layton AT. The mixed blessing of AMPK signaling in cancer treatments. BMC Cancer. 2022;22(1):105. 10.1186/s12885-022-09211-1. 35078427 10.1186/s12885-022-09211-1 PMC8786626

[343] Dai X, Jiang C, Jiang Q, Fang L, Yu H, Guo J, et al. AMPK-dependent phosphorylation of the GATOR2 component WDR24 suppresses glucose-mediated mTORC1 activation. Nat Metab. 2023;5(2):265–76. 10.1038/s42255-022-00732-4. 36732624 10.1038/s42255-022-00732-4 PMC11070849

[344] Zhu Y, Wang A, Zhang S, Kim J, Xia J, Zhang F, et al. Paclitaxel-loaded ginsenoside Rg3 liposomes for drug-resistant cancer therapy by dual targeting of the tumor microenvironment and cancer cells. J Adv Res. 2023;49:159–73. 10.1016/j.jare.2022.09.007. 36167294 10.1016/j.jare.2022.09.007 PMC10334248

[345] Franczak MA, Krol O, Harasim G, Jedrzejewska A, Zaffaroni N, Granchi C, et al. Metabolic effects of new glucose transporter (GLUT-1) and lactate dehydrogenase-A (LDH-A) inhibitors against chemoresistant malignant mesothelioma. Int J Mol Sci. 2023. 10.3390/ijms24097771. 37175477 10.3390/ijms24097771 PMC10177874

[346] Hayashi M, Nakamura K, Harada S, Tanaka M, Kobayashi A, Saito H, et al. GLUT1 inhibition by BAY-876 induces metabolic changes and cell death in human colorectal cancer cells. BMC Cancer. 2025;25(1):716. 10.1186/s12885-025-14141-9. 40247224 10.1186/s12885-025-14141-9 PMC12004878

[347] Schumacher TJ, Iyer AV, Rumbley J, Ronayne CT, Mereddy VR. Exploring the impact of mitochondrial-targeting anthelmintic agents with GLUT1 inhibitor BAY-876 on breast cancer cell metabolism. BMC Cancer. 2024;24(1):1415. 10.1186/s12885-024-13186-6. 39550554 10.1186/s12885-024-13186-6 PMC11568538

[348] Miller ZA, Muthuswami S, Mueller A, Ma RZ, Sywanycz SM, Naik A, et al. GLUT1 inhibitor BAY-876 induces apoptosis and enhances anti-cancer effects of bitter receptor agonists in head and neck squamous carcinoma cells. Cell Death Discov. 2024;10(1):339. 10.1038/s41420-024-02106-z. 39060287 10.1038/s41420-024-02106-z PMC11282258

[349] Komza M, Khatun J, Gelles JD, Trotta AP, Abraham-Enachescu I, Henao J, et al. Metabolic adaptations to acute glucose uptake inhibition converge upon mitochondrial respiration for leukemia cell survival. Cell Commun Signal. 2025;23(1):47. 10.1186/s12964-025-02044-y. 39863913 10.1186/s12964-025-02044-y PMC11762851

[350] Mohite P, Lokwani DK, Sakle NS. Exploring the therapeutic potential of SGLT2 inhibitors in cancer treatment: integrating in silico and in vitro investigations. Naunyn Schmiedebergs Arch Pharmacol. 2024;397(8):6107–19. 10.1007/s00210-024-03021-x. 38416196 10.1007/s00210-024-03021-x

[351] Park LK, Lim KH, Volkman J, Abdiannia M, Johnston H, Nigogosyan Z, et al. Safety, tolerability, and effectiveness of the sodium-glucose cotransporter 2 inhibitor (SGLT2i) dapagliflozin in combination with standard chemotherapy for patients with advanced, inoperable pancreatic adenocarcinoma: a phase 1b observational study. Cancer Metab. 2023;11(1):6. 10.1186/s40170-023-00306-2. 37202813 10.1186/s40170-023-00306-2 PMC10193807

[352] Agnihotri S, Mansouri S, Burrell K, Li M, Mamatjan Y, Liu J, et al. Ketoconazole and Posaconazole Selectively Target HK2-expressing Glioblastoma Cells. Clin Cancer Res. 2019;25(2):844–55. 10.1158/1078-0432.Ccr-18-1854. 30322879 10.1158/1078-0432.CCR-18-1854 PMC8103287

[353] Xu S, Zhou T, Doh HM, Trinh KR, Catapang A, Lee JT, et al. An HK2 antisense oligonucleotide induces synthetic lethality in HK1(-)HK2(+) multiple myeloma. Cancer Res. 2019;79(10):2748–60. 10.1158/0008-5472.Can-18-2799. 30885978 10.1158/0008-5472.CAN-18-2799 PMC6522331

[354] Prasanna VK, Venkataramana NK, Dwarakanath BS, Santhosh V. Differential responses of tumors and normal brain to the combined treatment of 2-DG and radiation in glioablastoma. J Cancer Res Ther. 2009;5(1):S44–7. 10.4103/0973-1482.55141. 20009294 10.4103/0973-1482.55141

[355] Gupta N, Zhang B, Zhou Y, McCormack FX, Ingledue R, Robbins N, et al. Safety and Efficacy of Combined Resveratrol and Sirolimus in Lymphangioleiomyomatosis. Chest. 2023;163(5):1144–55. 10.1016/j.chest.2023.01.007. 36642366 10.1016/j.chest.2023.01.007 PMC10206511

[356] Peng YC, He ZJ, Yin LC, Pi HF, Jiang Y, Li KY, et al. Sanguinarine suppresses oral squamous cell carcinoma progression by targeting the PKM2/TFEB aix to inhibit autophagic flux. Phytomedicine. 2025;136:156337. 10.1016/j.phymed.2024.156337. 39729782 10.1016/j.phymed.2024.156337

[357] Yan L, Sun Y, Shi SS, Li Y, Zhang YF, Qu LZ, et al. Triclabendazole inhibits PKM2 nuclear localization and glycolysis by enhancing HDAC6-mediated deacetylation in lung cancer. J Transl Med. 2025;23(1):1001. 10.1186/s12967-025-06905-5. 40993625 10.1186/s12967-025-06905-5 PMC12461981

[358] Zhang J, Ouyang F, Gao A, Zeng T, Li M, Li H, et al. Esm1 enhances fatty acid synthesis and vascular mimicry in ovarian cancer by utilizing the PKM2-dependent warburg effect within the hypoxic tumor microenvironment. Mol Cancer. 2024;23(1):94. 10.1186/s12943-024-02009-8. 38720298 10.1186/s12943-024-02009-8 PMC11077861

[359] Wang B, Wang Z, Zhou Z, Liu G, Jiang Z, Zheng M, et al. Inhibition of 6-phosphogluconate dehydrogenase suppresses esophageal squamous cell carcinoma growth and enhances the anti-tumor effects of metformin via the AMPK/mTOR pathway. Mol Cancer. 2025;24(1):97. 10.1186/s12943-025-02302-0. 40140842 10.1186/s12943-025-02302-0 PMC11938747

[360] Gillis JL, Hinneh JA, Ryan NK, Irani S, Moldovan M, Quek LE, et al. A feedback loop between the androgen receptor and 6-phosphogluoconate dehydrogenase (6PGD) drives prostate cancer growth. Elife. 2021. 10.7554/eLife.62592. 34382934 10.7554/eLife.62592 PMC8416027

[361] Lin X, Xu Y, Bai E, Deng Y, Zhang W, Xue R, et al. 6-aminonicotinamide, a G6PD inhibitor, mitigates CAPS1 reduction mediated HCC metastasis via ERK and GSK3β signals. Neoplasia. 2025;70:101239. 10.1016/j.neo.2025.101239. 41082820 10.1016/j.neo.2025.101239 PMC12547465

[362] Liu Q, Shen J, Chen Y, Zhou J, Luo H, Deng J, et al. Phase II trial of radiotherapy plus Huachansu in elderly or chemotherapy-ineligible patients with locally advanced esophageal squamous cell carcinoma. Oncologist. 2025. 10.1093/oncolo/oyaf325. 41021440 10.1093/oncolo/oyaf325 PMC12573250

[363] Noble RA, Bell N, Blair H, Sikka A, Thomas H, Phillips N, et al. Inhibition of monocarboxyate transporter 1 by AZD3965 as a novel therapeutic approach for diffuse large B-cell lymphoma and Burkitt lymphoma. Haematologica. 2017;102(7):1247–57. 10.3324/haematol.2016.163030. 28385782 10.3324/haematol.2016.163030 PMC5566036

[364] Chatterjee P, Bhowmik D, Roy SS. A systemic analysis of monocarboxylate transporters in ovarian cancer and possible therapeutic interventions. Channels (Austin). 2023;17(1):2273008. 10.1080/19336950.2023.2273008. 37934721 10.1080/19336950.2023.2273008 PMC10631444

[365] Khajah MA, Khushaish S, Luqmani YA. The effect of lactate dehydrogenase inhibitors on proliferation, motility and invasion of breast cancer cells in vitro highlights a new role for lactate. Mol Med Rep. 2024;29(1). 10.3892/mmr.2023.13135. 10.3892/mmr.2023.13135 PMC10704548 37997856

[366] Di Magno L, Coluccia A, Bufano M, Ripa S, La Regina G, Nalli M, et al. Discovery of novel human lactate dehydrogenase inhibitors: Structure-based virtual screening studies and biological assessment. Eur J Med Chem. 2022;240:114605. 10.1016/j.ejmech.2022.114605. 35868126 10.1016/j.ejmech.2022.114605

[367] Hashimoto T, Ushikubo G, Arao N, Hatabi K, Tsubota K, Hosoi Y. Oxamate, an LDHA inhibitor, inhibits stemness, including EMT and high DNA repair ability, induces senescence, and exhibits radiosensitizing effects in glioblastoma cells. Int J Mol Sci. 2025. 10.3390/ijms26125710. 40565174 10.3390/ijms26125710 PMC12193169

[368] Dunbar EM, Coats BS, Shroads AL, Langaee T, Lew A, Forder JR, et al. Phase 1 trial of dichloroacetate (DCA) in adults with recurrent malignant brain tumors. Invest New Drugs. 2014;32(3):452–64. 10.1007/s10637-013-0047-4. 24297161 10.1007/s10637-013-0047-4 PMC4455946

[369] Mellinghoff IK, van den Bent MJ, Blumenthal DT, Touat M, Peters KB, Clarke J, et al. Vorasidenib in IDH1- or IDH2-mutant low-grade glioma. N Engl J Med. 2023;389(7):589–601. 10.1056/NEJMoa2304194. 37272516 10.1056/NEJMoa2304194 PMC11445763

[370] Harding JJ, Oh DY, Mercade TM, Goyal L, Varkaris A, Palmieri LJ, et al. Final results from a first-in-human phase 1 study of the dual isocitrate dehydrogenase (IDH) 1/2 inhibitor, LY3410738, in advanced solid tumors harboring IDH1 or IDH2 mutations. Clin Cancer Res. 2025. 10.1158/1078-0432.Ccr-25-0174. 41026608 10.1158/1078-0432.CCR-25-0174

[371] Arakawa Y, Saito R, Kanemura Y, Mishima K, Koriyama S, Narita Y, et al. Phase II study of safusidenib erbumine in patients with chemotherapy- and radiotherapy-naïve isocitrate dehydrogenase 1-mutated WHO grade 2 gliomas. Neuro Oncol. 2025. 10.1093/neuonc/noaf258. 41206766 10.1093/neuonc/noaf258 PMC13070488

[372] DiNardo CD, Hochhaus A, Frattini MG, Yee K, Zander T, Krämer A, et al. A phase 1 study of IDH305 in patients with IDH1(R132)-mutant acute myeloid leukemia or myelodysplastic syndrome. J Cancer Res Clin Oncol. 2023;149(3):1145–58. 10.1007/s00432-022-03983-6. 35353219 10.1007/s00432-022-03983-6 PMC11798246

[373] Woods A, Norsworthy KJ, Wang X, Vallejo J, Chiu Yuen Chow E, Li RJ, et al. FDA approval summary: ivosidenib in combination with azacitidine for treatment of patients with newly diagnosed acute myeloid leukemia with an IDH1 mutation. Clin Cancer Res. 2024;30(7):1226–31. 10.1158/1078-0432.Ccr-23-2234. 38010220 10.1158/1078-0432.CCR-23-2234 PMC10984783

[374] Mahalingam D, Harb W, Patnaik A, Bullock A, Watnick RS, Vincent MY, et al. First-in-human phase I dose escalation trial of the first-in-class tumor microenvironment modulator VT1021 in advanced solid tumors. Commun Med (Lond). 2024;4(1):10. 10.1038/s43856-024-00433-x. 38218979 10.1038/s43856-024-00433-x PMC10787778

[375] Chen J, Yu X, Yang G, Chen X, Gong C, Han L, et al. Combined blockade of lipid uptake and synthesis by CD36 inhibitor and SCD1 siRNA is beneficial for the treatment of refractory prostate cancer. Adv Sci. 2025;12(8):e2412244. 10.1002/advs.202412244. 10.1002/advs.202412244 PMC11848597 39736148

[376] Velez BC, Petrella CP, DiSalvo KH, Cheng K, Kravtsov R, Krasniqi D, et al. Combined inhibition of ACLY and CDK4/6 reduces cancer cell growth and invasion. Oncol Rep. 2023;49(2). 10.3892/or.2022.8469. 10.3892/or.2022.8469 PMC9827262 36562384

[377] Li R, Li Y, Song Z, Gu Y, Jiao X, Wan C, et al. A Graphene-Based Lipid Modulation Nanoplatform for Synergetic Lipid Starvation/Chemo/Photothermal Therapy of Oral Squamous Cell Carcinoma. Int J Nanomedicine. 2024;19:11235–55. 10.2147/ijn.S478308. 39524917 10.2147/IJN.S478308 PMC11545731

[378] Zhang X, Xu Y, Li S, Qin Y, Zhu G, Zhang Q, et al. SIRT2-mediated deacetylation of ACLY promotes the progression of oesophageal squamous cell carcinoma. J Cell Mol Med. 2024;28(6):e18129. 10.1111/jcmm.18129. 38426936 10.1111/jcmm.18129 PMC10906381

[379] Saisomboon S, Kariya R, Mahalapbutr P, Insawang T, Sawanyawisuth K, Cha’on U, et al. Augmented global protein acetylation diminishes cell growth and migration of cholangiocarcinoma cells. Int J Mol Sci. 2024. 10.3390/ijms251810170. 39337655 10.3390/ijms251810170 PMC11432552

[380] Jiang M, Xu L, Lin W, Liu W, Zhang Y, Wang H, et al. LncRNA CRCMSL interferes in phospholipid unsaturation to suppress colorectal cancer progression via reducing membrane fluidity. J Adv Res. 2025. 10.1016/j.jare.2025.02.003. 39921055 10.1016/j.jare.2025.02.003 PMC12684916

[381] Feng M, Gong W, Zhu X, Zhu J, Hu J, Xu W, et al. Covalent binding of 5-tetradecyloxy-2-furoic acid (TOFA) and c(RGDfK) and its co-delivery with Lipusu, a novel synergistic strategy to inhibit the proliferation of nasopharyngeal cancer. Eur J Pharm Sci. 2025;209:107092. 10.1016/j.ejps.2025.107092. 40228725 10.1016/j.ejps.2025.107092

[382] Menendez JA, Cuyàs E, Encinar JA, Vander Steen T, Verdura S, Llop-Hernández À, et al. Fatty acid synthase (FASN) signalome: A molecular guide for precision oncology. Mol Oncol. 2024;18(3):479–516. 10.1002/1878-0261.13582. 38158755 10.1002/1878-0261.13582 PMC10920094

[383] Almeida LY, Moreira FDS, Santos G, Cuadra Zelaya FJM, Ortiz CA, Agostini M, et al. FASN inhibition sensitizes metastatic OSCC cells to cisplatin and paclitaxel by downregulating cyclin B1. Oral Dis. 2023;29(2):649–60. 10.1111/odi.14017. 34510641 10.1111/odi.14017

[384] Sardesai SD, Thomas A, Gallagher C, Lynce F, Ottaviano YL, Ballinger TJ, et al. Inhibiting fatty acid synthase with omeprazole to improve efficacy of neoadjuvant chemotherapy in patients with operable TNBC. Clin Cancer Res. 2021;27(21):5810–7. 10.1158/1078-0432.Ccr-21-0493. 34400413 10.1158/1078-0432.CCR-21-0493

[385] Polonio-Alcalá E, Ausellé-Bosch S, Riesco-Llach G, Novales P, Feliu L, Planas M, et al. Elucidating the Role of FASN in Lung Cancer Stem Cells in Sensitive and Resistant EGFR-Mutated Non-Small Cell Lung Cancer Cells. Lung Cancer (Auckl). 2025;16:57–72. 10.2147/lctt.S512936. 40452758 10.2147/LCTT.S512936 PMC12126118

[386] Steen TV, Espinoza I, Duran C, Casadevall G, Serrano-Hervás E, Cuyàs E, et al. Fatty acid synthase (FASN) inhibition cooperates with BH3 mimetic drugs to overcome resistance to mitochondrial apoptosis in pancreatic cancer. Neoplasia. 2025;62:101143. 10.1016/j.neo.2025.101143. 39999714 10.1016/j.neo.2025.101143 PMC11908614

[387] Bandyopadhyay D, Tran ET, Patel RA, Luetzen MA, Cho K, Shriver LP, et al. Momordicine-I suppresses head and neck cancer growth by modulating key metabolic pathways. Cell Commun Signal. 2024;22(1):597. 10.1186/s12964-024-01951-w. 39696286 10.1186/s12964-024-01951-w PMC11657802

[388] Dembitz V, Lawson H, Burt R, Natani S, Philippe C, James SC, et al. Stearoyl-CoA desaturase inhibition is toxic to acute myeloid leukemia displaying high levels of the de novo fatty acid biosynthesis and desaturation. Leukemia. 2024;38(11):2395–409. 10.1038/s41375-024-02390-9. 39187579 10.1038/s41375-024-02390-9 PMC11518998

[389] Peng Y, Wu S, Xu Y, Ye X, Huang X, Gao L, et al. Huangqi-Danshen decoction alleviates renal fibrosis through targeting SCD1 to modulate cGAS/STING signaling. J Ethnopharmacol. 2025;342:119364. 10.1016/j.jep.2025.119364. 39832629 10.1016/j.jep.2025.119364

[390] Cai J, Ye Z, Hu Y, Ye L, Gao L, Wang Y, et al. Fatostatin induces ferroptosis through inhibition of the AKT/mTORC1/GPX4 signaling pathway in glioblastoma. Cell Death Dis. 2023;14(3):211. 10.1038/s41419-023-05738-8. 36966152 10.1038/s41419-023-05738-8 PMC10039896

[391] Wei G, Huang Y, Li W, Xie Y, Zhang D, Niu Y, et al. SREBF1-based metabolic reprogramming in prostate cancer promotes tumor ferroptosis resistance. Cell Death Discov. 2025;11(1):75. 10.1038/s41420-025-02354-7. 39988626 10.1038/s41420-025-02354-7 PMC11847930

[392] Wei G, Zhu H, Zhou Y, Pan Y, Yi B, Bai Y. Single-cell sequencing revealed metabolic reprogramming and its transcription factor regulatory network in prostate cancer. Transl Oncol. 2024;44:101925. 10.1016/j.tranon.2024.101925. 38447277 10.1016/j.tranon.2024.101925 PMC11391037

[393] Varney SD, Erkes DA, Mersky GL, Mustafa MU, Chua V, Chervoneva I, et al. Metabolic Inhibition Induces Pyroptosis in Uveal Melanoma. Mol Cancer Res. 2025;23(4):350–62. 10.1158/1541-7786.Mcr-24-0508. 39670827 10.1158/1541-7786.MCR-24-0508 PMC11961327

[394] Kawabata K, Takahashi T, Tanaka K, Kurokawa Y, Yamamoto K, Saito T, et al. Lipolysis-stimulated lipoprotein receptor promote lipid uptake and fatty acid oxidation in gastric cancer. Gastric Cancer. 2024;27(6):1258–72. 10.1007/s10120-024-01552-z. 39294388 10.1007/s10120-024-01552-z

[395] Hu A, Wang H, Xu Q, Pan Y, Jiang Z, Li S, et al. A novel CPT1A covalent inhibitor modulates fatty acid oxidation and CPT1A-VDAC1 axis with therapeutic potential for colorectal cancer. Redox Biol. 2023;68:102959. 10.1016/j.redox.2023.102959. 37977042 10.1016/j.redox.2023.102959 PMC10692921

[396] Shinoda S, Nakamura N, Inoko K, Sato-Dahlman M, Carmella S, Hecht S, et al. Inhibition of fatty acid binding protein suppresses pancreatic cancer progression and metastasis. Sci Rep. 2025;15(1):26084. 10.1038/s41598-025-11271-9. 40681747 10.1038/s41598-025-11271-9 PMC12274511

[397] Gouda MA, Voss MH, Tawbi H, Gordon M, Tykodi SS, Lam ET, et al. A phase I/II study of the safety and efficacy of telaglenastat (CB-839) in combination with nivolumab in patients with metastatic melanoma, renal cell carcinoma, and non-small-cell lung cancer. ESMO Open. 2025;10(5):104536. 10.1016/j.esmoop.2025.104536. 40359708 10.1016/j.esmoop.2025.104536 PMC12141888

[398] Guo H, Li W, Pan G, Wang C, Li D, Liu N, et al. The Glutaminase Inhibitor Compound 968 Exhibits Potent In vitro and In vivo Anti-tumor Effects in Endometrial Cancer. Anticancer Agents Med Chem. 2023;23(2):210–21. 10.2174/1871520622666220513163341. 35570522 10.2174/1871520622666220513163341

[399] Tanaka S, Hayashi S, Otsuka T, Kamiya T, Ishikawa K, Hara H. Inhibition of glutamine metabolism increases sensitivity to plasma-activated medium-induced cytotoxicity. Free Radic Res. 2024;58(3):170–9. 10.1080/10715762.2024.2332343. 38511644 10.1080/10715762.2024.2332343

[400] Lu Y, Zhou C, Li J, Liu L, Liu X, Shen L, et al. Radiation induces M2 polarization of glioma-associated macrophages via upregulation of glutamine synthetases. Int Immunopharmacol. 2025;154:114595. 10.1016/j.intimp.2025.114595. 40184814 10.1016/j.intimp.2025.114595

[401] Tatarskiy V, Chan W-K, Tan L, Khamidullina A, Mahmud I, Kumar SV, et al. The ASNS inhibitor ASX-173 potentiates L-asparaginase anticancer activity. 2025:2025.07.03.662851. 10.1101/2025.07.03.662851 %J bioRxiv.

[402] Pan Y, Suzuki T, Sakai K, Hirano Y, Ikeda H, Hattori A, et al. Bisabosqual A: a novel asparagine synthetase inhibitor suppressing the proliferation and migration of human non-small cell lung cancer A549 cells. Eur J Pharmacol. 2023;960:176156. 10.1016/j.ejphar.2023.176156. 38059445 10.1016/j.ejphar.2023.176156

[403] Szlosarek PW, Creelan BC, Sarkodie T, Nolan L, Taylor P, Olevsky O, et al. Pegargiminase Plus First-Line Chemotherapy in Patients With Nonepithelioid Pleural Mesothelioma: The ATOMIC-Meso Randomized Clinical Trial. JAMA Oncol. 2024;10(4):475–83. 10.1001/jamaoncol.2023.6789. 38358753 10.1001/jamaoncol.2023.6789 PMC10870227

[404] Menjivar RE, Nwosu ZC, Du W, Donahue KL, Hong HS, Espinoza C, et al. Arginase 1 is a key driver of immune suppression in pancreatic cancer. Elife. 2023. 10.7554/eLife.80721. 36727849 10.7554/eLife.80721 PMC10260021

[405] Akram HMB, Liu Y, Dong J, Zhao X, Wang L, Zhao W, et al. Discovery of W478, a novel SHMT2 inhibitor for the treatment of esophageal carcinoma. Bioorg Chem. 2025;165:109028. 10.1016/j.bioorg.2025.109028. 41014834 10.1016/j.bioorg.2025.109028

[406] Wilke AC, Doebele C, Zindel A, Lee KS, Rieke SA, Ceribelli M, et al. SHMT2 inhibition disrupts the TCF3 transcriptional survival program in Burkitt lymphoma. Blood. 2022;139(4):538–53. 10.1182/blood.2021012081. 34624079 10.1182/blood.2021012081 PMC8938936

[407] Ma Q, Gao S, Li C, Yao J, Xie Y, Jiang C, et al. Cuproptosis and serine metabolism blockade triggered by copper-based Prussian blue nanomedicine for enhanced tumor therapy. Small. 2025;21(5):e2406942. 10.1002/smll.202406942. 39676407 10.1002/smll.202406942

[408] Gong K, Huang Y, Zheng Y, Zhu Y, Hao W, Shi K. Preclinical efficacy of CBR-5884 against epithelial ovarian cancer cells by targeting the serine synthesis pathway. Discov Oncol. 2024;15(1):154. 10.1007/s12672-024-01013-0. 38733440 10.1007/s12672-024-01013-0 PMC11088592

[409] Zhou Q, Yin Y, Yu M, Gao D, Sun J, Yang Z, et al. GTPBP4 promotes hepatocellular carcinoma progression and metastasis via the PKM2 dependent glucose metabolism. Redox Biol. 2022;56:102458. 10.1016/j.redox.2022.102458. 36116159 10.1016/j.redox.2022.102458 PMC9483790

[410] Wu H, Pan L, Gao C, Xu H, Li Y, Zhang L, et al. Quercetin inhibits the proliferation of glycolysis-addicted HCC cells by reducing hexokinase 2 and Akt-mTOR pathway. Molecules. 2019. 10.3390/molecules24101993. 31137633 10.3390/molecules24101993 PMC6572074

[411] Li W, Hao J, Zhang L, Cheng Z, Deng X, Shu G. Astragalin reduces hexokinase 2 through increasing miR-125b to inhibit the proliferation of hepatocellular carcinoma cells in vitro and in vivo. J Agric Food Chem. 2017;65(29):5961–72. 10.1021/acs.jafc.7b02120. 28654261 10.1021/acs.jafc.7b02120

[412] Li M, Shao J, Guo Z, Jin C, Wang L, Wang F, et al. Novel mitochondrion-targeting copper(II) complex induces HK2 malfunction and inhibits glycolysis via Drp1-mediating mitophagy in HCC. J Cell Mol Med. 2020;24(5):3091–107. 10.1111/jcmm.14971. 31994339 10.1111/jcmm.14971 PMC7077532

[413] Li M, Zhang A, Qi X, Yu R, Li J. A novel inhibitor of PGK1 suppresses the aerobic glycolysis and proliferation of hepatocellular carcinoma. Biomed Pharmacother. 2023;158:114115. 10.1016/j.biopha.2022.114115. 36516697 10.1016/j.biopha.2022.114115

[414] Huang R, Zhang L, Jin J, Zhou Y, Zhang H, Lv C, et al. Bruceine D inhibits HIF-1α-mediated glucose metabolism in hepatocellular carcinoma by blocking ICAT/β-catenin interaction. Acta Pharm Sin B. 2021;11(11):3481–92. 10.1016/j.apsb.2021.05.009. 34900531 10.1016/j.apsb.2021.05.009 PMC8642446

[415] Hu B, Yu M, Ma X, Sun J, Liu C, Wang C, et al. IFNα potentiates anti-PD-1 efficacy by remodeling glucose metabolism in the hepatocellular carcinoma microenvironment. Cancer Discov. 2022;12(7):1718–41. 10.1158/2159-8290.Cd-21-1022. 35412588 10.1158/2159-8290.CD-21-1022

[416] Hsu CC, Wu LC, Hsia CY, Yin PH, Chi CW, Yeh TS, et al. Energy metabolism determines the sensitivity of human hepatocellular carcinoma cells to mitochondrial inhibitors and biguanide drugs. Oncol Rep. 2015;34(3):1620–8. 10.3892/or.2015.4092. 26133123 10.3892/or.2015.4092

[417] Yang G, Wang Y, Feng J, Liu Y, Wang T, Zhao M, et al. Aspirin suppresses the abnormal lipid metabolism in liver cancer cells via disrupting an NFκB-ACSL1 signaling. Biochem Biophys Res Commun. 2017;486(3):827–32. 10.1016/j.bbrc.2017.03.139. 28359761 10.1016/j.bbrc.2017.03.139

[418] Ding Z, Pan Y, Shang T, Jiang T, Lin Y, Yang C, et al. URI alleviates tyrosine kinase inhibitors-induced ferroptosis by reprogramming lipid metabolism in p53 wild-type liver cancers. Nat Commun. 2023;14(1):6269. 10.1038/s41467-023-41852-z. 37805657 10.1038/s41467-023-41852-z PMC10560259

[419] Wei Y, Tian C, Zhao Y, Liu X, Liu F, Li S, et al. MRG15 orchestrates rhythmic epigenomic remodelling and controls hepatic lipid metabolism. Nat Metab. 2020;2(5):447–60. 10.1038/s42255-020-0203-z. 32694659 10.1038/s42255-020-0203-z

[420] Su W, Wu S, Yang Y, Guo Y, Zhang H, Su J, et al. Phosphorylation of 17β-hydroxysteroid dehydrogenase 13 at serine 33 attenuates nonalcoholic fatty liver disease in mice. Nat Commun. 2022;13(1):6577. 10.1038/s41467-022-34299-1. 36323699 10.1038/s41467-022-34299-1 PMC9630536

[421] Du D, Qin M, Shi L, Liu C, Jiang J, Liao Z, et al. RNA binding motif protein 45-mediated phosphorylation enhances protein stability of ASCT2 to promote hepatocellular carcinoma progression. Oncogene. 2023;42(42):3127–41. 10.1038/s41388-023-02795-3. 37658192 10.1038/s41388-023-02795-3

[422] Chiu M, Tardito S, Pillozzi S, Arcangeli A, Armento A, Uggeri J, et al. Glutamine depletion by crisantaspase hinders the growth of human hepatocellular carcinoma xenografts. Br J Cancer. 2014;111(6):1159–67. 10.1038/bjc.2014.425. 25072259 10.1038/bjc.2014.425 PMC4453854

[423] Wei D, Chen D, Mou H, Chakraborty S, Wei B, Tan L, et al. Targeting glutamine metabolism with a novel Na+/K+-ATPase inhibitor RX108 in hepatocellular carcinoma. Mol Cancer Ther. 2023;22(6):693–705. 10.1158/1535-7163.Mct-22-0490. 36780187 10.1158/1535-7163.MCT-22-0490 PMC11817653

[424] Hu H, Ning S, Liu F, Zhang Z, Zeng W, Liu Y, et al. Hafnium Metal-Organic Framework-Based Glutamine Metabolism Disruptor For Potentiating Radio-Immunotherapy in MYC-Amplified Hepatocellular Carcinoma. ACS Appl Mater Interfaces. 2025;17(13):19367–81. 10.1021/acsami.4c21998. 40116395 10.1021/acsami.4c21998

[425] Yang C, Lee D, Zhang MS, Tse AP, Wei L, Bao MH, et al. Genome-wide CRISPR/Cas9 library screening revealed dietary restriction of glutamine in combination with inhibition of pyruvate metabolism as effective liver cancer treatment. Adv Sci (Weinh). 2022;9(34):e2202104. 10.1002/advs.202202104. 36310121 10.1002/advs.202202104 PMC9731711

[426] Missiaen R, Anderson NM, Kim LC, Nance B, Burrows M, Skuli N, et al. GCN2 inhibition sensitizes arginine-deprived hepatocellular carcinoma cells to senolytic treatment. Cell Metab. 2022;34(8):1151-67.e7. 10.1016/j.cmet.2022.06.010. 35839757 10.1016/j.cmet.2022.06.010 PMC9357184

[427] Ajmal I, Farooq MA, Duan Y, Yao J, Gao Y, Hui X, et al. Intrinsic ADRB2 inhibition improves CAR-T cell therapy efficacy against prostate cancer. Mol Ther. 2024;32(10):3539–57. 10.1016/j.ymthe.2024.08.028. 39228124 10.1016/j.ymthe.2024.08.028 PMC11489547

[428] Pucci G, Minafra L, Bravatà V, Calvaruso M, Turturici G, Cammarata FP, et al. Glut-3 gene knockdown as a potential strategy to overcome glioblastoma radioresistance. Int J Mol Sci. 2024. 10.3390/ijms25042079. 38396757 10.3390/ijms25042079 PMC10889562

[429] Li T, Gu Y, Xu B, Kuca K, Zhang J, Wu W. CircZBTB44 promotes renal carcinoma progression by stabilizing HK3 mRNA structure. Mol Cancer. 2023;22(1):77. 10.1186/s12943-023-01771-5. 37106446 10.1186/s12943-023-01771-5 PMC10134651

[430] Gunasekharan V, Lin HK, Marczyk M, Rios-Hoyo A, Campos GE, Shan NL, et al. Phosphoenolpyruvate carboxykinase-2 (PCK2) is a therapeutic target in triple-negative breast cancer. Breast Cancer Res Treat. 2024;208(3):657–71. 10.1007/s10549-024-07462-z. 39177932 10.1007/s10549-024-07462-z

[431] Silva L, Skiados N, Murugavel N, Cover K, Luna N, Gupta MK, et al. Efficient identification of new small molecules targeting succinate dehydrogenase in non-small cell lung cancer. Cancer Cell Int. 2025;25(1):362. 10.1186/s12935-025-04002-7. 41107962 10.1186/s12935-025-04002-7 PMC12535061

[432] Luo L, Wu X, Fan J, Dong L, Wang M, Zeng Y, et al. FBXO7 ubiquitinates PRMT1 to suppress serine synthesis and tumor growth in hepatocellular carcinoma. Nat Commun. 2024;15(1):4790. 10.1038/s41467-024-49087-2. 38839752 10.1038/s41467-024-49087-2 PMC11153525

[433] Pham TTM, Kim M, Nguyen TQN, Park JH, Kim JI, Seo JH, et al. Glycine decarboxylase regulates renal carcinoma progression via interferon stimulated gene factor 3-mediated pathway. Int J Biol Sci. 2025;21(2):772–88. 10.7150/ijbs.104458. 39781465 10.7150/ijbs.104458 PMC11705630

[434] Hu X, Chen W, Yang K, Zhu C, Li Z, Zheng D, et al. TCF19/CDKN2A regulates glycolysis and macrophage M2 polarization for osteosarcoma progression. FASEB J. 2025;39(7):e70519. 10.1096/fj.202401343RRR. 40193126 10.1096/fj.202401343RRR

[435] Wang Z, Wang C, Zhao S, Gu C. Unraveling the role of GPCR signaling in metabolic reprogramming and immune microenvironment of lung adenocarcinoma: a multi-omics study with experimental validation. Front Immunol. 2025;16:1606125. 10.3389/fimmu.2025.1606125. 40547013 10.3389/fimmu.2025.1606125 PMC12179119

[436] Baiskhanova D, Menzel M, Geismann C, Röcken C, Beitz E, Sebens S, et al. Transmembrane protease serine 11B modulates lactate transport through SLC16A1 in pancreatic ductal adenocarcinoma-a functional link to phenotype heterogeneity. Int J Mol Sci. 2025. 10.3390/ijms26115398. 40508207 10.3390/ijms26115398 PMC12155430

[437] Najumudeen AK, Ceteci F, Fey SK, Hamm G, Steven RT, Hall H, et al. The amino acid transporter SLC7A5 is required for efficient growth of KRAS-mutant colorectal cancer. Nat Genet. 2021;53(1):16–26. 10.1038/s41588-020-00753-3. 33414552 10.1038/s41588-020-00753-3

[438] Zhao Y, Wang Y, Miao Z, Liu Y, Yang Q. c-Myc protects hepatocellular carcinoma cell from ferroptosis induced by glutamine deprivation via upregulating GOT1 and Nrf2. Mol Biol Rep. 2023;50(8):6627–41. 10.1007/s11033-023-08495-1. 37358765 10.1007/s11033-023-08495-1

[439] Huang Y, Meng F, Zeng T, Thorne RF, He L, Zha Q, et al. IFRD1 promotes tumor cells “low-cost” survival under glutamine starvation via inhibiting histone H1.0 nucleophagy. Cell Discov. 2024;10(1):57. 10.1038/s41421-024-00668-x. 38802351 10.1038/s41421-024-00668-x PMC11130292

[440] Garcia CR, Highsmith K, Knight S, Andrade IP, Leung CH, Puduvalli V, et al. Single center experience of IDH inhibitors in recurrent high-grade gliomas. J Neurooncol. 2025;175(2):837–44. 10.1007/s11060-025-05183-x. 40748516 10.1007/s11060-025-05183-x

[441] Shen Y, Wang H, Guo D, Liu J, Sun J, Chen N, et al. Dual asparagine-depriving nanoparticles against solid tumors. Nat Commun. 2025;16(1):5675. 10.1038/s41467-025-60798-y. 40593710 10.1038/s41467-025-60798-y PMC12215482

[442] Wang J, Zhang Q, Fu H, Han Y, Li X, Zou Q, et al. ASCT2 regulates fatty acid metabolism to trigger glutamine addiction in basal-like breast cancer. Cancers (Basel). 2024. 10.3390/cancers16173028. 39272886 10.3390/cancers16173028 PMC11394221

[443] Xu Y, Yu Z, Fu H, Guo Y, Hu P, Shi J. Dual inhibitions on glucose/glutamine metabolisms for nontoxic pancreatic cancer therapy. ACS Appl Mater Interfaces. 2022;14(19):21836–47. 10.1021/acsami.2c00111. 35512029 10.1021/acsami.2c00111

[444] Lee CH, Motzer R, Emamekhoo H, Matrana M, Percent I, Hsieh JJ, et al. Telaglenastat plus Everolimus in advanced renal cell carcinoma: a randomized, double-blinded, placebo-controlled, phase II ENTRATA trial. Clin Cancer Res. 2022;28(15):3248–55. 10.1158/1078-0432.Ccr-22-0061. 35576438 10.1158/1078-0432.CCR-22-0061 PMC10202043

[445] Meric-Bernstam F, Tannir NM, Iliopoulos O, Lee RJ, Telli ML, Fan AC, et al. Telaglenastat plus Cabozantinib or Everolimus for advanced or metastatic renal cell carcinoma: an open-label phase I trial. Clin Cancer Res. 2022;28(8):1540–8. 10.1158/1078-0432.Ccr-21-2972. 35140121 10.1158/1078-0432.CCR-21-2972 PMC9164172

[446] Lin X, Liu Z, Zhou K, Li Y, Huang G, Zhang H, et al. Intratumoral and peritumoral PET/CT-based radiomics for non-invasively and dynamically predicting immunotherapy response in NSCLC. Br J Cancer. 2025;132(6):558–68. 10.1038/s41416-025-02948-z. 39930148 10.1038/s41416-025-02948-z PMC11920075

[447] Wang Q, Li M, Chen C, Xu L, Fu Y, Xu J, et al. Glucose homeostasis controls N-acetyltransferase 10-mediated ac4C modification of HK2 to drive gastric tumorigenesis. Theranostics. 2025;15(6):2428–50. 10.7150/thno.104310. 39990211 10.7150/thno.104310 PMC11840738

[448] Yuan H, Chen X, Zhao X, Dai M, Liu Y, Han J, et al. A head-to-head comparison of [(68)Ga]Ga-DOTA-FGFR1 and [(18)F]FDG PET/CT in the diagnosis of lung cancer. Eur J Nucl Med Mol Imaging. 2025;52(3):979–92. 10.1007/s00259-024-06976-4. 39516377 10.1007/s00259-024-06976-4

[449] Guo W, Xu W, Meng T, Fan C, Fu H, Pang Y, et al. FAP-targeted PET/CT imaging in patients with breast cancer from a prospective bi-center study: insights into diagnosis and clinic management. Eur J Nucl Med Mol Imaging. 2025;52(7):2317–34. 10.1007/s00259-025-07108-2. 39883140 10.1007/s00259-025-07108-2

[450] Huang S, Ren L, Beck JA, Phelps TE, Olkowski C, Ton A, et al. Exploration of imaging biomarkers for metabolically-targeted osteosarcoma therapy in a murine xenograft model. Cancer Biother Radiopharm. 2023;38(7):475–85. 10.1089/cbr.2022.0090. 37253167 10.1089/cbr.2022.0090 PMC10623067

[451] Storey CM, Altai M, Bicak M, Veach DR, Lückerath K, Adrian G, et al. Quantitative In Vivo Imaging of the Androgen Receptor Axis Reveals Degree of Prostate Cancer Radiotherapy Response. Mol Cancer Res. 2023;21(4):307–15. 10.1158/1541-7786.Mcr-22-0736. 36608299 10.1158/1541-7786.MCR-22-0736 PMC10355285

[452] Beinat C, Haywood T, Chen YS, Patel CB, Alam IS, Murty S, et al. The utility of [(18)F]DASA-23 for molecular imaging of prostate cancer with positron emission tomography. Mol Imaging Biol. 2018;20(6):1015–24. 10.1007/s11307-018-1194-y. 29736561 10.1007/s11307-018-1194-y

[453] Nakayama S, Nishio J, Aoki M, Koga K, Nabeshima K, Yamamoto T. GLUT-1 expression is helpful to distinguish myxofibrosarcoma from nodular fasciitis. Histol Histopathol. 2023;38(1):47–51. 10.14670/HH-18-490. 35792526 10.14670/HH-18-490

[454] Tang Y, Li W, Qiu L, Zhang X, Zhang L, Miyagishi M, et al. The p52-ZER6/G6PD axis alters aerobic glycolysis and promotes tumor progression by activating the pentose phosphate pathway. Oncogenesis. 2023;12(1):17. 10.1038/s41389-023-00464-4. 36977688 10.1038/s41389-023-00464-4 PMC10050210

[455] Yin Q, Yao Y, Ni J, Zhang Y, Wu J, Zeng H, et al. DLAT activates EMT to promote HCC metastasis by regulating GLUT1-mediated aerobic glycolysis. Mol Med. 2025;31(1):71. 10.1186/s10020-025-01125-5. 39979835 10.1186/s10020-025-01125-5 PMC11844032

[456] Wang W, Tian X, Yan L, Guan X, Dong B, Zhao M, et al. Identification of the γ-glutamyl cycle as a novel therapeutic target and 5-oxoproline as a new biomarker for diagnosing pancreatic cancer. Ann Med. 2023;55(2):2242247. 10.1080/07853890.2023.2242247. 37544888 10.1080/07853890.2023.2242247 PMC10405758

[457] Wu SL, Zha GY, Tian KB, Xu J, Cao MG. The metabolic reprogramming of gamma-aminobutyrate in oral squamous cell carcinoma. BMC Oral Health. 2024;24(1):418. 10.1186/s12903-024-04174-0. 38580938 10.1186/s12903-024-04174-0 PMC10996254

[458] Lin Z, Yang S, Qiu Q, Cui G, Zhang Y, Yao M, et al. Hypoxia-induced cysteine metabolism reprogramming is crucial for the tumorigenesis of colorectal cancer. Redox Biol. 2024;75:103286. 10.1016/j.redox.2024.103286. 39079386 10.1016/j.redox.2024.103286 PMC11340627

[459] Li K, Shi W, Song Y, Qin L, Zang C, Mei T, et al. Reprogramming of lipid metabolism in hepatocellular carcinoma resulting in downregulation of phosphatidylcholines used as potential markers for diagnosis and prediction. Expert Rev Mol Diagn. 2023;23(11):1015–26. 10.1080/14737159.2023.2254884. 37672012 10.1080/14737159.2023.2254884

[460] Zuo D, Xiao J, An H, Chen Y, Li J, Yang X, et al. Screening for lipid-metabolism-related genes and identifying the diagnostic potential of ANGPTL6 for HBV-related early-stage hepatocellular carcinoma. Biomolecules. 2022. 10.3390/biom12111700. 36421714 10.3390/biom12111700 PMC9687352

[461] O’Connor RS, Guo L, Ghassemi S, Snyder NW, Worth AJ, Weng L, et al. The CPT1a inhibitor, etomoxir induces severe oxidative stress at commonly used concentrations. Sci Rep. 2018;8(1):6289. 10.1038/s41598-018-24676-6. 29674640 10.1038/s41598-018-24676-6 PMC5908836

[462] Singh R, Gupta V, Kumar A, Singh K. 2-deoxy-D-glucose: a novel pharmacological agent for killing hypoxic tumor cells, oxygen dependence-lowering in Covid-19, and other pharmacological activities. Adv Pharmacol Pharm Sci. 2023;2023:9993386. 10.1155/2023/9993386. 36911357 10.1155/2023/9993386 PMC9998157

[463] Xu D, Jin J, Yu H, Zhao Z, Ma D, Zhang C, et al. Chrysin inhibited tumor glycolysis and induced apoptosis in hepatocellular carcinoma by targeting hexokinase-2. J Exp Clin Cancer Res. 2017;36(1):44. 10.1186/s13046-017-0514-4. 28320429 10.1186/s13046-017-0514-4 PMC5359903

[464] Hu B, Lin JZ, Yang XB, Sang XT. Aberrant lipid metabolism in hepatocellular carcinoma cells as well as immune microenvironment: a review. Cell Prolif. 2020;53(3):e12772. 10.1111/cpr.12772. 32003505 10.1111/cpr.12772 PMC7106960

[465] Yang J, He J, Feng Y, Xiang M. Obesity contributes to hepatocellular carcinoma development via immunosuppressive microenvironment remodeling. Front Immunol. 2023;14:1166440. 10.3389/fimmu.2023.1166440. 37266440 10.3389/fimmu.2023.1166440 PMC10231659

[466] Hu N, Li H, Tao C, Xiao T, Rong W. The role of metabolic reprogramming in the tumor immune microenvironment: mechanisms and opportunities for immunotherapy in hepatocellular carcinoma. Int J Mol Sci. 2024. 10.3390/ijms25115584. 38891772 10.3390/ijms25115584 PMC11171976

[467] Feng XC, Liu FC, Chen WY, Du J, Liu H. Lipid metabolism of hepatocellular carcinoma impacts targeted therapy and immunotherapy. World J Gastrointest Oncol. 2023;15(4):617–31. 10.4251/wjgo.v15.i4.617. 37123054 10.4251/wjgo.v15.i4.617 PMC10134209

[468] Takaichi M, Tachinami H, Takatsuka D, Yonesi A, Sakurai K, Rasul MI, et al. Targeting CD36-mediated lipid metabolism by selective inhibitor-augmented antitumor immune responses in oral cancer. Int J Mol Sci. 2024. 10.3390/ijms25179438. 39273384 10.3390/ijms25179438 PMC11395596

[469] Tzeng SF, Yu YR, Park J, von Renesse J, Hsiao HW, Hsu CH, et al. PLT012, a Humanized CD36-Blocking Antibody, Is Effective for Unleashing Antitumor Immunity Against Liver Cancer and Liver Metastasis. Cancer Discov. 2025;15(8):1676–96. 10.1158/2159-8290.Cd-24-1409. 40294022 10.1158/2159-8290.CD-24-1409 PMC7617665

[470] Wu S, Fukumoto T, Lin J, Nacarelli T, Wang Y, Ong D, et al. Targeting glutamine dependence through GLS1 inhibition suppresses ARID1A-inactivated clear cell ovarian carcinoma. Nat Cancer. 2021;2(2):189–200. 10.1038/s43018-020-00160-x. 34085048 10.1038/s43018-020-00160-x PMC8168620

[471] Babl N, Decking SM, Voll F, Althammer M, Sala-Hojman A, Ferretti R, et al. MCT4 blockade increases the efficacy of immune checkpoint blockade. J Immunother Cancer. 2023. 10.1136/jitc-2023-007349. 37880183 10.1136/jitc-2023-007349 PMC10603342

[472] Kelly CM, Qin LX, Whiting KA, Richards AL, Avutu V, Chan JE, et al. A phase II study of epacadostat and pembrolizumab in patients with advanced sarcoma. Clin Cancer Res. 2023;29(11):2043–51. 10.1158/1078-0432.Ccr-22-3911. 36971773 10.1158/1078-0432.CCR-22-3911 PMC10752758

[473] Fan L, Tian C, Yang W, Liu X, Dhungana Y, Yang W, et al. HKDC1 promotes liver cancer stemness under hypoxia through stabilizing β-catenin. Hepatology. 2025;81(6):1685–99. 10.1097/hep.0000000000001085. 39250463 10.1097/HEP.0000000000001085 PMC12077336

[474] Liu Q, Zhang X, Qi J, Tian X, Dovjak E, Zhang J, et al. Comprehensive profiling of lipid metabolic reprogramming expands precision medicine for HCC. Hepatology. 2025;81(4):1164–80. 10.1097/hep.0000000000000962. 38899975 10.1097/HEP.0000000000000962 PMC11902616

[475] Liu YJ, Li JX, Li JP, Hu YD, Ma ZB, Huang W, et al. Endoplasmic Reticulum Membrane Protein Complex Regulates Cancer Stem Cells and is Associated with Sorafenib Resistance in Hepatocellular Carcinoma. J Hepatocell Carcinoma. 2024;11:1519–39. 10.2147/jhc.S474343. 39139735 10.2147/JHC.S474343 PMC11321348

[476] Lu Y, Chan YT, Tan HY, Zhang C, Guo W, Xu Y, et al. Epigenetic regulation of ferroptosis via ETS1/miR-23a-3p/ACSL4 axis mediates sorafenib resistance in human hepatocellular carcinoma. J Exp Clin Cancer Res. 2022;41(1):3. 10.1186/s13046-021-02208-x. 34980204 10.1186/s13046-021-02208-x PMC8722264

[477] Zhou Y, Zhao H, Ren R, Zhou M, Zhang J, Wu Z, et al. GPAT3 is a potential therapeutic target to overcome sorafenib resistance in hepatocellular carcinoma. Theranostics. 2024;14(9):3470–85. 10.7150/thno.92646. 38948063 10.7150/thno.92646 PMC11209725

[478] Zhang S, Hu Y, Wu Z, Zhou X, Wu T, Li P, et al. Deficiency of carbamoyl phosphate synthetase 1 engenders radioresistance in hepatocellular carcinoma via deubiquitinating c-Myc. Int J Radiat Oncol Biol Phys. 2023;115(5):1244–56. 10.1016/j.ijrobp.2022.11.022. 36423742 10.1016/j.ijrobp.2022.11.022

[479] Law JH, Shao H, DasGupta R, Huang DQ. Developing risk stratification strategies and biomarkers for recurrent hepatocellular carcinoma. Clin Transl Med. 2025;15(8):e70410. 10.1002/ctm2.70410. 40741894 10.1002/ctm2.70410 PMC12311832

[480] Liu X, Gao X, Yang Y, Yang D, Guo Q, Li L, et al. Eva1a reverses lenvatinib resistance in hepatocellular carcinoma through regulating PI3K/AKT/p53 signaling axis. Apoptosis. 2024;29(7–8):1161–84. 10.1007/s10495-024-01967-0. 38743191 10.1007/s10495-024-01967-0

[481] Wang X, Du Q, Mai Q, Zou Q, Wang S, Lin X, et al. Targeting FASN enhances cisplatin sensitivity via SLC7A11-mediated ferroptosis in cervical cancer. Transl Oncol. 2025;56:102396. 10.1016/j.tranon.2025.102396. 40239242 10.1016/j.tranon.2025.102396 PMC12022685

[482] Ward AV, Riley D, Cosper KE, Finlay-Schultz J, Brechbuhl HM, Libby AE, et al. Lipid metabolic reprogramming drives triglyceride storage and variable sensitivity to FASN inhibition in endocrine-resistant breast cancer cells. Breast Cancer Res. 2025;27(1):32. 10.1186/s13058-025-01991-1. 40055794 10.1186/s13058-025-01991-1 PMC11889759

[483] Yang SM, Kim J, Lee JY, Lee JS, Lee JM. Regulation of glucose and glutamine metabolism to overcome cisplatin resistance in intrahepatic cholangiocarcinoma. BMB Rep. 2023;56(11):600–5. 10.5483/BMBRep.2023-0029. 37401237 10.5483/BMBRep.2023-0029 PMC10689087

[484] Ochi N, Miyake N, Takeyama M, Yamane H, Fukazawa T, Nagasaki Y, et al. The combined inhibition of SLC1A3 and glutaminase in osimertinib-resistant EGFR mutant cells. Biochim Biophys Acta Gen Subj. 2024;1868(10):130675. 10.1016/j.bbagen.2024.130675. 39059510 10.1016/j.bbagen.2024.130675

[485] Scheiblecker L, Klampfl T, Doma E, Nebenfuehr S, Torres-Quesada O, Strich S, et al. Cdk6 kinase inhibition unmasks metabolic dependencies in BCR::ABL1+ leukemia. Cell Death Dis. 2025;16(1):107. 10.1038/s41419-025-07434-1. 39966356 10.1038/s41419-025-07434-1 PMC11836434

